# Associations of Early Pregnancy Metabolite Profiles with Gestational Blood Pressure Development

**DOI:** 10.3390/metabo12121169

**Published:** 2022-11-24

**Authors:** Sophia M. Blaauwendraad, Rama J. Wahab, Bas B. van Rijn, Berthold Koletzko, Vincent W. V. Jaddoe, Romy Gaillard

**Affiliations:** 1 The Generation R Study Group, Erasmus MC, University Medical Center, 3000 CA Rotterdam, The Netherlands; 2 Department of Pediatrics, Erasmus MC, University Medical Center, 3000 CA Rotterdam, The Netherlands; 3 Department of Gynecology and Obstetrics, Erasmus MC, University Medical Center, 3000 CA Rotterdam, The Netherlands; 4 Division of Metabolic and Nutritional Medicine, Dr. von Hauner Children’s Hospital, LMU—Ludwig-Maximilians Universität München, 80337 Munich, Germany

**Keywords:** metabolomics, gestational hypertensive disorders, pregnancy, phospholipids, phosphatidylcholines, non-esterified fatty acids, carnitines, amino-acids

## Abstract

Blood pressure development plays a major role in both the etiology and prediction of gestational hypertensive disorders. Metabolomics might serve as a tool to identify underlying metabolic mechanisms in the etiology of hypertension in pregnancy and lead to the identification of novel metabolites useful for the prediction of gestational hypertensive disorders. In a population-based, prospective cohort study among 803 pregnant women, liquid chromatography—mass spectrometry was used to determine serum concentrations of amino-acids, non-esterified fatty acids, phospholipids and carnitines in early pregnancy. Blood pressure was measured in each trimester of pregnancy. Information on gestational hypertensive disorders was obtained from medical records. Higher individual metabolite concentrations of the diacyl-phosphatidylcholines and acyl-lysophosphatidylcholines group were associated with higher systolic blood pressure throughout pregnancy (Federal Discovery Rate (FDR)-adjusted *p*-values < 0.05). Higher concentrations of one non-esterified fatty acid were associated with higher diastolic blood pressure throughout pregnancy (FDR-adjusted *p*-value < 0.05). Using penalized regression, we identified 12 individual early-pregnancy amino-acids, non-esterified fatty acids, diacyl-phosphatidylcholines and acyl-carnitines and the glutamine/glutamic acid ratio, that were jointly associated with larger changes in systolic and diastolic blood pressure from first to third trimester. These metabolites did not improve the prediction of gestational hypertensive disorders in addition to clinical markers. In conclusion, altered early pregnancy serum metabolite profiles mainly characterized by changes in non-esterified fatty acids and phospholipids metabolites are associated with higher gestational blood pressure throughout pregnancy within the physiological ranges. These findings are important from an etiological perspective and, after further replication, might improve the early identification of women at increased risk of gestational hypertensive disorders.

## 1. Introduction

Gestational hypertensive disorders are among the most common causes of maternal and fetal morbidity and mortality worldwide [1]. Gestational hypertension and preeclampsia are at the extreme end of the blood pressure spectrum in pregnancy. Already small increases in blood pressure across this spectrum, even within the physiological ranges, are associated with gestational hypertensive disorders and adverse birth outcomes [2,3,4]. Metabolic changes might play an important role in the pathophysiology of increased blood pressure in pregnancy, as women with suboptimal metabolic profiles, especially with obesity, diabetes, and dyslipidemia, are at higher risks of gestational hypertension and pre-eclampsia [5,6]. Furthermore, changes in early-pregnancy serum concentrations of amino-acids (AA), phospholipids (PL), and fatty acids have been associated with gestational hypertensive disorders [7,8,9,10,11,12,13]. Among non-pregnant populations, higher longitudinal blood pressure and hypertension are associated with alterations in metabolites from pathways of, among others, AA, lipids, glucose metabolism, nucleotides and peptides [14,15,16]. However, findings are non-consistent and studies are limited by small sample size [17]. Further identification of metabolic profiles associated with gestational hypertensive disorders is needed. Furthermore, no prior studies investigated the association of early-pregnancy metabolic profiles with blood pressure throughout pregnancy. Identifying early-pregnancy metabolites associated with increased blood pressure could be useful in the early prediction of gestational hypertensive disorders, enabling intensified monitoring and early interventions [8,9,11]. With the use of metabolomics, detailed characterization of metabolic mechanisms underlying hypertension in pregnancy can be obtained [3]. 

Therefore, in a subsample of a population-based prospective cohort study, we first examined the associations of early-pregnancy serum metabolites with blood pressure development throughout pregnancy. We used a targeted metabolomics approach for amino acids, carnitines, fatty acids and phospholipids. These metabolites have been associated with cardiovascular diseases, play an important role in lipid metabolism, oxidative processes, inflammatory responses and immunity, and might therefore be involved in the pathophysiology of blood pressure development throughout pregnancy and gestational hypertensive disorders [7,8,9,10,11,12,13]. Second, we explored whether identified metabolites improve the prediction of gestational hypertensive disorders in addition to standard clinical characteristics 

## 2. Methods

### 2.1. Study Design

This study was embedded in the Generation R Study, a population-based prospective cohort study from fetal life until adulthood in Rotterdam, The Netherlands. Study approval was obtained by the Medical Ethical Committee of the Erasmus MC, University Medical Center, Rotterdam (MEC 198.782/2001/31) [18]. Written informed consent was obtained from all mothers. A subgroup of 814 pregnant women that enrolled between 2001 and 2005 had early-pregnancy metabolomics analysis. All women were Dutch. We excluded non-singleton live births leading to a study sample of 804 women. We excluded one woman without information on blood pressure in at least one trimester, resulting in a study sample of 803 mother-child-pairs (Flowchart shown in Figure S1). 

### 2.2. Metabolomics Analysis

At enrollment in the study, maternal non-fasting blood samples were obtained at a mean gestational age of 13.0 weeks (standard deviation ± 1.7 weeks) by research nurses [18]. Blood samples were transported to the regional laboratory (STAR-MDC), spun and stored at −80 degrees Celsius within 4 h after collection [19]. They were transported on dry ice to the Division of Metabolic and Nutritional Medicine of the Dr. von Hauner Children’s Hospital in Munich, Germany. As described in detail previously, targeted metabolomics approach was used to determine the serum concentrations (µmol/L) of amino-acids (AA), non-esterified fatty acids (NEFA), phospholipids (PL) (including diacyl-phosphatidylcholines (PC.aa), acyl-alkyl-phosphatidylcholines (PC.ae), acyl-lysophosphatidylcholines (Lyso.PC.a), alkyl-lysophosphatidylcholines (Lyso.PC.e), sphingomyelins (SM)) and carnitines (Carn) (including free carnitine (Free Carn) and acyl-carnitines (Carn.a)) [19,20]. Detailed information is given in the Supplemental Text. In short, AA were analyzed with 1100 high-performance liquid chromatography (HPLC) system (Agilent, Waldbronn, Germany) coupled to an API2000 tandem mass spectrometer (MS/MS) (AB Sciex, Darmstadt, Germany). IUPAC-IUB Nomenclature was used for notation of AA.((JCBN) 1984). Non-esterified fatty acids, phospholipids and carnitines were measured with a 1200 SL HPLC system (Agilent, Waldbronn, Germany) coupled to a 4000 QTRAP tandem mass spectrometer (AB Sciex, Darmstadt, Germany) [21,22]. Automatic data processing was performed with the Analyst© Software, using automatic peak selection. Visual inspection of the correct peak selection and consistent peak integration was performed by analysts and technicians. The analytical technique used was able to determine the total number of total bonds, but not the position of the double bonds and the distribution of the carbon atoms between fatty acid side chains. The following notation was used for non-esterified fatty acids, phospholipids and acyl-carnitines: X:Y, where X denotes the length of the carbon chain, and Y the number of double bonds. The ‘a’ denotes an acyl chain bound to the backbone of an ester bond (‘acyl-’) and the ‘e’ represents an ether bond (‘alkyl-’) [19]. Six quality control (QC) samples per batch were consistently measured between study samples. Data quality control (QC) was based on thresholds of 25% and 35% for the intra- and inter-batch coefficients of variation respectively [19]. To correct for batch effects, metabolite concentrations were divided by the ratio of the intra-batch and inter-batch median of the QC samples. Metabolites and participants with more than 50% of missing values were excluded. Missing metabolite values of the remaining metabolites and participants were imputed using the Random Forest algorithm (R package missForest).

Individual metabolites were clustered in the following metabolite groups, based on their chemical structure: AA, NEFA, PC.aa, PC.ae, Lyso.PC.a, Lyso.PC.e, SM, free Carn and Carn.a [19]. As we expected Krebs cycle, inflammation, oxidative stress and lipid metabolism to be part of the pathophysiological mechanism of blood pressure changes in pregnancy and gestational hypertensive disorders, we computed the following metabolite ratios: asparagine/aspartic acid (Asn/Asp) and glutamine/glutamic acid (Gln/Glu) as indicators for anaplerosis or replenishing of Krebs cycle metabolites; NEFA.18:1/NEFA.18:0 and NEFA.16:1/NEFA.16:0 as markers of stearoyl-CoA desaturase-1 activity, which is associated with increased fat accumulation and reduced fatty acid oxidation; ΣPC.aa/ΣPC.ae, reflecting oxidative stress; ΣLyso.PC.a/ΣPC.aa, as a lipid biomarker of inflammation; (lyso.PC.a.C16:0 + lyso.PC.a.C18:0)/ΣPC.aa as a proinflammatory biomarker; (lyso.PC.a.C18:1 + lyso.PC.a.C18:2)/ΣPC.aa as an anti-inflammatory biomarker; Carn.a.C:16:0/free Carn as marker of Carn palmitoyl transferase-1 activity and Carn.a.C2:0/ Carn.a.C16:0 as markers of fatty acid β-oxidation [23,24,25,26,27,28].

### 2.3. Blood Pressure and Gestational Hypertensive Disorders

Systolic and diastolic blood pressure were measured in early pregnancy (median 12.8 weeks, 95% range 9.9, 16.9 weeks), mid pregnancy (median 20.4 weeks, 95% range 18.9, 22.5 weeks) and late pregnancy (median 30.2 weeks, 95% range 28.4, 32.5 weeks) using a validated Omron 907 automated digital oscillometric sphygmomanometer (OMRON Healthcare Europe BV, Hoofddorp, The Netherlands) [29]. Participants were seated in upright position. After a minimum of 5 min of relaxation, a cuff was placed around the non-dominant upper arm at the level of the heart. For further analysis, we used the mean of two blood pressure measurements with a 60-s interval. We used blood pressure in each trimester and calculated the blood pressure change from first to third trimester by subtracting first trimester systolic or diastolic blood pressure from the third trimester systolic or diastolic blood pressure, respectively. Steeper increases in blood pressure from first to third trimester are strongly associated with gestational hypertensive disorders [4,30]. Information about gestational hypertensive disorders was obtained from medical records [31]. Women suspected for gestational hypertensive disorders based on these records were cross-validated using hospital registries, and defined using criteria of the International Society for the Study of Hypertension in Pregnancy [31,32]. Gestational hypertension was defined as hypertension (blood pressure at least 140/90 mmHg), appearing after 20 weeks of gestational age in women who were previously normotensive. Preeclampsia was defined as hypertension after 20 weeks gestation in women who were previously normotensive, with concurrent proteinuria, defined as 2 or more dipstick readings of 2+ or greater, 1 catheter sample reading of 1+ or greater, or a 24-h urine collection containing at least 300 mg of protein). 

### 2.4. Covariates

We obtained information on maternal characteristics related to blood pressure. At enrollment, age was assessed, and weight and height were measured without shoes. We obtained information on parity (nulliparous or multiparous), educational level (finished primary school, secondary school or higher) and smoking status (non-smoking, smoked until pregnancy was known, continued smoking in pregnancy), folic acid supplementation use (yes/no), obstetric history of gestational hypertensive disorders (yes/no), preexisting hypertension (yes/no) and hypertensive disorders in first-degree family members (yes/no) through questionnaire. 

### 2.5. Statistical Analysis 

First, we square root transformed metabolites to obtain normal distributions [19]. We standardized the metabolites by dividing them by their respective standard deviation, to enable comparison between metabolites. Second, we aimed to identify metabolites that are involved in the etiology of higher blood pressure development in pregnancy. We examined the associations of early-pregnancy serum concentrations of the individual metabolites of the groups AA, NEFA, PC.aa, PC.ae, Lyso.PC.a, Lyso.PC.e, SM, free carnitine and Carn.a with first, second and third trimester systolic and diastolic blood pressure using linear regression models. Analyses were first only adjusted for gestational age in the basic model, and subsequently for sociodemographic and lifestyle factors in the adjusted model. We considered the adjusted model as the main model. Confounders were selected from previous literature and were defined in a Direct Acyclic Graph (Figure S2) and included: age, pre-pregnancy body mass index, educational level, parity, smoking and folic acid supplement use [33,34,35,36,37,38,39]. As family hypertension is closely related to gestational hypertensive disorders, we performed a separate analysis additionally adjusting for hypertensive disorders in first-degree family members [40]. Next, we performed a sensitivity analysis excluding women with preexisting hypertension. This way, we aimed to identify which associations of metabolite profiles with higher blood pressure were caused by preexisting hypertension rather than gestational hypertension. Benjamin–Hochberg Federal Discovery Rate (FDR)-corrected *p*-values were obtained to correct for multiple hypothesis testing, which is a widely used approach for multiple testing in omics studies [41]. We considered an FDR-adjusted *p*-value of <0.05 statistically significant. 

Next, we aimed to identify metabolites for the prediction of gestational hypertensive disorders. We used the outcome blood pressure changes from first to third trimester, as larger increases are associated with higher risks of gestational hypertension and preeclampsia. We did not use the outcomes gestational hypertension or pre-eclampsia due to lack of power for model development in our population-based study sample. We used a penalized regression method (lasso regression) to select a combination of individual metabolites that were jointly associated with systolic and diastolic blood pressure change from first to third trimester, in addition to the selected confounders (R package *glmnet*) [42]. Lasso regression allows the use of correlated variables and enables variable selection such that only important variables stay in the model using shrinkage. We chose this method above unsupervised analysis methods such as Principal Component Analysis and cluster analysis, as we aimed to identify individual metabolites rather than clusters, using regression models on the outcome blood pressure. After replication, a small group of individual metabolites would be most easily implemented in future screening of prediction studies or even clinical practice. The regression included all early-pregnancy metabolites in the model and the selected confounders which could not be penalized. The selected confounders were age, pre-pregnancy body-mass index, parity and smoking, as these have shown to be predictive for blood pressure changes in our study population [43]. We performed a 10-fold cross-validation and used the penalty parameter value yielding the smallest prediction error. Next, we examined whether the identified metabolite profiles were associated with the risk of gestational hypertensive disorders in our population. We used logistic regression models to construct (1) a clinical model, including age, pre-pregnancy body mass index, parity and smoking and (2) a metabolite model additionally including early-pregnancy serum metabolites selected from the penalized regression models [43]. We assessed model performance via Receiver Operating Characteristics (ROC) curves and calculated the area-under-the-curve (AUC) (R package ROC). To compare the model performance of the clinical models with the metabolite models, we used the test of DeLong et al. 

Missing values were imputed using multiple imputation by the fully conditional specification method and pooled results from 5 imputed datasets were reported [44]. The percentage of missing values for covariates ranged from 0.5% (educational level) to 14.9% (pre-pregnancy body mass index). All statistical tests were 2-sided and FDR-adjusted *p*-values for all analysis were presented. The analyses were performed using the Statistical Package for the Social Sciences version 25.0 (IBM Corp., Armonk, New York, NY, USA) and R Statistical Software version 4.1.0 (R Foundation for Statistical Computing, Vienna, Austria). 

## 3. Results

### 3.1. Population Characteristics

At enrollment, the mean maternal age was 31.4 years (standard deviation (±SD) 4.1 years), and the median pre-pregnancy body mass index was 22.6 kg/m^2^ (95% range 20.9, 25.2 kg/m^2^) (Table 1). Most women were nulliparous (61.0%), highly educated (63.3%) and never smoked in pregnancy (75.9%). The study population included 37 cases of gestational hypertension (4.8%) and 12 cases of preeclampsia (1.6%). 9 (1.1%) women had preexisting hypertension and 38 (4.7%) women had a previous pregnancy complicated by gestational hypertensive disorders. Median concentrations of early-pregnancy serum metabolite groups and individual metabolites obtained at a mean gestational age of 13.0 weeks (±SD 1.7 weeks) are shown in Table S1. 

### 3.2. Maternal Early-Pregnancy Metabolites and Gestational Blood Pressure

**Figure 1:**
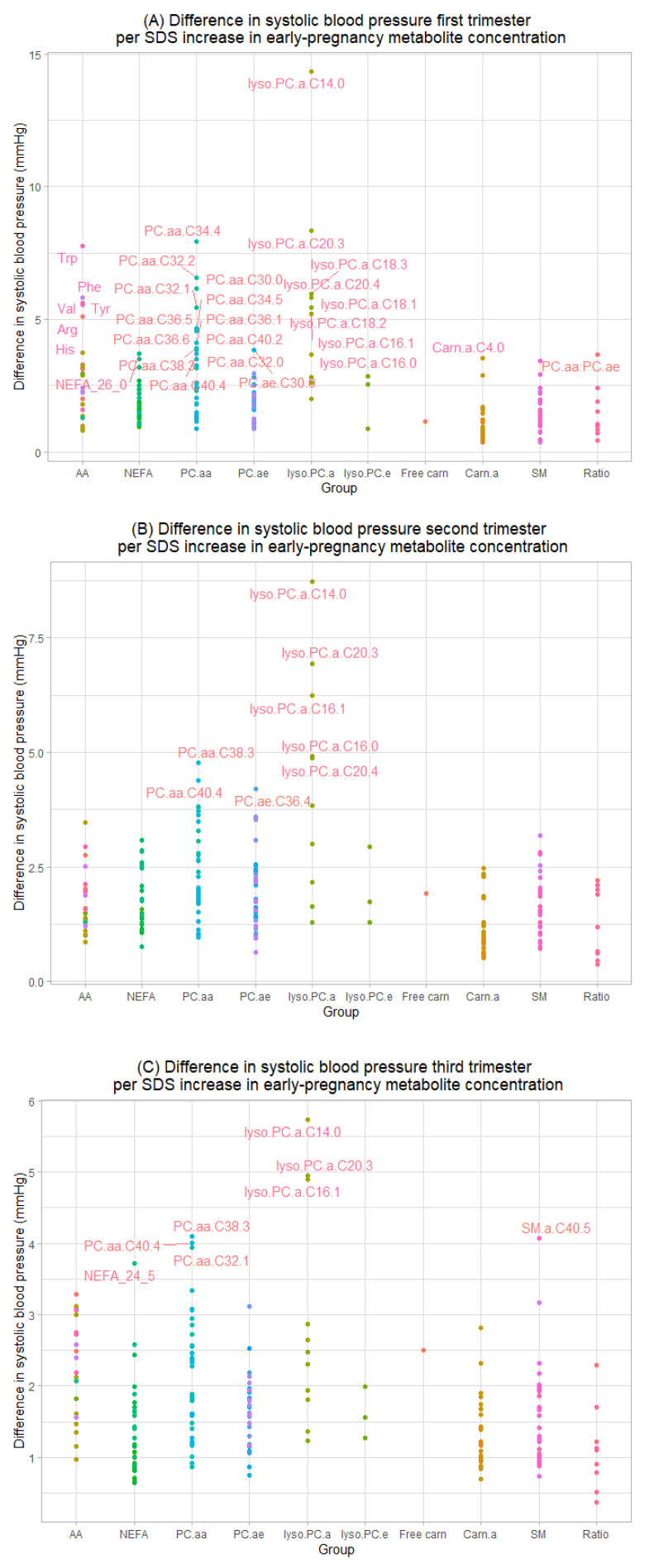
Associations of maternal serum early-pregnancy individual metabolites with systolic blood pressure in (**A**) early-, (**B**) mid- and (**C**) late pregnancy. Values represent absolute differences in blood pressure from linear regression models that reflect the difference in blood pressure (mmHg) per SDS increase in maternal early-pregnancy metabolite concentrations (μmol/L). Model includes gestational age at time of measurement, age, parity, pre-pregnancy body mass index, educational level, smoking and folic acid supplementation. Labeled values represent significant associations (FDR-adjusted *p*-values < 0.05). Corresponding numerical values are shown in Table S4. AA amino acids, NEFA non-esterified fatty acids, PC.aa diacyl-phosphatidylcholines, PC.ae acyl-alkyl-phosphatidylcholines, lyso.PC.a acyl-lysophosphatidylcholines, lyso.PC.e alkyl-lysophosphatidylcholines, Carn.a acyl-carnitines, SM sphingomyelins.

Figure 1 shows that higher early-pregnancy serum concentrations of five individual metabolites were associated with higher systolic blood pressure in all three trimester, namely PC.aa.C38.3, PC.aa.40.4, Lyso.PC.a.C14.0, Lyso.PC.a.C16.1 and Lyso.PC.a.20.3 (Table S4) (FDR-adjusted *p*-values < 0.05). The strongest associations were present for higher early pregnancy Lyso.PC.a.C14.0 and higher blood pressure in first and second trimester (14.69 mmHg (95% CI 5.76, 37.44) and 8.75 mmHg (95% CI 3.49, 21.93) increase in systolic blood pressure per SDS increase in metabolite concentration, respectively). In total, higher concentrations of 28 early-pregnancy serum metabolites of the groups AA, Carn.a, LysoPC.a, NEFA, PC.aa and PC.ae groups and of the metabolite ratio PC.aa:PC.ae were associated with higher systolic blood pressure in first trimester. Higher concentrations of eight Lyso.PC.a and PC.aa metabolites were associated with higher systolic blood pressure in second trimester, and of eight NEFA, Lyso.PC.a, PC.aa and SM metabolites with higher systolic blood pressure in third trimester (all FDR-adjusted *p*-values < 0.05). We found no associations of early-pregnancy serum concentrations of free Carn, PC.ae or Lyso.PC.e metabolites with systolic blood pressure in pregnancy.

**Figure 2:**
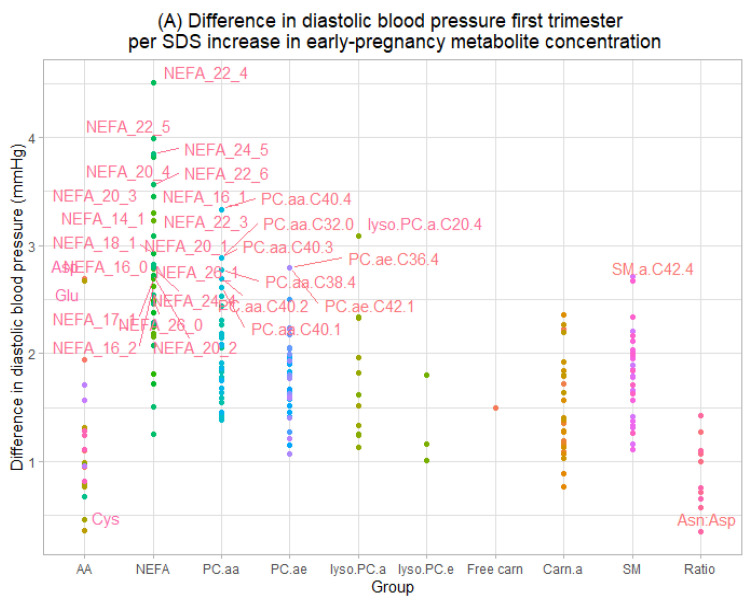

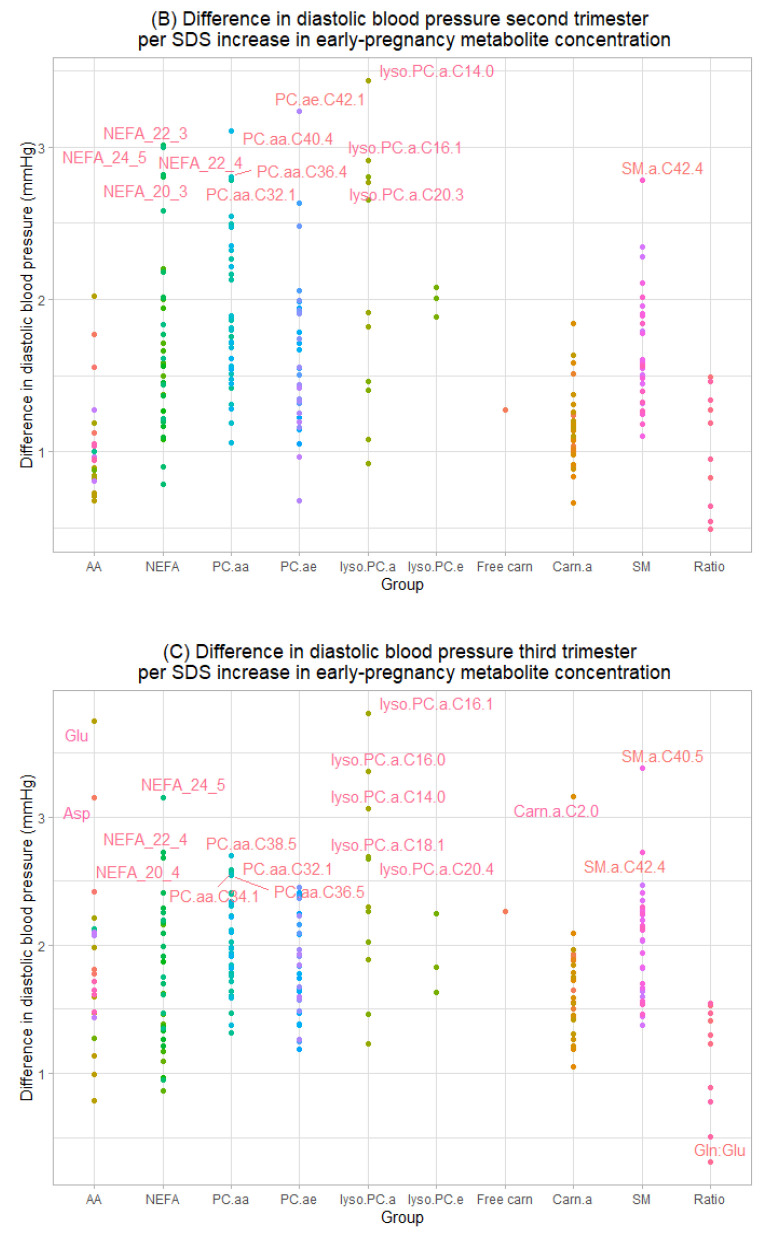
Associations of maternal serum early-pregnancy individual metabolites with diastolic blood pressure in (**A**) early-, (**B**) mid- and (**C**) late pregnancy. Values represent absolute differences in blood pressure from linear regression models that reflect the difference in blood pressure (mmHg) per SDS increase in maternal early-pregnancy metabolite concentrations (μmol/L). Model includes gestational age at time of measurement, age, parity, pre-pregnancy body mass index, educational level, smoking and folic acid supplementation. Labeled values represent significant associations (FDR-adjusted *p*-values < 0.05). Corresponding numerical values are shown in Table S5. AA amino acids, NEFA non-esterified fatty acids, PC.aa diacyl-phosphatidylcholines, PC.ae acyl-alkyl-phosphatidylcholines, lyso.PC.a acyl-lysophosphatidylcholines, lyso.PC.e alkyl-lysophosphatidylcholines, Carn.a acyl-carnitines, SM sphingomyelins.

Higher early-pregnancy serum concentrations of the individual metabolite NEFA.C24.5 were associated with higher diastolic blood pressure in all three trimesters (FDR-adjusted *p*-value < 0.05) (Figure 2, Table S5). In total, higher concentrations of 31 individual metabolites of the AA, Lyso.PC.a, NEFA, PC.aa, PC.ae and SM group and the metabolite ratio Asn/Asp were associated with higher diastolic blood pressure in first trimester. Higher concentrations of 12 individual Lyso.PC.a, NEFA, PC.aa and PC.ae metabolites were associated with higher diastolic blood pressure in second trimester and higher concentrations of 16 individual AA, Carn.a, Lyso.PC.a, NEFA, PC.aa and SM metabolites and the metabolite ratio Gln/Glu were associated with higher blood pressure in third trimester (all FDR-adjusted *p*-values < 0.05). We found no associations of early-pregnancy serum concentrations of Lyso.PC.e metabolites or free carnitine with diastolic blood pressure in pregnancy. Additionally adjusting for family history of hypertension in general yielded similar results to the main analysis (Tables S6 and S7). Only the associations of early-pregnancy lyso.PC.a.C16.0 with first trimester systolic blood pressure and of NEFA.C26.0 and PC.aa.C40.1 with first trimester diastolic blood pressure were no longer significant. When we performed a sensitivity analysis excluding women with pre-existing hypertension, we observed similar findings to the main analysis (Tables S8 and S9).

### 3.3. Maternal Early-Pregnancy Metabolites and Prediction of Higher Blood Pressure

**Figure 3:**
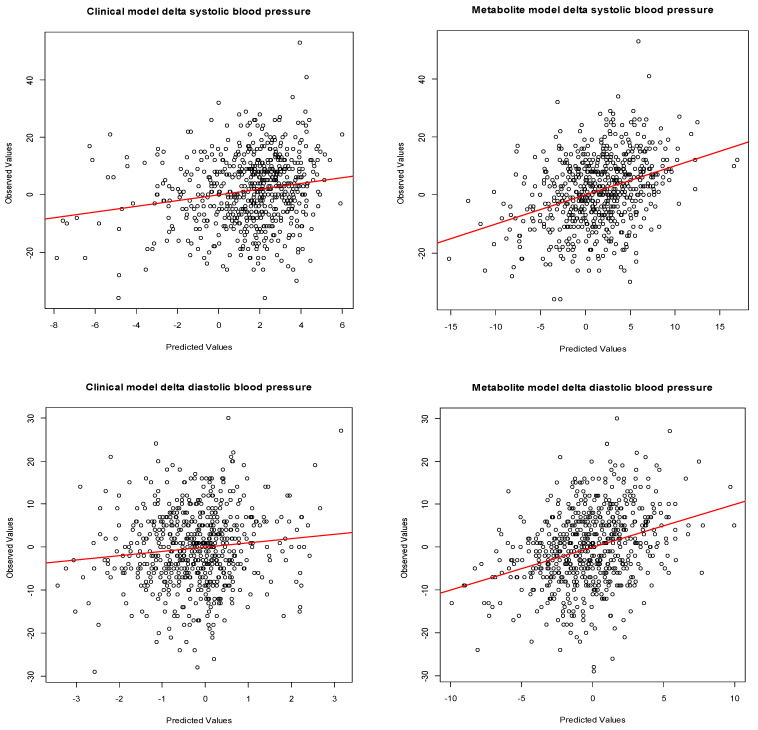
Observed versus predicted values for the linear regression models on diastolic and systolic blood pressure change from first to third trimester. Scatterplot represents observed and predicted values for changes in systolic and diastolic blood pressure from first to third trimester. Predicted values were obtained from linear regression models. Clinical models include maternal age, pre-pregnancy BMI, parity and smoking. Metabolite model includes maternal age, pre-pregnancy BMI, parity, smoking, and arginine, asparagine, glycine, lysine, tryptophan, NEFA_18_0, NEFA_26_0, PC.aa.C34.4, PC.ae.C36.5, SM.a.C37.1, SM.a.C38.2, SM.a.C39.2, SM.a.C41.1, SM.a.C43.2, Carn.a.C16.1, Carn.a.C16.2 and asparagine/aspartic acid ratio.

Using lasso penalized regression models, a combination of 45 individual early-pregnancy metabolites and 1 metabolite ratio were selected on the outcome systolic blood pressure change from first to third trimester, and a combination of 38 metabolites and 1 metabolite ratio on the outcome diastolic blood pressure change (Tables S10 and S11). In both models, the strongest effects were of Carn.a metabolites. In total, 12 metabolites and 1 metabolite ratio were jointly selected in both models; those were arginine, asparagine, glycine, lysine, tryptophan, NEFA.C18.0, NEFA.C26.0, PC.aa.C34.4, PC.ae.C36.5, SM.a.C37.1, SM.a.38.3, SM.a.C39.2, SM.a.C41.1, SM.a.C34.2, Carn.a.C16.1, Carn.a.C16.2 and asparagine/aspartic acid. The explained variance of the metabolite model for systolic blood pressure changes from first to third trimester including the selected confounders and metabolites was 9.2% (SD residuals 11.3), which was higher than the explained variance of the clinical model (2.6%, SD residuals 11.9) (Table 2). The explained variance of the metabolite model for diastolic blood pressure changes from first to third trimester was 7.5% (SD residuals 8.3), which was also higher than the explained variance of the clinical model (0.6%, SD residuals 8.7). The observed versus predicted values of the models for the outcomes are shown in Figure 3. The metabolite model did not improve the prediction of gestational hypertensive disorders, as compared to the clinical model (AUC 0.78 (95% CI 0.70, 0.85) versus AUC 0.76 (95% CI 0.68, 0.82), *p*-value DeLong 0.516) (Figure S3).

## 4. Discussion

In this prospective cohort study, we observed that altered early-pregnancy PC.aa and Lyso.PC.a metabolites were associated with higher systolic blood pressure development throughout pregnancy, whereas higher concentrations of NEFA.24.5 were associated with a higher diastolic blood pressure throughout pregnancy. To a lesser extent, changes in several AA and Carn.a metabolite concentrations were associated with higher blood pressure in pregnancy. We identified 12 individual early-pregnancy AA, NEFA, PC.aa and Carn.a metabolites and 1 metabolite ratio representing reduced Krebs cycle anaplerosis, that were jointly associated with larger increase in diastolic and systolic blood pressure from first to third trimester. These metabolites improved the prediction of variation in blood pressure throughout pregnancy, but did not improve the prediction of gestational hypertensive disorders in addition to clinical markers.

### 4.1. Interpretation of Main Findings

Blood pressure development plays a major role in both the etiology and prediction of gestational hypertensive disorders. The identification of metabolic changes in early-pregnancy serum associated with higher blood pressure throughout pregnancy enables more insight into underlying metabolic changes and mechanisms of hypertension in pregnancy. 

Previous studies investigated the association of metabolite profiles and blood pressure in non-pregnant populations. A systematic review compared metabolite profiles of patients with essential hypertension with healthy controls [14]. Of the 19 metabolites that were identified in relation to essential hypertension, the metabolites that showed constant changing trends were valine, glycine, oleic acid, pyruvate and lactic acid. A targeted metabolomics study among 504 normotensive men concluded that higher concentrations of ceramide, triacylglycerol, total glycerolipids and oleic acid and lower cholesteryl ester were associated with higher continuous diastolic blood pressure change, but no associations were found with systolic blood pressure change [15]. An untargeted metabolomics study among 1249 participants identified 30 metabolites robustly associated with higher diastolic and systolic blood pressure, which included metabolites from metabolic pathways of among others amino acids, lipids, nucleotides and peptides [16]. In pregnant populations, several small studies focused on metabolite profiles and gestational hypertensive disorders [8,9,11]. As compared to controls, 30 pregnant women that developed early-onset preeclampsia had decreased early-pregnancy serum levels of glycose and pyruvate, increased levels of the amino acids alanine, glutamine, glycine, isoleucine, leucine, phenylalanine, serine, threonine, methionine, and increased choline and glycerol which are precursors in phospholipid, phosphatidylcholine and triacylglycerol synthesis [9]. In 59 women with late-onset pre-eclampsia as compared to controls, increased levels of early-pregnancy serum methyl histidine, leucine and valine, of carnitine, glucose, pyruvate and lactate and of oxidative stress marker 2-hydroxybutyric acid were found [11]. Together, those studies among non-pregnant populations and women with gestation hypertensive disorders suggest that changed metabolite profiles are associated with higher blood pressure. Despite the different pathophysiological mechanisms, both are characterized by changes in amino acids, most consistently valine and glycine, changes in fatty acids such as oleic acid and cholesteryl ester, changes in glucose metabolism metabolites, such as pyruvate, glucose and lactic acid, and changes in lipids such as glycerol and triacylglycerol.

No previous studies assessed the association of early-pregnancy metabolites with blood pressure development throughout pregnancy. We observed the most consistent associations of higher early-pregnancy serum concentrations of PC.aa.C38.3, PC.aa.C40.4, Lyso.PC.a.C14.0, Lyso.PC.a.C16.1, Lyso.PC.a.20.3 and NEFA.C24.5 with higher blood pressure development throughout pregnancy. Previous studies have reported associations of PC.aa, Lyso.PC.a and NEFA metabolites with cardiovascular risk factors and cardiovascular disease in adults [45,46]. Phospholipids are involved in transport of fatty acids, cell differentiation, inflammatory signaling and regulation of cell apoptosis. Higher phosphatidylcholine levels increase expression of several genes that activate lipogenesis, resulting in higher steady state plasma lipid levels [47,48]. Higher plasma PC.aa C38.3 has been associated with higher BMI, hip circumference and waist circumference in both young and middle-aged adults, and with type 2 diabetes [49,50,51]. Furthermore, PC.aa C38.3 and C40.4 have been associated with myocardial infarctions [47,48,52]. In our study, Lyso.PC.a C14.0 was remarkable for associations with both systolic and diastolic blood pressure throughout pregnancy with relatively large effect estimates. Interestingly, in previous studies Lyso.PC.a C14.0 was the only predictor in infancy of overweight and obesity at early school age, and the only serum marker in breastfed infants responding to human milk protein content [53,54]. In young adults, Lyso.PC.a C14.0 was increased in subjects with high insulin resistance [50]. This metabolite might play a key role in human cardio metabolic health, and is of high interest in further research. Higher plasma concentration of NEFA metabolites, which are free fatty acids, have previously been associated with insulin resistance, obesity and higher blood pressure [45,46]. The association of the phospholipid and NEFA metabolites with cardiovascular risk factors, which are also risk factors for gestational hypertensive disorders, might explain their involvement in higher blood pressure development in pregnancy. In our study, higher early-pregnancy serum concentrations of several AA, SM and Carn.a metabolites were associated with higher blood pressure in at least one trimester. SM metabolites are closely related to phospholipids, mainly localize in the lipid micro domains on cellular membranes, and are involved in proliferation, migration, inflammation, and cell survival [55,56]. In adults, higher plasma SM levels have been associated with cardiovascular disease [46,57]. Carn.a metabolites are derivatives of long chain fatty acids required for import of fatty acids into mitochondria for beta-oxidation to occur. Because Carn.a metabolites are usually broken down within mitochondria, mitochondrial dysfunction causes incomplete oxidation of fatty acids and may result in accumulation of Carn.a [58]. Furthermore, higher serum Carn.a concentrations are associated with induced pro-inflammatory pathways [59]. Thus, Carn.a and SM might both play a role in the pathophysiology of gestational hypertensive disorder through disturbance of lipid metabolism and inflammation. Likewise, AA metabolites are involved in multiple cellular mechanisms, amongst others energy processes (Krebs cycle), anti-oxidative responses and immunity [60]. Alterations in early-pregnancy serum AA concentrations were also present in previous studies investigating women with early- and late-onset preeclampsia [9,11]. In our study, particularly AA ratios characteristic for reduced replenishing of metabolites in the Krebs cycle were associated with higher gestational blood pressure.

Our population-based study enabled the identification of changes in maternal early-pregnancy metabolome associated with higher blood pressure in pregnancy in the general population. Our results show that alterations in serum metabolites involved in lipid metabolism, energy processes, oxidation, and inflammatory processes are associated with gestational blood pressure changes, even within the physiological ranges. Our results should be considered hypothesis generating and are important from an etiological perspective. They provide novel insight into underlying biological mechanisms, potential future markers and intervention targets for gestational hypertensive disorders. Future large studies among multi-ethnic populations are needed, to confirm the associations of the metabolites with higher blood pressure in pregnancy. Hereafter, the identified metabolites should be taken forward to further investigate their diagnostic and prognostic value for gestational hypertensive disorders, in addition to common clinical characteristics. 

### 4.2. Methodological Considerations

This study benefitted from the prospective data collection from early pregnancy onwards, allowing repeated blood pressure measurements throughout the entire pregnancy. The low prevalence of gestational hypertensive disorders in our study sample might have reduced the ability to detect associations. Furthermore, due to the low prevalence in preeclampsia, we could not distinguish between early- and late-onset. Therefore, further studies including a larger number of women with gestational hypertensive disorders are needed. The metabolomics analyses were only available for a subsample of the Generation R cohort, which consisted of Dutch, relatively high educated and healthy participants. This might have affected the generalizability of our findings. We used a targeted metabolomics approach, allowing us to optimize the quantification of the metabolites of interest. However, relevant biological pathways might have been missed. Although we adjusted for many potential confounders, residual confounding cannot be excluded due to the observational nature of the study. Maternal serum metabolites concentrations in our study were all measured in early pregnancy, at a mean gestational age of 13 weeks. We had no information on metabolic profiles of mothers in the preconception period available. Therefore, in our study, we were unable to distinguish which metabolic alterations associated with higher blood pressure in pregnancy were specifically pregnancy-related and which were not. However, metabolic adaptations, such as changes in insulin resistance and glucose levels, already originate in first trimester of pregnancy, and so might gestational hypertensive disorders [61]. By excluding women with preexisting hypertension from our analysis, which showed similar results to the main analysis, we aimed to exclude pre-pregnancy pathology from our associations. Further studies should investigate changes in metabolite profiles from preconception until late-pregnancy related to blood pressure in pregnancy and gestational hypertensive disorders. This way, using repeated measurements of metabolite profiles, critical periods for the development of gestational hypertensive disorder can be determined.

## 5. Conclusions

Altered early pregnancy serum metabolite profiles mainly characterized by higher concentrations of metabolites from the PC.aa, Lyso.PC.a and NEFA groups, and to a lesser extent the AA and Carn.a groups, are associated with higher gestational blood pressure development within the physiological ranges. Those metabolite groups are involved in lipid metabolism, energy processes, oxidation, and inflammatory processes. Our findings are important from an etiological perspective and, after further replication in large multi-ethnic populations including more women with gestational hypertension and preeclampsia, might improve the early identification of women at increased risk of gestational hypertensive disorders.

## Figures and Tables

**Table 1:** General characteristics of the study population.

Characteristic	Total Sample *n* = 803
Age at enrolment, mean (±SD), years	31.4 (4.1)
Parity, *n* (%)	
Nulliparous	490 (61.0)
Multiparous	313 (39.0)
Ethnicity, *n* (%)	
Dutch	803 (100)
Other	0 (0)
Education, *n* (%)	
Primary	15 (1.9)
Secondary	278 (34.8)
Higher	506 (63.3)
Pre-pregnancy body mass index, median (95% range), kg/m^2^	22.6 (20.9, 25.2)
Smoking, *n* (%)	
Never smoked during pregnancy	553 (75.9)
Smoked until pregnancy was known	73 (10.0)
Continued smoking in pregnancy	103 (14.1)
Systolic blood pressure, mean (SD), mmHg	
Early pregnancy	118.9 (13.0)
Mid pregnancy	119.4 (12.3)
Late pregnancy	120.4 (11.0)
Diastolic blood pressure, mean (SD), mmHg	
Early pregnancy	67.0 (10.0)
Mid pregnancy	68.1 (9.7)
Late pregnancy	69.9 (9.3)
Gestational hypertensive disorders, *n* (%)	
Gestational hypertension	37 (4.8)
Preeclampsia	12 (1.6)
History of hypertensive disorders, *n*(%)	
Pre-existing hypertension	9 (1.1)
Gestational hypertensive disorders	38 (4.7)

Values represent mean (±SD), median (95% range) or number of participants (valid %). Number of missing values per covariate: education *n* = 4 (0.5%), pre-pregnancy body mass index *n* = 120 (14.9%), smoking *n* = 86 (10.7%).

**Table 2:** Selected models for the prediction of systolic and diastolic blood pressure.

Model	Difference in Blood Pressure (95% CI)	Adjusted R^2^ (%)	SD Residuals
Clinical model systolic blood pressure		2.6	11.9
Maternal age	0.22 (−0.02, 0.46)		
Pre-pregnancy BMI	−0.52 (−0.76, −0.27)		
Parity	−0.41 (−1.80, 0.98)		
Smoking	−0.39 (−3.09, 2.31)		
Metabolite model systolic blood pressure		9.2	11.3
Maternal age	0.24 (−1.76, 0.48)		
Pre-pregnancy BMI	−0.54 (−7.92, −0.28)		
Parity	−0.62 (−1.99, 0.76)		
Smoking	0.19 (−2.47, 2.84)		
Arginine	−0.03 (−0.25, 0.02)		
Asparagine	0.03 (−0.07, 0.13)		
Glycine	0.01 (−0.01, 0.03)		
Lysine	0.06 (0.03, 0.09)		
Tryptophan	−0.14 (−0.23, −0.05)		
NEFA_18_0	−0.02 (−0.13, 0.09)		
NEFA_26_0	−7.80 (−17.80, 2.20)		
PC.aa.C34.4	−1.17 (−2.42, 0.08)		
PC.ae.C36.5	−0.18 (−0.57, 0.21)		
SM.a.C37.1	1.73 (−0.16, 3.62)		
SM.a.C38.2	0.14 (−0.08, 0.37)		
SM.a.C39.2	3.31 (0.53, 6.08)		
SM.a.C41.1	−0.26 (−0.64, 0.11)		
SM.a.C43.2	−1.12 (−2.58, 0.33)		
Carn.a.C16.1	29.34 (−12.25, 70.96)		
Carn.a.C16.2	26.95 (−59.61, 113.52)		
Asn/asp	0.27 (−1.14, 1.68)		
Clinical model diastolic blood pressure		0.5	8.7
Maternal age	0.12 (−0.06, 0.29)		
Pre-pregnancy BMI	−0.08 (−0.26, 0.09)		
Parity	−1.05 (−2.08, −0.03)		
Smoking	1.78 (−0.21, 3.76)		
Metabolite model diastolic blood pressure		7.5	8.3
Maternal age	0.11 (−0.07, 0.28)		
Pre-pregnancy BMI	−0.06 (−0.25, 1.26)		
Parity	−1.18 (−2.10, −0.17)		
Smoking	2.20 (0.25, 4.15)		
Arginine	−0.04 (−0.09, 0.00)		
Asparagine	0.04 (−0.04, 0.11)		
Glycine	0.01 (−0.01, 0.03)		
Lysine	0.04 (0.02, 0.06)		
Tryptophan	−0.06 (−0.13, 0.00)		
NEFA_18_0	−0.03 (−0.12, 0.05)		
NEFA_26_0	−3.93 (−11.3, 3.42)		
PC.aa.C34.4	−1.04 (−1.96, −0.12)		
PC.ae.C36.5	−0.31 (−0.59, −0.02)		
SM.a.C37.1	1.53 (0.14, 2.92)		
SM.a.C38.2	0.14 (−0.02, 0.31)		
SM.a.C39.2	1.27 (−0.77, 3.31)		
SM.a.C41.1	0.13 (−0.14, 0.40)		
SM.a.C43.2	−1.20 (−2.27, −0.13)		
Carn.a.C16.1	8.24 (−22.35, 38.83)		
Carn.a.C16.2	43.3 (−20.39, 106.91)		
Asn/asp	0.24 (−0.79, 1.28)		

Values represent regression coefficients (95% confidence interval (CI)), adjusted *R*^2^ obtained from linear regression models and standard deviation (SD) of the residuals.

## Data Availability

The data presented in this study are available on request from the corresponding author. The data are not publicly available due to privacy and ethical restrictions.

## Supplementary Materials

The following supporting information can be downloaded at: https://www.mdpi.com/article/10.3390/metabo12121169/s1, Table S1: Parameters for mass-spectrometry detection and identifications, Table S2: Maternal early-pregnancy serum metabolite concentrations, Table S3: Associations of maternal early-pregnancy individual metabolites with systolic and diastolic blood pressure in early-, mid- and late pregnancy. Basic model, Table S4: Associations of early-pregnancy individual metabolites with systolic blood pressure in early-, mid- and late pregnancy. Full model, Table S5: Associations of early-pregnancy individual metabolites with diastolic blood pressure in early-, mid- and late pregnancy. Full model, Table S6: Associations of early-pregnancy individual metabolites with systolic blood pressure in early-, mid- and late pregnancy. Full model, additional adjustment for family history of hypertensive disorders, Table S7: Associations of early-pregnancy individual metabolites with diastolic blood pressure in early-, mid- and late pregnancy. Full model, additional adjustment for family history of hypertensive disorders, Table S8: Associations of early-pregnancy individual metabolites with systolic blood pressure in early-, mid- and late pregnancy. Full model, sensitivity analysis excluding women without preexisting hypertension, Table S9: Associations of early-pregnancy individual metabolites with diastolic blood pressure in early-, mid- and late pregnancy. Full model, sensitivity analysis excluding women without preexisting hypertension, Table S10: Lasso regression on systolic blood pressure change from first to third trimester, Table S11: Lasso on diastolic blood pressure change from first to third trimester, Figure S1: Flowchart of participants included in the study, Figure S2: Direct Acyclic Graph, Figure S3: ROC curves on the prediction of gestational hypertensive disorders.

## Abbreviations

AA	amino-acids
Carn	carnitines
Carn.a	acyl-carnitines
NEFA	non-esterified fatty acids
Lyso.PC.a	acyl-lysophosphatidylcholines
Lyso.PC.e	alkyl-lysophosphatidylcholines
PC	phosphatidylcholines
PC.aa	diacyl-phosphatidylcholines
PC.ae	acyl-alkyl-phosphatidylcholines
PL	phospholipids
SM	sphingomyelins
