# Supplementary material for: Associations of Early Pregnancy Metabolite Profiles with Gestational Blood Pressure Development
Source: Metabolites. 2022 Nov 24;12(12):1169. doi: 10.3390/metabo12121169 (PMC9785484; doi:10.3390/metabo12121169)
Supplement: Supplementary file 1 [file metabolites-12-01169-s001.zip › metabolites-1990417-supplementary.pdf]

Supplementary Material

Supplemental Text. Methods for blood metabolomics analysis.

At enrollment in the study, maternal non-fasting blood samples were obtained at a mean gestational age of 13.0 weeks (standard deviation ± 1.7 weeks) by research nurses.[1] Blood samples were transported to the regional laboratory (STAR-MDC), spun and stored at -80 ºC within 4h after collection. They were transported on dry ice to the Division of Metabolic and Nutritional Medicine of the Dr. von Hauner Children's Hospital in Munich, Germany.

As described in detail previously, a targeted metabolomics approach was used to determine the serum concentrations (µmol/L) of AA, non-esterified fatty acids (NEFA), phospholipids (PL) (including diacyl-phosphatidylcholines (PC.aa), acyl-alkyl-phosphatidylcholines (PC.ae) , acyl-lysophosphatidylcholines (Lyso.PC.a), alkyl-lysophosphatidylcholines (Lyso.PC.e), sphingomyelines (SM)) and carnitines (Carn) (including free carnitine (Free Carn) and acyl-carnitines (Carn.a)).[1,2] Proteins of 50 µL serum were precipitated by adding 450 µL methanol with the following internal standards: labeled amino acid standards set A (NSK-A-1, Cambridge Isotope Laboratories (CIL), USA), 15N2-L-asparagine (NLM-3286-0.25, CIL, USA), indole-D5-L-tryptophan (DLM-1092-0.5, CIL, USA), U-13C16-palmitic acid (CLM-409-MPT-PK, CIL, USA), D3-acetyl-carnitine (DLM-754-PK, CIL, USA), D3-octanoyl-carnitine (DLM-755-0.01, CIL, USA), and D3-palmitoyl-carnitine (DLM-1263-0.01, CIL, USA), tridecanoyl-2-hydroxy-sn-glycero-3-phosphocholine (855476, Avanti Polar Lipids, USA) and 1,2-dimyristoyl-sn-glycero-3-phospocholine (850345, Avanti Polar Lipids, USA).[1] If sample volume was less than optimal, the concentrations were corrected by the respective factor. Sample volumes less than 25 µL were considered missing. After centrifugation, we split the supernatant into aliquots. We analyzed AA by liquid chromatography tandem mass spectrometry (LC-MS/MS), as described previously . An aliquot of the supernatant was used for the derivatization to AA butylester with hydrocholic acid in 1-buthanol. After evaporation, the residues were dissolved in water/methanol (80:20; (v/v)) with 0.1% formic acid.[1] The samples were analyzed with 1100 high-performance liquid chromatography (HPLC) system (Agilent, Waldbronn, Germany) equipped with 150 x 2.1 mm, 3.5 µm particle size C18 HPLC column (X-Bridge, Waters, Milford, USA) and 0.1% heptafluorobutyric acid as an ion pair reagent in the mobile phases A (water) and B (methanol). We performed mass spectrometry (MS) detection with an API2000 tandem mass spectrometer (MS/MS) (AB Sciex, Darmstadt, Germany). IUPAC-IUB Nomenclature was used for notation of AA. For AA, information on the identification and analysis for each metabolite and class are presented in the table below.

NEFA, PL and Carn were measured with a 1200 SL HPLC system (Agilent, Waldbronn, Germany) coupled to a 4000 QTRAP tandem mass spectrometer (AB Sciex, Darmstadt, Germany).[3,4] NEFA were analyzed by injection of the supernatant to a LC-MS/MS operating in negative electrospray ionization (ESI) mode where they separated by gradient elution on a 100 x 3.0 mm, 1.9 µm particle size Purusuit UPS Diphenyl column from Varian (Darmstadt, Germany) using 5 mM ammonium acetate in water as mobile phase A and acetonitrile /isopropanol (80:20, (v/v)) as mobile phase B.[1] NEFA species were quantified using GLC-85 reference standard mixture (Nu-Chek Prep, USA). For NEFA, information on the identification and analysis for each metabolite and class are presented in the table below.

PL were analyzed by flow-injection-analysis with LC-MS/MS coupled with ESI . The system was run in positive ionization mode with 5% water in isopropanol as mobile phase A and 5% water in methanol as mobile phase B. The method included 2 periods of 2.6 minutes each. The total runtime for both periods was 5.2 minutes and 0.8 injection time with a total injection volume of 60 µL. The analysis was performed for PC.aa, PC.ae, Lyso.PC.a, Lyso.PC.e and SM. For Carn (Free Carn and Carn.a) analysis we performed flow-injection analysis of the supernatant into a LC-MS/MS system using an isocratic elution with 76% isopropanol, 19% methanol and 5% water.[1] The mass spectrometer was equipped with electrospray ionization and operated in the positive ionization mode. PL and Carn.a were quantified using aliquots of a commercially available lyophilized control plasma (ClinChek®, Recipe, Germany), where the concentrations have been determined by AbsoluteIDQ p150 Kit from Biocrates®, a previous published LC-MS/MS method and by in-house quantification with various standards . Information on the identification and analysis of PL and Carn.a are given in the table below.[1] The entire analytical process was controlled and post-processed by Analyst 1.6.1. and R Software.[2] The analytical technique used can determine the total number of total bonds, but not the position of the double bonds and the distribution of the carbon atoms between FA side chains. The following notation was used for NEFA, PL and

Carn.a: X:Y, where X denotes the length of the carbon chain, and Y the number of double bonds. The ‘a’ denotes an acyl chain bound to the backbone of an ester bond (‘acyl-’) and the ‘e’ represents an ether bond (‘alkyl-’).

Data quality control (QC) was based on thresholds of 25% and 35% for the intra- and inter-batch coefficients of variation respectively.[1] To correct for batch effects, metabolite concentrations were divided by the ratio of the intra-batch and inter-batch median of the QC samples. Metabolites and participants with more than 50% of missing values were excluded. Missing metabolite values of the remaining metabolites and participants were imputed using the Random Forest algorithm (R package missForest), which is among the best performing imputation methods for mass-spectrometry based metabolomics data with missing values at random or missing values completely at random. The Random Forest algorithm works by aggregating the predictions made by multiple decision trees of varying depth. The trees (or models) are relatively uncorrelated, as each tree samples at random from the dataset and the trees use different features to make the decision instead of always picking the feature that provides the most separation.

For analyses, we categorized metabolites into general metabolite groups based on chemical structure (AA, NEFA, PC.aa, PC.ae, Lyso.PC.a, Lyso.PC.e, SM, Free Carn and Carn.a) and in detailed metabolite subgroups based on chemical structure and biological relevance (AA: branched chain AA (BCAA), aromatic AA (AAA), essential AA, non-essential AA; NEFA, PC.aa, PC.ae, Lyso.PC.a, Lyso.PC.e and SM: saturated, mono-unsaturated, poly-unsaturated; Carn.a: short-chain, medium-chain, long-chain).[1] Correlations between metabolites were assessed in a previous study, concluding high correlations between individual metabolites within groups of metabolites with similar chemical structures, but lower correlations between groups of metabolites with different chemical structures (Voerman et al. 2020). To correct for right skewedness, individual metabolite concentrations were square root transformed. To facilitate interpretation of the effect estimates, standard deviation scores (SDS) were calculated for both metabolite groups and individual metabolites.

Table S1: Parameters for mass-spectrometry detection and identifications

Parameters for mass-spectrometry detection and identification for amino-acids and non-esterified fatty acids, including the labelled internal standards .								
**ID***	**Rt** minutes	**Q1**	**Q3**	**DP**	**CE**	**CXP**	**Adduct**	**MSI ID Level**
**Amino acids**								
Ala1	7,6	146,182	44	11	25	4	(M+H)+	1
Ala2	7,6	146,182	90	11	13	12	(M+H)+	1
AlaIS	7,6	150,168	48,1	31	25	6	(M+H)+	1
Arg1	7,3	231,201	70,1	21	39	8	(M+H)+	1
Arg2	7,3	231,276	60	21	33	8	(M+H)+	1
Arg3	7,3	231,276	172,2	21	21	8	(M+H)+	1
ArgIS	7,3	236,201	75,1	21	39	8	(M+H)+	1
Asn1	5,4	189,303	144,1	21	17	6	(M+H)+	1
Asn2	5,4	189,303	74	21	27	8	(M+H)+	1
Asn3	5,4	189,303	130,3	21	19	4	(M+H)+	1
AsnIS	5,4	191,116	145,2	21	19	6	(M+H)+	1
Asp1	13,8	246,262	144,3	21	19	6	(M+H)+	1
Asp2	13,8	246,262	88,1	21	27	2	(M+H)+	1
Asp3	13,8	246,262	74,2	21	35	8	(M+H)+	1
AspIS	13,8	249,278	147,3	16	19	6	(M+H)+	1
Cit1	6,3	232,249	70,1	16	43	8	(M+H)+	1
Cit2	6,3	232,249	215,3	16	17	10	(M+H)+	1
Cit3	6,3	232,249	113,2	16	27	4	(M+H)+	1
CitIS	6,3	234,237	115,2	16	27	4	(M+H)+	1

Cys1	11,8	353,133	129,9	21	29	14	(M+H)+	1
Cys2	11,8	353,078	73,9	21	47	8	(M+H)+	1
Cys3	11,8	353,078	208,1	21	21	8	(M+H)+	1
CysIS	11,8	357,133	129,9	21	29	14	(M+H)+	1
Gln1	5,6	203,1	84,1	11	30	11	(M+H)+	1
Gln2	5,6	203,1	186,1	11	16	8	(M+H)+	1
Gln3	5,6	203,1	130,1	11	21	15	(M+H)+	1
GlnIS	5,6	208,1	89,1	11	30	11	(M+H)+	1
Glu1	14,3	260,312	84	16	37	10	(M+H)+	1
Glu2	14,3	260,312	186,2	16	19	8	(M+H)+	1
Glu3	14,3	260,312	130,1	16	25	4	(M+H)+	1
GluIS	14,3	263,297	87,1	16	35	10	(M+H)+	1
Gly1	6,2	132,19	76	16	13	8	(M+H)+	1
Gly2	6,2	132,19	57	16	19	6	(M+H)+	1
GlyIS	6,2	134,17	77,9	16	13	10	(M+H)+	1
His1	6,8	212,18	109,9	16	27	14	(M+H)+	1
His2	6,8	212,271	83,1	21	41	10	(M+H)+	1
His3	6,8	212,271	93	21	41	8	(M+H)+	1
HisIS	6,8	215,184	112,9	16	27	14	(M+H)+	1
Ile1	12,4	188,327	86	21	21	10	(M+H)+	1
Ile2	12,4	188,327	69	21	33	8	(M+H)+	1
Ile3	12,4	188,327	44,1	21	43	4	(M+H)+	1
Leu1	12,6	188,2	86	21	21	10	(M+H)+	1
Leu2	12,6	188,2	69	21	33	8	(M+H)+	1
Leu3	12,6	188,2	44,1	21	43	4	(M+H)+	1
LeuIS	12,6	191,338	89,2	11	19	2	(M+H)+	1
Lys1	7,2	203,2	84,1	21	33	2	(M+H)+	1
Lys2	7,2	203,2	186,2	21	17	8	(M+H)+	1
Lys3	7,2	203,2	56	21	61	6	(M+H)+	1
LysIS	7,2	207,2	88,1	21	33	2	(M+H)+	1
Met1	10,9	206,245	104,1	31	19	4	(M+H)+	1
Met2	10,9	206,245	61,1	31	41	6	(M+H)+	1
Met3	10,9	206,245	56	31	31	6	(M+H)+	1
MetIS	10,9	209,2	107,1	11	30	5	(M+H)+	1
Orn1	6,7	189,304	70,1	16	29	8	(M+H)+	1
Orn2	6,7	189,304	172,2	16	15	8	(M+H)+	1
Orn3	6,7	189,304	116,1	16	21	6	(M+H)+	1
OrnIS	6,7	191,338	174,1	11	15	8	(M+H)+	1
Phe1	12,8	222,248	120,3	21	23	4	(M+H)+	1
Phe2	12,8	222,248	103,1	21	49	10	(M+H)+	1
Phe3	12,8	222,248	77	21	69	8	(M+H)+	1
PheIS	12,8	228,284	126,2	16	21	6	(M+H)+	1
Pro1	7,8	172,291	70,1	26	25	8	(M+H)+	1
Pro2	7,8	172,291	116,2	26	19	4	(M+H)+	1
Pro3	7,8	172,291	57,1	26	27	6	(M+H)+	1
ProIS	7,8	175,18	73	16	27	8	(M+H)+	1

Pro2IS	7,8	175,18	118,9	16	21	16	(M+H)+	1
Ser1	6	162,255	60	16	23	6	(M+H)+	1
Ser2	6	162,255	106,2	16	15	4	(M+H)+	1
Ser3	6	162,255	88,3	16	19	2	(M+H)+	1
SerIS	6	165,255	63	16	23	6	(M+H)+	1
Thr1	7,2	176,24	73,9	16	23	10	(M+H)+	1
Thr2	7,2	176,24	55,9	16	31	6	(M+H)+	1
Thr3	7,2	176,24	102,1	16	19	4	(M+H)+	1
ThrIS	7,2	180,24	75,9	16	23	10	(M+H)+	1
Trp1	13,3	261,284	244,2	21	17	10	(M+H)+	1
Trp2	13,3	261,284	159,3	21	25	6	(M+H)+	1
Trp3	13,3	261,284	132,4	21	41	6	(M+H)+	1
TrpIS	13,3	266,284	249,2	21	17	10	(M+H)+	1
Tyr1	10,24	238,241	136,1	21	23	6	(M+H)+	1
Tyr2	10,24	238,241	91,2	21	47	4	(M+H)+	1
Tyr3	10,24	238,241	119,2	21	37	4	(M+H)+	1
TyrIS	10,24	244,266	142,2	16	23	8	(M+H)+	1
Val1	10,8	174,213	72	16	19	8	(M+H)+	1
Val2	10,8	174,213	55	16	41	6	(M+H)+	1
Val3	10,8	174,213	118,2	16	15	6	(M+H)+	1
ValIS	10,8	182,275	80,2	16	21	10	(M+H)+	1
**Non-esterified fatty acids**								
4_0	0,7	87	87	-45	-8	-7	(M-H)-	1
5_0	0,8	101	101	-45	-8	-7	(M-H)-	1
6_0	1,0	115,1	115,1	-50	-8	-7	(M-H)-	1
7_0	1,2	129,1	129,1	-50	-8	-7	(M-H)-	1
8_0	1,6	143,1	143,1	-55	-8	-7	(M-H)-	1
9_0	2,0	157,1	157,1	-55	-8	-7	(M-H)-	1
10_0	2,4	171,146	171,146	-60	-8	-13	(M-H)-	1
11_0	2,7	185,162	185,162	-65	-8	-11	(M-H)-	1
12_0	3,1	199,178	199,178	-68	-8	-11	(M-H)-	1
12_1	2,6	197,162	197,162	-72	-8	-7	(M-H)-	1
13_0	3,5	213,193	213,193	-70	-8	-17	(M-H)-	1
13_1	2,9	211,178	211,178	-74	-8	-7	(M-H)-	1
14_0	3,9	227,209	227,209	-120	-25	-13	(M-H)-	1
14_1	3,3	225,193	225,193	-75	-8	-13	(M-H)-	1
14_2	2,7	223,178	223,178	-78	-8	-7	(M-H)-	1
15_0	4,2	241,225	241,225	-75	-8	-7	(M-H)-	1
15_1	3,7	239,209	239,209	-75	-10	-7	(M-H)-	1
16_0	4,6	255,24	255,24	-150	-35	-13	(M-H)-	1
16_1	4,0	253,225	253,225	-78	-30	-13	(M-H)-	1
16_2	3,5	251,209	251,209	-79	-8	-7	(M-H)-	1
16_3	2,9	249,193	249,193	-78	-8	-7	(M-H)-	1
16_4	2,4	247,178	247,178	-78	-8	-7	(M-H)-	1
17_0	5,0	269,256	269,256	-85	-12	-7	(M-H)-	1
17_1	4,4	267,24	267,24	-75	-10	-7	(M-H)-	1

17_2	3,9	265,225	265,225	-79	-8	-7	(M-H)-	1
18_0	5,4	283,272	283,272	-150	-35	-7	(M-H)-	1
18_1	4,8	281,256	281,256	-150	-37	-7	(M-H)-	1
18_2	4,2	279,24	279,24	-130	-32	-7	(M-H)-	1
18_3	3,7	277,225	277,225	-120	-10	-7	(M-H)-	1
18_4	3,1	275,209	275,209	-72	-8	-7	(M-H)-	1
19_0	5,7	297,287	297,287	-90	-8	-7	(M-H)-	1
19_1	5,2	295,272	295,272	-85	-8	-7	(M-H)-	1
19_2	4,6	293,256	293,256	-80	-8	-7	(M-H)-	1
20_0	6,1	311,303	311,303	-95	-8	-13	(M-H)-	1
20_1	5,5	309,287	309,287	-90	-8	-9	(M-H)-	1
20_2	5,0	307,272	307,272	-85	-8	-9	(M-H)-	1
20_3	4,4	305,256	305,256	-80	-8	-9	(M-H)-	1
20_4	3,9	303,24	303,24	-150	-10	-9	(M-H)-	1
20_5	3,3	301,225	301,225	-61	-8	-7	(M-H)-	1
22_0	6,8	339,334	339,334	-100	-14	-11	(M-H)-	1
22_1	6,3	337,318	337,318	-80	-8	-9	(M-H)-	1
22_2	5,7	335,303	335,303	-80	-8	-11	(M-H)-	1
22_3	5,2	333,287	333,287	-71	-8	-7	(M-H)-	1
22_4	4,6	331,272	331,272	-62	-8	-7	(M-H)-	1
22_5	4,0	329,256	329,256	-53	-8	-7	(M-H)-	1
22_6	3,5	327,24	327,24	-150	-6	-13	(M-H)-	1
24_0	7,6	367,365	367,365	-106	-8	-7	(M-H)-	1
24_1	7,0	365,35	365,35	-97	-8	-11	(M-H)-	1
24_2	6,5	363,334	363,334	-81	-8	-7	(M-H)-	1
24_3	5,9	361,318	361,318	-69	-8	-7	(M-H)-	1
24_4	5,4	359,303	359,303	-57	-8	-7	(M-H)-	1
24_5	4,8	357,287	357,287	-44	-8	-7	(M-H)-	1
24_6	4,2	355,272	355,272	-32	-8	-7	(M-H)-	1
26_0	8,3	395,397	395,397	-113	-8	-7	(M-H)-	1
26_1	7,8	393,381	393,381	-97	-8	-7	(M-H)-	1
26_2	7,2	391,365	391,365	-82	-8	-7	(M-H)-	1
26_3	6,7	389,35	389,35	-67	-8	-7	(M-H)-	1
26_4	6,1	387,334	387,334	-52	-8	-7	(M-H)-	1
26_5	5,5	385,318	385,318	-36	-8	-7	(M-H)-	1
26_6	5,0	383,303	383,303	-21	-8	-7	(M-H)-	1
16_0-IS	4,1	271	271	-80	-10	-7	(M-H)-	1
20_4-IS	3,7	311,24	311,24	-70	-10	-9	(M-H)-	1
22_6-IS	3,6	332,24	332,24	-40	-6	-13	(M-H)-	1
22_0-IS	5,9	342,33	342,33	-100	-14	-11	(M-H)-	1
10_0-IS	2,4	190,15	190,15	-60	-8	-13	(M-H)-	1
6_0-IS	0,9	126,1	126,1	-50	-8	-7	(M-H)-	1

ID metabolite identity, Rt retention time in minutes, Q1/Q3 quadrupole 1 and 3, IS internal standard, DP declustering potential, CE collison energy, CXP collision cell exit potential, MSI ID Metabolomics Standards Initiative identification.
*The numbers next to the ID refer to the different transitions used.

Parameters for mass-spectrometry detection and identifications for phospholipids and acyl-carnitines.							
**ID**	**Sofia.ID**	**Q1**	**Q3**	**CP1**	**CP2**	**Adduct**	**MSI ID Level**
Carn.C0	Carn	162,1	85,1	29,25	29,85	(M+H)+	1
Carn.C10	Carn.C10	316,2	85,1	0,1465	0,1395	(M+H)+	1
Carn.C10.1	Carn.C10.1	314,2	85,1	0,13	0,122	(M+H)+	1
Carn.C10.2	Carn.C10.2	312,2	85,1	0,0275	0,0275	(M+H)+	1
Carn.C12	Carn.C12	344,3	85,1	0,06975	0,0705	(M+H)+	1
Carn.C12.1	Carn.C12.1	342,3	85,1	0,13875	0,1245	(M+H)+	1
Carn.C12.DC	Carn.C12.DC	374,3	85,1	0,0475	0,0495	(M+H)+	1
Carn.C14	Carn.C14	372,3	85,1	0,03875	0,037	(M+H)+	1
Carn.C14.1	Carn.C14.1	370,3	85,1	0,07725	0,078	(M+H)+	1
Carn.C14.1.OH	Carn.C14.1.OH	386,3	85,1	0,0095	0,0095	(M+H)+	1
Carn.C14.2	Carn.C14.2	368,3	85,1	0,014	0,0135	(M+H)+	1
Carn.C14.2.OH	Carn.C14.2.OH	384,3	85,1	0,0075	0,007	(M+H)+	1
Carn.C16	Carn.C16	400,3	85,1	0,075	0,0775	(M+H)+	1
Carn.C16.1	Carn.C16.1	398,3	85,1	0,02625	0,027	(M+H)+	1
Carn.C16.1.OH	Carn.C16.1.OH	414,3	85,1	0,01	0,0095	(M+H)+	1
Carn.C16.2	Carn.C16.2	396,3	85,1	0,0045	0,005	(M+H)+	1
Carn.C16.2.OH	Carn.C16.2.OH	412,3	85,1	0,01	0,0095	(M+H)+	1
Carn.C16.OH	Carn.C16.OH	416,3	85,1	0,0055	0,0055	(M+H)+	1
Carn.C18	Carn.C18	428,4	85,1	0,03425	0,0355	(M+H)+	1
Carn.C18.1	Carn.C18.1	426,4	85,1	0,0915	0,0915	(M+H)+	1
Carn.C18.1.OH	Carn.C18.1.OH	442,4	85,1	0,0075	0,0075	(M+H)+	1
Carn.C18.2	Carn.C18.2	424,3	85,1	0,043	0,043	(M+H)+	1
Carn.C2	Carn.C2	204,1	85,1	4,48	4,52	(M+H)+	1
Carn.C3	Carn.C3	218,1	85,1	0,345	0,355	(M+H)+	1
Carn.C3.1	Carn.C3.1	216,1	85,1	0,0055	0,005	(M+H)+	1
Carn.C3.DC.C4.OH.	Carn.C3.DC	248,1	85,1	0,03875	0,0495	(M+H)+	1
Carn.C3.OH	Carn.C3.OH	234,1	85,1	0,02125	0,022	(M+H)+	1
Carn.C4	Carn.C4	232,2	85,1	0,179	0,1875	(M+H)+	1
Carn.C4.1	Carn.C4.1	230,1	85,1	0,019	0,019	(M+H)+	1
Carn.C5	Carn.C5	246,2	85,1	0,10375	0,103	(M+H)+	1
Carn.C5.1	Carn.C5.1	244,2	85,1	0,0195	0,02	(M+H)+	1
Carn.C5.1.DC	Carn.C5.1.DC	274,1	85,1	0,0175	0,015	(M+H)+	1
Carn.C5.DC.C6.OH.	Carn.C5.DC	276,1	85,1	0,01925	0,0205	(M+H)+	1
Carn.C5.M.DC	Carn.C5.M.DC	290,2	85,1	0,03225	0,031	(M+H)+	1
Carn.C5.OH.C3.DC.M.	Carn.C5.OH	262,2	85,1	0,0575	0,0615	(M+H)+	1
Carn.C6.1	Carn.C6.1	258,2	85,1	0,0125	0,0125	(M+H)+	1
Carn.C6.C4.1.DC.	Carn.C6	260,2	85,1	0,04475	0,046	(M+H)+	1
Carn.C7.DC	Carn.C7.DC	304,2	85,1	0,02575	0,027	(M+H)+	1
Carn.C8	Carn.C8	288,2	85,1	0,1105	0,1195	(M+H)+	1
Carn.C8.1	Carn.C8.1	286,2	85,1	0,07525	0,0745	(M+H)+	1
Carn.C9	Carn.C9	302,2	85,1	0,03425	0,034	(M+H)+	1
lysoPCaC14.0	lyso.PC.a.C14.0	468,3	184	2,575	2,615	(M+H)+	1
lysoPCaC16.0	lyso.PC.a.C16.0	496,3	184	98,775	95,6	(M+H)+	1
lysoPCaC16.1	lyso.PC.a.C16.1	494,3	184	3	2,96	(M+H)+	1

lysoPCaC17.0	lyso.PC.a.C17.0	510,4	184	1,7325	1,66	(M+H)+	1
lysoPCaC18.0	lyso.PC.a.C18.0	524,4	184	26,675	26,2	(M+H)+	1
lysoPCaC18.1	lyso.PC.a.C18.1	522,4	184	18,275	18,05	(M+H)+	1
lysoPCaC18.2	lyso.PC.a.C18.2	520,3	184	32,6	32,15	(M+H)+	1
lysoPCaC20.3	lyso.PC.a.C20.3	546,4	184	1,96	2,03	(M+H)+	1
lysoPCaC20.4	lyso.PC.a.C20.4	544,3	184	6,5375	6,48	(M+H)+	1
lysoPCaC24.0	lyso.PC.a.C24.0	608,5	184	0,76625	0,8805	(M+H)+	1
lysoPCaC26.0	lyso.PC.a.C26.0	636,5	184	1,815	2,22	(M+H)+	1
lysoPCaC26.1	lyso.PC.a.C26.1	634,5	184	3,4275	3,71	(M+H)+	1
lysoPCaC28.0	lyso.PC.a.C28.0	664,5	184	1,3125	1,535	(M+H)+	1
lysoPCaC28.1	lyso.PC.a.C28.1	662,5	184	1,52	1,785	(M+H)+	1
lysoPCaC6.0	lyso.PC.a.C6.0	356,2	184	0,05025	0,0455	(M+H)+	1
PCaaC24.0	PC.aa.C24.0	622,4	184	0,4935	0,596	(M+H)+	1
PCaaC26.0	PC.aa.C26.0	650,5	184	2,735	3,33	(M+H)+	1
PCaaC28.1	PC.aa.C28.1	676,5	184	2,6525	2,775	(M+H)+	1
PCaaC30.0	PC.aa.C30.0	706,5	184	3,49	3,535	(M+H)+	1
PCaaC30.2	PC.aa.C30.2	702,5	184	0,51625	0,548	(M+H)+	1
PCaaC32.0	PC.aa.C32.0	734,6	184	11	11,05	(M+H)+	1
PCaaC32.1	PC.aa.C32.1	732,6	184	13,3	13,75	(M+H)+	1
PCaaC32.2	PC.aa.C32.2	730,5	184	2,8125	2,87	(M+H)+	1
PCaaC32.3	PC.aa.C32.3	728,5	184	0,5415	0,525	(M+H)+	1
PCaaC34.1	PC.aa.C34.1	760,6	184	167,5	167,5	(M+H)+	1
PCaaC34.2	PC.aa.C34.2	758,6	184	278,75	282	(M+H)+	1
PCaaC34.3	PC.aa.C34.3	756,6	184	14,65	13,6	(M+H)+	1
PCaaC34.4	PC.aa.C34.4	754,5	184	1,675	1,65	(M+H)+	1
PCaaC36.0	PC.aa.C36.0	790,6	184	2,305	2,095	(M+H)+	1
PCaaC36.1	PC.aa.C36.1	788,6	184	35,4	34,15	(M+H)+	1
PCaaC36.2	PC.aa.C36.2	786,6	184	170,5	173	(M+H)+	1
PCaaC36.3	PC.aa.C36.3	784,6	184	97,8	98,9	(M+H)+	1
PCaaC36.4	PC.aa.C36.4	782,6	184	125	127	(M+H)+	1
PCaaC36.5	PC.aa.C36.5	780,6	184	15,975	15,75	(M+H)+	1
PCaaC36.6	PC.aa.C36.6	778,5	184	0,9525	0,848	(M+H)+	1
PCaaC38.0	PC.aa.C38.0	818,7	184	1,8325	1,81	(M+H)+	1
PCaaC38.1	PC.aa.C38.1	816,6	184	1,17	0,9875	(M+H)+	1
PCaaC38.3	PC.aa.C38.3	812,6	184	29,275	28,55	(M+H)+	1
PCaaC38.4	PC.aa.C38.4	810,6	184	69,225	72,3	(M+H)+	1
PCaaC38.5	PC.aa.C38.5	808,6	184	34,775	35,8	(M+H)+	1
PCaaC38.6	PC.aa.C38.6	806,6	184	47,4	49,6	(M+H)+	1
PCaaC40.1	PC.aa.C40.1	844,7	184	0,457	0,425	(M+H)+	1
PCaaC40.2	PC.aa.C40.2	842,7	184	0,51675	0,436	(M+H)+	1
PCaaC40.3	PC.aa.C40.3	840,6	184	0,72825	0,646	(M+H)+	1
PCaaC40.4	PC.aa.C40.4	838,6	184	2,4325	2,51	(M+H)+	1
PCaaC40.5	PC.aa.C40.5	836,6	184	6,835	7,19	(M+H)+	1
PCaaC40.6	PC.aa.C40.6	834,6	184	15,6	16,6	(M+H)+	1
PCaaC42.0	PC.aa.C42.0	874,7	184	0,4535	0,4445	(M+H)+	1
PCaaC42.1	PC.aa.C42.1	872,7	184	0,2585	0,245	(M+H)+	1

PCaaC42.2	PC.aa.C42.2	870,7	184	0,2725	0,233	(M+H)+	1
PCaaC42.4	PC.aa.C42.4	866,7	184	0,248	0,222	(M+H)+	1
PCaaC42.5	PC.aa.C42.5	864,6	184	0,3165	0,3175	(M+H)+	1
PCaaC42.6	PC.aa.C42.6	862,6	184	0,55475	0,58	(M+H)+	1
PCaeC30.0	PC.ae.C30.0	692,6	184	0,432	0,4415	(M+H)+	1
PCaeC30.1	PC.ae.C30.1	690,5	184	0,691	0,882	(M+H)+	1
PCaeC30.2	PC.ae.C30.2	688,5	184	0,249	0,2505	(M+H)+	1
PCaeC32.1	PC.ae.C32.1	718,6	184	2,2625	2,28	(M+H)+	1
PCaeC32.2	PC.ae.C32.2	716,6	184	0,798	0,8295	(M+H)+	1
PCaeC34.0	PC.ae.C34.0	748,6	184	1,3025	1,275	(M+H)+	1
PCaeC34.1	PC.ae.C34.1	746,6	184	7,3125	7,285	(M+H)+	1
PCaeC34.2	PC.ae.C34.2	744,6	184	8,33	8,28	(M+H)+	1
PCaeC34.3	PC.ae.C34.3	742,6	184	5,845	6,035	(M+H)+	1
PCaeC36.0	PC.ae.C36.0	776,7	184	0,765	0,7355	(M+H)+	1
PCaeC36.1	PC.ae.C36.1	774,6	184	7,6675	6,57	(M+H)+	1
PCaeC36.2	PC.ae.C36.2	772,6	184	11,525	10,75	(M+H)+	1
PCaeC36.3	PC.ae.C36.3	770,6	184	5,5775	5,515	(M+H)+	1
PCaeC36.4	PC.ae.C36.4	768,6	184	11,05	11,15	(M+H)+	1
PCaeC36.5	PC.ae.C36.5	766,6	184	7,2275	7,405	(M+H)+	1
PCaeC38.0	PC.ae.C38.0	804,7	184	2,195	2,045	(M+H)+	1
PCaeC38.1	PC.ae.C38.1	802,7	184	1,8875	1,41	(M+H)+	1
PCaeC38.2	PC.ae.C38.2	800,7	184	3,125	2,535	(M+H)+	1
PCaeC38.3	PC.ae.C38.3	798,6	184	5,1625	4,68	(M+H)+	1
PCaeC38.4	PC.ae.C38.4	796,6	184	9,3975	9,28	(M+H)+	1
PCaeC38.5	PC.ae.C38.5	794,6	184	10,525	10,55	(M+H)+	1
PCaeC38.6	PC.ae.C38.6	792,6	184	4,71	4,805	(M+H)+	1
PCaeC40.0	PC.ae.C40.0	832,7	184	7,225	7,365	(M+H)+	1
PCaeC40.1	PC.ae.C40.1	830,7	184	1,555	1,55	(M+H)+	1
PCaeC40.2	PC.ae.C40.2	828,7	184	1,7025	1,6	(M+H)+	1
PCaeC40.3	PC.ae.C40.3	826,7	184	1,65	1,45	(M+H)+	1
PCaeC40.4	PC.ae.C40.4	824,7	184	2,0575	1,955	(M+H)+	1
PCaeC40.5	PC.ae.C40.5	822,6	184	3,285	3,24	(M+H)+	1
PCaeC40.6	PC.ae.C40.6	820,6	184	2,885	2,93	(M+H)+	1
PCaeC42.0	PC.ae.C42.0	860,7	184	0,52825	0,533	(M+H)+	1
PCaeC42.1	PC.ae.C42.1	858,7	184	0,53275	0,5395	(M+H)+	1
PCaeC42.2	PC.ae.C42.2	856,7	184	0,54675	0,5235	(M+H)+	1
PCaeC42.3	PC.ae.C42.3	854,7	184	1,065	0,9655	(M+H)+	1
PCaeC42.4	PC.ae.C42.4	852,7	184	0,83025	0,7745	(M+H)+	1
PCaeC42.5	PC.ae.C42.5	850,7	184	1,765	1,76	(M+H)+	1
PCaeC44.3	PC.ae.C44.3	882,7	184	0,28225	0,219	(M+H)+	1
PCaeC44.4	PC.ae.C44.4	880,7	184	0,4375	0,412	(M+H)+	1
PCaeC44.5	PC.ae.C44.5	878,7	184	1,4725	1,51	(M+H)+	1
PCaeC44.6	PC.ae.C44.6	876,7	184	0,85425	0,8785	(M+H)+	1
SM.OH.C14.1	SM.C18.1.OH.C14.1	689,5	184	4,4825	4,26	(M+H)+	1
SM.OH.C16.1	SM.C18.1.OH.C16.1	717,6	184	2,065	2,075	(M+H)+	1
SM.OH.C22.1	SM.C18.1.OH.C22.1	801,6	184	8,58	8,365	(M+H)+	1

SM.OH.C22.2	SM.C18.1.OH.C22.2	799,6	184	6,26	6,14	(M+H)+	1
SM.OH.C24.1	SM.C18.1.OH.C24.1	829,7	184	0,9405	0,9585	(M+H)+	1
SMC16.0	SM.C18.1.C16.0	703,6	184	76,4	75	(M+H)+	1
SMC16.1	SM.C18.1.C16.1	701,6	184	10,525	10,55	(M+H)+	1
SMC18.0	SM.C18.1.C18.0	731,6	184	14,575	14,35	(M+H)+	1
SMC18.1	SM.C18.1.C18.1	729,6	184	7,1225	7,025	(M+H)+	1
SMC20.2	SM.C18.1.C20.2	755,6	184	0,322	0,282	(M+H)+	1
SMC22.3	SM.C18.1.C22.3	781,6	184	1,7625	1,755	(M+H)+	1
SMC24.0	SM.C18.1.C24.0	815,7	184	14,8	14,3	(M+H)+	1
SMC24.1	SM.C18.1.C24.1	813,7	184	33	32,15	(M+H)+	1
SMC26.0	SM.C18.1.C26.0	843,7	184	0,07225	0,0815	(M+H)+	1
SMC26.1	SM.C18.1.C26.1	841,7	184	0,28175	0,2855	(M+H)+	1
Sum of Hexoses	Sum of Hexoses	179	89	22237,5	22592	(M+H)+	1

ID metabolite identity metabolomics laboratory. Sofia ID metabolite identity Generation R Study group. Q1/Q3 Quadrupole 1 and 3, CP1/CP2 Calibrators 1 and 2, MSI ID Metabolomics Standards Initiative identification.
This table is adapted from: Voerman, E., Jaddoe, V. W. V., Uhl, O., & Shokry, E. (2020). A population based resource for intergenerational metabolomics analysis in pregnant women and their children : the Generation R Study. *Metabolomics*, 1 – 26. Voerman et al. 2020 describes the acquisition, processing and structure of the metabolomics data in the Generation R study cohort.

REFERENCES

1. Voerman E, Jaddoe VWV, Uhl O, et al. A population-based resource for intergenerational metabolomics analyses in pregnant women and their children: the Generation R Study. *Metabolomics* 2020; **16**(4): 43-.
2. Hellmuth C, Uhl O, Standl M, et al. Cord Blood Metabolome Is Highly Associated with Birth Weight, but Less Predictive for Later Weight Development. *Obes Facts* 2017; **10**(2): 85-100.
3. Hellmuth C, Weber M, Koletzko B, Peissner W. Nonesterified Fatty Acid Determination for Functional Lipidomics: Comprehensive Ultrahigh Performance Liquid Chromatography–Tandem Mass Spectrometry Quantitation, Qualification, and Parameter Prediction. *Analytical Chemistry* 2012; **84**(3): 1483-90.
4. Uhl O, Fleddermann M, Hellmuth C, Demmelmair H, Koletzko B. Phospholipid Species in Newborn and 4 Month Old Infants after Consumption of Different Formulas or Breast Milk. *PLoS One* 2016; **11**(8): e0162040-e.

Table S2. Maternal early-pregnancy serum metabolite concentrations

Maternal early-pregnancy metabolite or metabolite group	Median (95% range), μmol/L
**Amino acids (AA)**	**2880.7 (1990.8, 3971.2)**
**Branched-chain AA**	**430.7 (269.4, 723.9)**
**Aromatic AA**	**214.4 (139.3, 232.3)**
**Essential AA**	**1022.3 (668.2, 1531.1)**
**Non-essential AA**	**1848.0 (1249.8, 2482.6)**
Alanine	404.8 (257.3, 626.6)
Arginine	91.4 (57.3, 136.8)
Asparagine	63.1 (41.1, 92.2)
Aspartic acid	37.6 (20.1, 63.5)
Citrulline	19.7 (10.5, 33.4)
Glutamine	447.0 (277.1, 738.4)
Glucose	90.6 (45.1, 157.3)
Glycine	218.4 (135.0, 332.1)
Histine	102.9 (60.3, 156.3)
Isoleucine	61.5 (30.9, 115.2)
Leucine	131.8 (78.3, 232.9)
Lysine	161.1 (95.0, 260.5)
Methionine	22.9 (13.1, 39.9)
Ornitine	57.3 (31.3, 97.9)
Phenylalanine	87.5 (56.4, 128.1)
Proline	194.9 (100.7, 366.1)
Tryptophan	60.7 (37.9, 93.4)
Serine	111.0 (62.4, 171.6)
Threonine	138.5 (81.3, 234.3)
Tyrosine	64.1 (35.4, 113.0)
Valine	239.8 (145.5, 382.0)
Cystein	15.3 (5.0, 29.9)
**Non-esterified fatty acids (NEFA)**	**143.9 (52.9, 458.9)**
**Saturated NEFA**	**58.8 (18.5, 186.2)**
**Mono-unsaturated NEFA**	**49.1 (15.7, 174.8)**
**Poly-unsaturated NEFA**	**34.8 (13.9, 99.9)**
NEFA_14_0	4.4 (1.2, 15.2)
NEFA_14_1	0.6 (0.1, 2.6)
NEFA_15_0	0.9 (0.2, 3.0)
NEFA_16_0	41.7 (10.2, 132.1)
NEFA_16_1	4.4 (1.3, 18.3)
NEFA_16_2	0.2 (0.0, 0.6)
NEFA_17_0	1.0 (0.3, 3.0)
NEFA_17_1	0.4 (0.0, 1.6)
NEFA_17_2	0.0 (0.0, 0.1)
NEFA_18_0	10.7 (1.4, 34.9)
NEFA_18_1	42.4 (12.7, 150.9)
NEFA_18_2	23.4 (8.8, 75.0)
NEFA_18_3	2.5 (0.6, 9.1)

NEFA_19_1	0.2 (0.1, 0.7)
NEFA_20_1	0.6 (0.1, 2.0)
NEFA_20_2	0.4 (0.1, 1.3)
NEFA_20_3	0.7 (0.2, 1.8)
NEFA_20_4	4.3 (1.8, 9.0)
NEFA_20_5	0.3 (0.1, 0.8)
NEFA_22_3	0.0 (0.0, 0.1)
NEFA_22_4	0.3 (0.1, 0.7)
NEFA_22_5	0.4 (0.2, 1.1)
NEFA_22_6	1.5 (0.4, 4.4)
NEFA_24_0	0.2 (0.1, 0.6)
NEFA_24_1	0.1 (0.0, 0.3)
NEFA_24_2	0.0 (0.0, 0.1)
NEFA_24_4	0.0 (0.0, 0.1)
NEFA_24_5	0.0 (0.0, 0.1)
NEFA_26_0	0.2 (0.1, 0.5)
NEFA_26_1	0.1 (0.0, 0.3)
NEFA_26_2	0.1 (0.0, 0.2)
**Acyl-lysosphophatidylcholines (LysoPCa)**	**182.6 (81.4, 229.7)**
**Saturated LysoPCa**	**127.3 (70.8, 214.7)**
**Mono-unsaturated LysoPCa**	**19.1 (10.3, 32.4)**
**Poly-unsaturated LysoPCa**	**35.9 (19.2, 66.9)**
Lyso.PC.a.C14.0	3.3 (1.0, 7.3)
Lyso.PC.a.C16.0	98.6 (54.8, 170.6)
Lyso.PC.a.C16.1	2.1 (0.9, 4.0)
Lyso.PC.a.C18.0	24.0 (13.0, 41.6)
Lyso.PC.a.C18.1	16.9 (9.4, 29.2)
Lyso.PC.a.C18.2	23.9 (11.8, 47.8)
Lyso.PC.a.C18.3	0.4 (0.1, 1.0)
Lyso.PC.a.C20.3	2.5 (1.2, 4.8)
Lyso.PC.a.C20.4	6.1 (2.9, 11.0)
Lyso.PC.a.C20.5	0.4 (0.1, 1.0)
Lyso.PC.a.C22.6	2.3 (1.1, 4.3)
**Alkyl-lysophosphatidylcholines (LysoPCe)**	**3.0 (1.6, 5.1)**
**Saturated LysoPCe**	**2.7 (1.4, 4.8)**
**Mono-unsaturated LysoPCe**	**0.3 (0.1, 0.5)**
Lyso.PC.e.C16.0	1.0 (0.4, 1.9)
Lyso.PC.e.C18.0	1.8 (0.9, 3.0)
Lyso.PC.e.C18.1	0.3 (0.1, 0.5)
**Diacyl-phosphatidylcholines (PCaa)**	**1813.6 (1030.4, 3060.6)**
**Saturated PCaa**	**27.6 (16.0, 45.2)**
**Mono-unsaturated PCaa**	**275.1 (144.5, 518.0)**
**Poly-unsaturated PCaa**	**1518.4 (862.2, 2551.4)**
PC.aa.C30.0	5.4 (2.3, 11.4)
PC.aa.C30.3	0.2 (0.1, 0.4)
PC.aa.C32.0	13.9 (8.3, 23.2)

PC.aa.C32.1	14.7 (6.4, 36.0)
PC.aa.C32.2	5.6 (2.0, 11.7)
PC.aa.C32.3	0.4 (0.2, 0.8)
PC.aa.C34.1	217.2 (110.5, 405.8)
PC.aa.C34.2	442.4 (239.8, 731.0)
PC.aa.C34.3	17.6 (8.4, 35.4)
PC.aa.C34.4	2.3 (0.9, 5.0)
PC.aa.C34.5	0.2 (0.1, 0.5)
PC.aa.C36.0	1.7 (0.7, 3.4)
PC.aa.C36.1	41.5 (22.1, 76.9)
PC.aa.C36.2	212.5 (116.4, 352.1)
PC.aa.C36.3	172.7 (91.0, 318.3)
PC.aa.C36.4	226.1 (120.5, 407.2)
PC.aa.C36.5	20.0 (8.0, 45.1)
PC.aa.C36.6	1.3 (0.4, 2.8)
PC.aa.C38.0	3.8 (1.8, 6.9)
PC.aa.C38.2	7.2 (3.3, 13.1)
PC.aa.C38.3	51.0 (24.8, 104.0)
PC.aa.C38.4	108.6 (57.5, 194.3)
PC.aa.C38.5	54.9 (28.3, 101.5)
PC.aa.C38.6	126.3 (59.5, 232.5)
PC.aa.C40.0	1.3 (0.5, 2.5)
PC.aa.C40.1	0.6 (0.2, 1.2)
PC.aa.C40.2	0.4 (0.1, 0.7)
PC.aa.C40.3	0.6 (0.2, 1.2)
PC.aa.C40.4	3.9 (1.9, 8.0)
PC.aa.C40.5	12.3 (6.1, 24.1)
PC.aa.C40.6	38.2 (18.1, 70.0)
PC.aa.C42.0	1.0 (0.5, 1.7)
PC.aa.C42.5	0.6 (0.3, 1.1)
PC.aa.C43.6	3.4 (1.9, 6.0)
PC.aa.C44.12	0.8 (0.4, 1.4)
**Acyl-alkyl-phosphatidylcholines (PCae)**	**187.3 (111.1, 305.6)**
**Saturated PCae**	**27.7 (14.8, 50.3)**
**Mono-unsaturated PCae**	**20.0 (11.1, 33.4)**
**Poly-unsaturated PCae**	**138.6 (81.4, 229.7)**
PC.ae.C30.0	0.5 (0.2, 1.0)
PC.ae.C32.0	3.7 (2.1, 6.5)
PC.ae.C32.1	2.7 (1.5, 4.7)
PC.ae.C32.2	0.6 (0.3, 1.2)
PC.ae.C34.0	1.5 (0.7, 2.7)
PC.ae.C34.1	9.6 (5.3, 16.7)
PC.ae.C34.2	10.6 (5.5, 18.2)
PC.ae.C34.3	8.1 (4.1, 14.3)
PC.ae.C34.4	0.3 (0.1, 0.6)
PC.ae.C36.0	0.7 (0.4, 1.4)

PC.ae.C36.1	5.5 (2.6, 9.5)
PC.ae.C36.2	14.7 (8.0, 24.7)
PC.ae.C36.3	8.6 (4.4, 15.4)
PC.ae.C36.4	16.4 (9.3, 29.3)
PC.ae.C36.5	10.8 (5.5, 19.4)
PC.ae.C38.0	2.6 (1.1, 5.6)
PC.ae.C38.2	1.9 (0.9, 3.4)
PC.ae.C38.3	4.4 (2.3, 8.0)
PC.ae.C38.4	13.3 (7.5, 23.7)
PC.ae.C38.5	17.7 (9.4, 30.5)
PC.ae.C38.6	8.0 (3.8, 14.2)
PC.ae.C40.0	18.3 (9.2, 35.9)
PC.ae.C40.1	1.6 (0.7, 2.8)
PC.ae.C40.2	1.7 (0.7, 3.2)
PC.ae.C40.3	1.0 (0.5, 1.9)
PC.ae.C40.4	2.9 (1.7, 5.1)
PC.ae.C40.5	3.9 (2.2, 6.6)
PC.ae.C40.6	5.5 (2.9, 9.6)
PC.ae.C42.1	0.4 (0.2, 0.8)
PC.ae.C42.3	1.0 (0.5, 1.7)
PC.ae.C42.4	1.4 (0.7, 2.4)
PC.ae.C42.5	3.1 (1.6, 5.9)
PC.ae.C42.6	2.1 (1.1, 3.6)
**Sphingomyelines (SM)**	**410.4 (252.8, 621.5)**
**Mono-unsaturated SM**	**200.9 (129.7, 299.5)**
**Poly-unsaturated SM**	**209.8 (120.5, 332.2)**
SM.a.C30.1	0.5 (0.2, 0.9)
SM.a.C32.1	8.7 (4.9, 14.5)
SM.a.C32.2	0.9 (0.4, 1.5)
SM.a.C33.1	6.4 (3.8, 10.3)
SM.a.C34.1	109.8 (70.1, 167.4)
SM.a.C34.2	17.7 (10.5, 29.5)
SM.a.C35.1	0.8 (0.4, 1.3)
SM.a.C36.1	22.8 (13.4, 35.7)
SM.a.C36.2	11.1 (6.4, 19.6)
SM.a.C36.3	1.0 (0.5, 1.7)
SM.a.C37.1	2.1 (0.9, 3.7)
SM.a.C38.2	20.9 (9.9, 36.4)
SM.a.C38.3	0.7 (0.3, 1.3)
SM.a.C39.1	5.7 (2.7, 9.4)
SM.a.C39.2	1.8 (0.8, 3.0)
SM.a.C40.2	32.4 (17.1, 54.7)
SM.a.C40.5	0.8 (0.3, 2.0)
SM.a.C41.1	16.2 (9.3, 25.6)
SM.a.C41.2	13.9 (7.5, 21.6)
SM.a.C42.1	23.1 (14.7, 39.1)

SM.a.C42.2	62.4 (35.4, 101.2)
SM.a.C42.3	27.0 (14.7, 45.8)
SM.a.C42.4	8.1 (3.9, 15.1)
SM.a.C42.6	5.0 (2.3, 9.3)
SM.a.C43.1	1.4 (0.8, 2.4)
SM.a.C43.2	2.7 (1.3, 4.8)
SM.a.C44.6	1.8 (0.8, 3.4)
SM.e.C36.2	0.4 (0.2, 0.8)
SM.e.C38.3	0.3 (0.1, 0.6)
SM.e.C40.5	0.4 (0.1, 0.7)
**Free Carn**	**25.0 (13.6, 38.2)**
**Acyl-carnitine (Carn.a)**	**4.9 (2.9, 8.1)**
**Small-chain Carn.a**	**3.5 (1.9, 6.3)**
**Medium-chain Carn.a**	**0.6 (0.3, 1.2)**
**Large-chain Carn.a**	**0.8 (0.4, 1.4)**
Carn.a.C10.0	0.1 (0.0, 0.4)
Carn.a.C10.1	0.1 (0.0, 0.2)
Carn.a.C12.0	0.1 (0.0, 0.2)
Carn.a.C14.1	0.0 (0.0, 0.1)
Carn.a.C14.2	0.0 (0.0, 0.1)
Carn.a.C15.0	0.0 (0.0, 0.1)
Carn.a.C16.0	0.1 (0.1, 0.3)
Carn.a.C16.0.Oxo	0.0 (0.0, 0.0)
Carn.a.C16.1	0.1 (0.0, 0.2)
Carn.a.C16.2	0.0 (0.0, 0.1)
Carn.a.C18.0	0.1 (0.0, 0.2)
Carn.a.C18.1	0.1 (0.0, 0.2)
Carn.a.C18.2	0.1 (0.0, 0.1)
Carn.a.C18.2.OH	0.0 (0.0, 0.1)
Carn.a.C2.0	2.8 (1.4, 5.4)
Carn.a.C20.0	0.0 (0.0, 0.1)
Carn.a.C20.1	0.0 (0.0, 0.0)
Carn.a.C20.3	0.1 (0.0, 0.1)
Carn.a.C20.4	0.0 (0.0, 0.0)
Carn.a.C3.0	0.3 (0.2, 0.5)
Carn.a.C3.0.DC	0.1 (0.0, 0.1)
Carn.a.C4.0	0.2 (0.1, 0.4)
Carn.a.C5.0	0.1 (0.1, 0.2)
Carn.a.C6.0	0.0 (0.0, 0.1)
Carn.a.C6.0.OH	0.0 (0.0, 0.1)
Carn.a.C8.0	0.1 (0.0, 0.2)
Carn.a.C8.1	0.1 (0.0, 0.1)
Carn.a.C9.0	0.0 (0.0, 0.1)

Values represent medians (95% range) of maternal early-pregnancy metabolite concentrations in serum (μmol/L).

Table S3. Associations of maternal early-pregnancy individual metabolites with systolic and diastolic blood pressure in early-, mid- and late pregnancy. Basic model.

	**Differences in systolic blood pressure in mmHg (95% confidence interval)**						**Differences in diastolic blood pressure in mmHg (95% confidence interval)**					
**Metabolite**	**Early pregnancy** N = 803	**P-value**	**Mid pregnancy** N = 793	**P-value**	**Late pregnancy** N = 800	**P-value**	**Early pregnancy** N = 803	**P-value**	**Mid pregnancy** N = 793	**P-value**	**Late pregnancy** N = 800	**P-value**
Ala	6.94 (2.86 - 16.86)	<0.001*	4.05 (1.72 - 9.52)	0.011*	3.95 (1.85 - 8.43)	0.003*	2.01 (1.02 - 3.98)	0.078	2.5 (1.29 - 4.83)	0.027*	4.25 (2.26 - 7.99)	<0.001*
Arg	9.65 (3.99 - 23.32)	0.000*	4.42 (1.87 - 10.42)	0.006*	4.68 (2.2 - 9.96)	0.001*	4.03 (2.05 - 7.92)	0.000*	2.42 (1.25 - 4.69)	0.035*	3.46 (1.84 - 6.51)	0.001*
Asn	2.2 (0.9 - 5.38)	0.154	1 (0.42 - 2.37)	0.999	2.15 (1 - 4.6)	0.102	0.72 (0.36 - 1.42)	0.444	0.79 (0.41 - 1.54)	0.612	1.46 (0.77 - 2.76)	0.308
Asp	7.23 (2.97 - 17.58)	<0.001*	8.15 (3.51 - 18.96)	<0.001*	8.5 (4.02 - 17.99)	<0.001*	6.93 (3.54 - 13.54)	<0.001*	5.53 (2.89 - 10.57)	<0.001*	9.84 (5.3 - 18.28)	<0.001*
Cit	0.87 (0.36 - 2.14)	0.824	0.65 (0.27 - 1.53)	0.507	1 (0.47 - 2.15)	0.996	1.03 (0.52 - 2.04)	0.974	0.64 (0.33 - 1.24)	0.319	1.07 (0.56 - 2.03)	0.863
Gln	1.14 (0.47 - 2.8)	0.829	0.73 (0.31 - 1.73)	0.677	0.57 (0.26 - 1.23)	0.247	0.76 (0.38 - 1.51)	0.537	0.47 (0.25 - 0.92)	0.070	0.5 (0.26 - 0.95)	0.065
Glu	3.85 (1.57 - 9.44)	0.013*	6.36 (2.72 - 14.87)	<0.001*	6.09 (2.87 - 12.94)	<0.001*	4.4 (2.24 - 8.66)	<0.001*	4.21 (2.19 - 8.09)	<0.001*	7.58 (4.07 - 14.14)	<0.001*
Gly	0.79 (0.32 - 1.93)	0.706	1.16 (0.49 - 2.72)	0.856	1.47 (0.69 - 3.16)	0.451	0.83 (0.42 - 1.65)	0.693	1.12 (0.58 - 2.17)	0.827	1.93 (1.02 - 3.66)	0.076
His	2.63 (1.07 - 6.42)	0.080	1.51 (0.64 - 3.57)	0.540	2.21 (1.03 - 4.72)	0.092	0.9 (0.46 - 1.79)	0.847	0.98 (0.5 - 1.9)	0.975	1.87 (0.99 - 3.53)	0.091
Ile	4.14 (1.69 - 10.12)	0.009*	2 (0.84 - 4.73)	0.260	1.79 (0.83 - 3.84)	0.232	1 (0.5 - 1.97)	0.990	1.32 (0.68 - 2.58)	0.548	2.01 (1.06 - 3.8)	0.062
Leu	3.69 (1.5 - 9.04)	0.017*	2.12 (0.9 - 5)	0.226	2.29 (1.07 - 4.89)	0.076	1.06 (0.53 - 2.1)	0.927	1.39 (0.72 - 2.7)	0.472	2.33 (1.23 - 4.4)	0.023*
Lys	1.43 (0.58 - 3.5)	0.563	2.5 (1.06 - 5.9)	0.123	3.78 (1.77 - 8.04)	0.004*	0.84 (0.42 - 1.66)	0.707	2.23 (1.15 - 4.32)	0.054	3.37 (1.79 - 6.33)	0.001*
Met	2.9 (1.18 - 7.08)	0.055	1.66 (0.7 - 3.92)	0.424	1.58 (0.74 - 3.39)	0.354	1.05 (0.53 - 2.07)	0.942	1 (0.51 - 1.94)	0.998	1.4 (0.74 - 2.65)	0.361
Orn	1.55 (0.63 - 3.82)	0.471	1.94 (0.82 - 4.57)	0.267	2.86 (1.34 - 6.12)	0.024*	0.8 (0.4 - 1.58)	0.613	1.42 (0.73 - 2.74)	0.446	2.98 (1.58 - 5.63)	0.003*
Phe	9.14 (3.77 - 22.14)	<0.001*	4.44 (1.89 - 10.42)	0.006*	3.46 (1.62 - 7.38)	0.007*	2.52 (1.28 - 4.98)	0.020*	2.28 (1.18 - 4.4)	0.049*	3.61 (1.92 - 6.79)	<0.001*

Pro	3.44 (1.4 - 8.42)	0.023*	2.3 (0.98 - 5.42)	0.162	1.65 (0.77 - 3.53)	0.310	1.29 (0.65 - 2.56)	0.558	1.11 (0.57 - 2.15)	0.837	1.86 (0.98 - 3.52)	0.093
Trp	6.09 (2.49 - 14.86)	0.001*	2.67 (1.13 - 6.31)	0.101	2.61 (1.22 - 5.57)	0.038*	0.98 (0.5 - 1.95)	0.982	1.02 (0.52 - 1.97)	0.987	1.48 (0.78 - 2.8)	0.294
Ser	1.68 (0.69 - 4.12)	0.375	1.18 (0.5 - 2.79)	0.830	2.47 (1.15 - 5.31)	0.051	1.32 (0.67 - 2.62)	0.533	0.99 (0.51 - 1.92)	0.997	2.2 (1.16 - 4.17)	0.033*
Thr	3.58 (1.46 - 8.81)	0.019*	2.66 (1.12 - 6.31)	0.102	2.91 (1.36 - 6.21)	0.022*	1.45 (0.73 - 2.88)	0.385	1.75 (0.9 - 3.41)	0.210	2.4 (1.27 - 4.52)	0.019*
Tyr	7.9 (3.25 - 19.21)	<0.001*	2.81 (1.19 - 6.62)	0.077	3.27 (1.53 - 6.99)	0.010*	1.8 (0.91 - 3.57)	0.142	1.39 (0.72 - 2.69)	0.472	2.34 (1.24 - 4.42)	0.022*
Val	7.24 (2.97 - 17.66)	<0.001*	3.26 (1.39 - 7.65)	0.035*	3.35 (1.57 - 7.15)	0.009*	1.77 (0.89 - 3.51)	0.155	1.63 (0.84 - 3.16)	0.273	2.71 (1.44 - 5.11)	0.007*
Cys	1.68 (0.69 - 4.13)	0.375	2.23 (0.95 - 5.24)	0.183	2.79 (1.3 - 5.97)	0.029*	0.78 (0.39 - 1.54)	0.566	1.51 (0.78 - 2.91)	0.354	2.14 (1.13 - 4.06)	0.040*
NEFA_14_0	1.39 (0.57 - 3.42)	0.575	1.15 (0.49 - 2.7)	0.864	0.78 (0.37 - 1.68)	0.655	1.97 (0.99 - 3.89)	0.090	1.36 (0.7 - 2.63)	0.508	1.37 (0.72 - 2.58)	0.400
NEFA_14_1	1.16 (0.47 - 2.84)	0.816	1.3 (0.55 - 3.05)	0.731	1.17 (0.55 - 2.52)	0.778	3.54 (1.8 - 6.97)	0.001*	2.51 (1.3 - 4.84)	0.026*	1.95 (1.03 - 3.7)	0.071
NEFA_15_0	1.18 (0.48 - 2.89)	0.792	1.02 (0.43 - 2.4)	0.975	0.57 (0.27 - 1.22)	0.239	1.64 (0.83 - 3.25)	0.218	1.29 (0.67 - 2.5)	0.571	0.89 (0.47 - 1.68)	0.762
NEFA_16_0	1.86 (0.76 - 4.55)	0.283	1.9 (0.81 - 4.47)	0.278	1.13 (0.53 - 2.43)	0.827	2.96 (1.5 - 5.83)	0.006*	2.34 (1.21 - 4.51)	0.040*	2.21 (1.17 - 4.18)	0.032*
NEFA_16_1	1.27 (0.52 - 3.1)	0.706	1.6 (0.68 - 3.77)	0.455	1.41 (0.66 - 3.01)	0.512	4.27 (2.17 - 8.38)	<0.001*	3.26 (1.7 - 6.27)	0.003*	3.47 (1.84 - 6.52)	0.001*
NEFA_16_2	1.72 (0.7 - 4.21)	0.356	1.66 (0.71 - 3.9)	0.421	1.25 (0.58 - 2.68)	0.681	3.19 (1.62 - 6.29)	0.003*	2.24 (1.16 - 4.31)	0.052	2.59 (1.38 - 4.89)	0.010*
NEFA_17_0	1.21 (0.5 - 2.98)	0.760	1.14 (0.48 - 2.68)	0.872	0.87 (0.4 - 1.87)	0.807	1.97 (1 - 3.89)	0.089	1.75 (0.9 - 3.39)	0.206	1.38 (0.73 - 2.61)	0.390
NEFA_17_1	1.71 (0.7 - 4.19)	0.357	1.46 (0.62 - 3.43)	0.586	1.04 (0.49 - 2.23)	0.952	3.01 (1.53 - 5.93)	0.005*	2.63 (1.37 - 5.07)	0.018*	1.94 (1.02 - 3.66)	0.075
NEFA_17_2	1.71 (0.7 - 4.19)	0.357	0.97 (0.41 - 2.29)	0.975	0.93 (0.43 - 2)	0.905	1.96 (0.99 - 3.87)	0.090	1.34 (0.7 - 2.6)	0.518	1.78 (0.94 - 3.37)	0.121
NEFA_18_0	1.53 (0.62 - 3.75)	0.483	1.08 (0.46 - 2.55)	0.934	0.89 (0.41 - 1.9)	0.832	2.22 (1.13 - 4.39)	0.043*	1.35 (0.7 - 2.61)	0.515	1.16 (0.61 - 2.19)	0.711
NEFA_18_1	1.36 (0.56 - 3.34)	0.598	1.39 (0.59 - 3.28)	0.637	1.35 (0.63 - 2.9)	0.559	3.15 (1.6 - 6.21)	0.004*	2.16 (1.12 - 4.16)	0.062	2.59 (1.37 - 4.89)	0.010*
NEFA_18_2	1.51 (0.62 - 3.7)	0.492	1.23 (0.52 - 2.9)	0.786	1.06 (0.5 - 2.28)	0.916	2.58 (1.31 - 5.08)	0.017*	1.62 (0.84 - 3.13)	0.283	1.96 (1.03 - 3.7)	0.071

NEFA_18_3	1.67 (0.68 - 4.1)	0.375	1.27 (0.54 - 2.98)	0.765	0.82 (0.38 - 1.75)	0.714	1.88 (0.95 - 3.71)	0.116	1.29 (0.67 - 2.5)	0.571	1.54 (0.82 - 2.92)	0.238
NEFA_19_1	1.02 (0.42 - 2.51)	0.962	0.94 (0.4 - 2.22)	0.962	0.79 (0.37 - 1.69)	0.658	2.03 (1.03 - 4.01)	0.075	1.66 (0.86 - 3.21)	0.256	1.38 (0.73 - 2.62)	0.382
NEFA_20_1	1.45 (0.59 - 3.57)	0.542	1.1 (0.47 - 2.59)	0.913	1.36 (0.63 - 2.91)	0.558	3.04 (1.54 - 5.99)	0.005*	1.85 (0.96 - 3.57)	0.159	2.81 (1.49 - 5.31)	0.005*
NEFA_20_2	1.41 (0.58 - 3.46)	0.563	1.6 (0.68 - 3.75)	0.455	1.78 (0.83 - 3.81)	0.234	3.3 (1.68 - 6.5)	0.002*	3 (1.56 - 5.77)	0.006*	3.32 (1.76 - 6.25)	0.001*
NEFA_20_3	3.78 (1.55 - 9.22)	0.014*	3.96 (1.69 - 9.27)	0.011*	2.75 (1.28 - 5.87)	0.030*	4.66 (2.37 - 9.14)	<0.001*	4.43 (2.31 - 8.49)	<0.001*	3.58 (1.9 - 6.74)	0.001*
NEFA_20_4	3.45 (1.41 - 8.44)	0.022*	3.52 (1.5 - 8.22)	0.024*	3.09 (1.45 - 6.59)	0.015*	5.27 (2.69 - 10.34)	<0.001*	3.37 (1.75 - 6.46)	0.002*	5.03 (2.69 - 9.41)	<0.001*
NEFA_20_5	2.07 (0.84 - 5.09)	0.194	1.21 (0.52 - 2.86)	0.805	1.55 (0.73 - 3.33)	0.371	1.83 (0.92 - 3.62)	0.129	1.05 (0.54 - 2.04)	0.916	2.29 (1.21 - 4.32)	0.025*
NEFA_22_3	2.04 (0.83 - 4.99)	0.205	3.36 (1.43 - 7.89)	0.032*	3.58 (1.67 - 7.67)	0.006*	4.12 (2.1 - 8.1)	<0.001*	4.72 (2.45 - 9.07)	<0.001*	3.96 (2.1 - 7.47)	<0.001*
NEFA_22_4	4.15 (1.7 - 10.14)	0.009*	4.89 (2.1 - 11.4)	0.003*	3.62 (1.7 - 7.72)	0.006*	6.75 (3.45 - 13.18)	<0.001*	5.31 (2.78 - 10.15)	<0.001*	5.47 (2.93 - 10.24)	<0.001*
NEFA_22_5	2.2 (0.9 - 5.38)	0.153	2.01 (0.85 - 4.71)	0.258	1.99 (0.93 - 4.26)	0.148	4.15 (2.11 - 8.16)	<0.001*	2.75 (1.43 - 5.29)	0.013*	3.2 (1.7 - 6.03)	0.002*
NEFA_22_6	1.66 (0.68 - 4.06)	0.383	1.17 (0.5 - 2.75)	0.847	1.36 (0.64 - 2.93)	0.558	3.23 (1.64 - 6.36)	0.003*	1.71 (0.88 - 3.3)	0.228	2.49 (1.32 - 4.7)	0.014*
NEFA_24_0	2.14 (0.87 - 5.24)	0.172	1.66 (0.7 - 3.92)	0.423	1.06 (0.5 - 2.28)	0.916	1.11 (0.56 - 2.19)	0.847	0.87 (0.45 - 1.69)	0.789	0.96 (0.51 - 1.81)	0.913
NEFA_24_1	0.81 (0.33 - 1.98)	0.733	0.56 (0.24 - 1.32)	0.345	0.91 (0.42 - 1.96)	0.877	1.13 (0.57 - 2.23)	0.818	0.67 (0.35 - 1.3)	0.375	1.48 (0.78 - 2.81)	0.294
NEFA_24_2	1.07 (0.44 - 2.64)	0.904	1.55 (0.66 - 3.64)	0.505	1.48 (0.69 - 3.18)	0.445	2.54 (1.29 - 5.02)	0.019*	1.67 (0.86 - 3.22)	0.253	1.58 (0.84 - 3)	0.211
NEFA_24_4	4.29 (1.76 - 10.5)	0.008*	3.96 (1.7 - 9.26)	0.011*	3.5 (1.64 - 7.46)	0.007*	4.11 (2.09 - 8.1)	<0.001*	4.08 (2.13 - 7.82)	<0.001*	3.48 (1.85 - 6.55)	0.001*
NEFA_24_5	4.07 (1.66 - 9.95)	0.010*	2.84 (1.21 - 6.67)	0.073	4.09 (1.92 - 8.73)	0.002*	4.63 (2.35 - 9.1)	<0.001*	3.97 (2.07 - 7.62)	<0.001*	4.57 (2.43 - 8.57)	<0.001*
NEFA_26_0	3.92 (1.61 - 9.58)	0.012*	2.31 (0.98 - 5.46)	0.161	2.18 (1.02 - 4.67)	0.096	2.54 (1.29 - 5.01)	0.019*	1.6 (0.82 - 3.1)	0.291	1.86 (0.98 - 3.52)	0.093
NEFA_26_1	1.93 (0.79 - 4.72)	0.252	3.08 (1.31 - 7.24)	0.050	2.18 (1.02 - 4.67)	0.096	2.68 (1.36 - 5.28)	0.013*	2.27 (1.17 - 4.4)	0.049*	2.07 (1.1 - 3.92)	0.049*
NEFA_26_2	1.54 (0.63 - 3.77)	0.478	2.52 (1.07 - 5.92)	0.120	1.97 (0.92 - 4.22)	0.151	2.29 (1.16 - 4.52)	0.036*	2.14 (1.11 - 4.14)	0.066	1.71 (0.91 - 3.24)	0.145

lyso.PC.a.C14.0	28.87 (12.07 - 69.03)	<0.001*	13.27 (5.68 - 30.97)	<0.001*	10.99 (5.19 - 23.27)	<0.001*	4.46 (2.26 - 8.79)	<0.001*	5.01 (2.59 - 9.67)	<0.001*	6.29 (3.35 - 11.81)	<0.001*
lyso.PC.a.C16.0	7.08 (2.9 - 17.28)	<0.001*	7.51 (3.22 - 17.52)	<0.001*	5.6 (2.61 - 11.98)	<0.001*	3.06 (1.55 - 6.04)	0.005*	4.22 (2.2 - 8.12)	<0.001*	5.84 (3.1 - 10.99)	<0.001*
lyso.PC.a.C16.1	18.47 (7.61 - 44.8)	<0.001*	15.39 (6.64 - 35.69)	<0.001*	11.54 (5.45 - 24.42)	<0.001*	5.67 (2.87 - 11.21)	<0.001*	6.41 (3.34 - 12.29)	<0.001*	8.57 (4.59 - 16.03)	<0.001*
lyso.PC.a.C18.0	3.33 (1.35 - 8.24)	0.028*	4.9 (2.09 - 11.49)	0.003*	3.47 (1.62 - 7.43)	0.007*	2.05 (1.03 - 4.08)	0.075	2.25 (1.17 - 4.36)	0.051	3.26 (1.73 - 6.15)	0.001*
lyso.PC.a.C18.1	6.21 (2.54 - 15.18)	<0.001*	3.32 (1.41 - 7.84)	0.034*	2.63 (1.23 - 5.64)	0.037*	2.15 (1.09 - 4.27)	0.054	1.86 (0.96 - 3.61)	0.159	2.81 (1.49 - 5.32)	0.005*
lyso.PC.a.C18.2	3.66 (1.49 - 8.98)	0.017*	1.61 (0.69 - 3.79)	0.455	1.19 (0.56 - 2.55)	0.753	0.97 (0.49 - 1.92)	0.967	0.64 (0.33 - 1.24)	0.319	0.86 (0.46 - 1.63)	0.705
lyso.PC.a.C18.3	6.13 (2.52 - 14.94)	0.001*	2 (0.85 - 4.73)	0.258	2.97 (1.39 - 6.37)	0.020*	1.18 (0.59 - 2.33)	0.729	0.92 (0.48 - 1.79)	0.873	1.96 (1.04 - 3.72)	0.071
lyso.PC.a.C20.3	16.31 (6.78 - 39.24)	<0.001*	9.37 (4.03 - 21.78)	<0.001*	8.75 (4.15 - 18.46)	<0.001*	4.74 (2.41 - 9.31)	<0.001*	4.33 (2.26 - 8.33)	<0.001*	4.12 (2.19 - 7.73)	<0.001*
lyso.PC.a.C20.4	10.73 (4.38 - 26.28)	<0.001*	8.19 (3.51 - 19.12)	<0.001*	4.91 (2.31 - 10.46)	0.001*	6.55 (3.31 - 12.93)	<0.001*	4.7 (2.44 - 9.03)	<0.001*	5.1 (2.72 - 9.58)	<0.001*
lyso.PC.a.C20.5	2.64 (1.07 - 6.54)	0.082	1.28 (0.54 - 3.01)	0.745	2.53 (1.18 - 5.41)	0.044*	1.35 (0.68 - 2.69)	0.507	1.34 (0.7 - 2.6)	0.518	2.14 (1.13 - 4.03)	0.040*
lyso.PC.a.C22.6	2.83 (1.15 - 6.97)	0.063	1.54 (0.65 - 3.64)	0.510	1.57 (0.73 - 3.37)	0.365	2.04 (1.03 - 4.05)	0.075	1.42 (0.73 - 2.75)	0.446	1.77 (0.93 - 3.36)	0.123
lyso.PC.e.C16.0	4.9 (2 - 11.99)	0.003*	3.96 (1.69 - 9.3)	0.011*	3.34 (1.56 - 7.17)	0.010*	1.86 (0.94 - 3.68)	0.122	2.43 (1.26 - 4.69)	0.033*	2.49 (1.31 - 4.72)	0.014*
lyso.PC.e.C18.0	0.76 (0.31 - 1.86)	0.650	1.43 (0.61 - 3.37)	0.617	1.41 (0.66 - 3.04)	0.507	0.92 (0.46 - 1.83)	0.878	1.56 (0.8 - 3.01)	0.321	1.3 (0.68 - 2.46)	0.492
lyso.PC.e.C18.1	4.29 (1.75 - 10.52)	0.008*	2.23 (0.95 - 5.25)	0.183	2.69 (1.26 - 5.76)	0.033*	2.39 (1.21 - 4.74)	0.031*	2.73 (1.41 - 5.27)	0.014*	3 (1.59 - 5.66)	0.003*
PC.aa.C30.0	6.36 (2.6 - 15.55)	<0.001*	2.01 (0.85 - 4.75)	0.258	1.94 (0.91 - 4.17)	0.164	2.02 (1.02 - 4)	0.079	1.25 (0.64 - 2.43)	0.624	1.43 (0.75 - 2.71)	0.343
PC.aa.C30.3	2.15 (0.88 - 5.27)	0.167	2.57 (1.1 - 6.04)	0.110	1.81 (0.84 - 3.91)	0.230	2.82 (1.43 - 5.56)	0.008*	2.06 (1.07 - 3.97)	0.081	1.99 (1.05 - 3.79)	0.066
PC.aa.C32.0	7.1 (2.92 - 17.29)	<0.001*	3 (1.28 - 7.04)	0.056	2.8 (1.3 - 6.02)	0.029*	4.7 (2.39 - 9.25)	<0.001*	2.77 (1.44 - 5.34)	0.013*	3.4 (1.8 - 6.42)	0.001*
PC.aa.C32.1	14.68 (6.09 - 35.36)	<0.001*	5.41 (2.31 - 12.69)	0.001*	5.93 (2.79 - 12.6)	<0.001*	5.03 (2.56 - 9.89)	<0.001*	4.1 (2.13 - 7.9)	<0.001*	4.39 (2.33 - 8.24)	<0.001*

PC.aa.C32.2	8.35 (3.43 - 20.33)	<0.001*	2.96 (1.26 - 6.94)	0.059	3.54 (1.66 - 7.57)	0.006*	2.38 (1.2 - 4.72)	0.031*	2.14 (1.11 - 4.14)	0.065	2.04 (1.08 - 3.86)	0.056
PC.aa.C32.3	2.85 (1.17 - 6.98)	0.059	1.98 (0.84 - 4.67)	0.260	1.91 (0.89 - 4.11)	0.175	3.17 (1.61 - 6.25)	0.004*	2.18 (1.13 - 4.21)	0.059	2.79 (1.48 - 5.27)	0.005*
PC.aa.C34.1	6.47 (2.65 - 15.81)	<0.001*	3.78 (1.61 - 8.87)	0.015*	4.34 (2.02 - 9.29)	0.002*	4.22 (2.14 - 8.32)	<0.001*	3.08 (1.6 - 5.94)	0.005*	3.96 (2.1 - 7.49)	<0.001*
PC.aa.C34.2	3.92 (1.59 - 9.66)	0.012*	2 (0.85 - 4.7)	0.258	2.25 (1.04 - 4.84)	0.088	2.36 (1.19 - 4.69)	0.033*	1.63 (0.84 - 3.16)	0.273	2.01 (1.06 - 3.82)	0.064
PC.aa.C34.3	6.59 (2.7 - 16.08)	<0.001*	2.43 (1.03 - 5.73)	0.136	2.97 (1.38 - 6.38)	0.020*	2.77 (1.4 - 5.48)	0.010*	2.29 (1.18 - 4.42)	0.047*	2.65 (1.4 - 5.02)	0.009*
PC.aa.C34.4	16.16 (6.73 - 38.81)	<0.001*	4.95 (2.11 - 11.62)	0.003*	4.92 (2.3 - 10.5)	0.001*	4.61 (2.35 - 9.05)	<0.001*	3.42 (1.77 - 6.59)	0.002*	3.1 (1.64 - 5.86)	0.002*
PC.aa.C34.5	3.95 (1.62 - 9.64)	0.011*	1.42 (0.6 - 3.36)	0.617	1.77 (0.83 - 3.8)	0.234	1.9 (0.96 - 3.74)	0.110	1.2 (0.62 - 2.33)	0.699	1.61 (0.85 - 3.04)	0.199
PC.aa.C36.0	1.41 (0.57 - 3.45)	0.563	1.51 (0.64 - 3.55)	0.540	1.37 (0.64 - 2.94)	0.558	1.78 (0.9 - 3.52)	0.147	1.52 (0.79 - 2.95)	0.343	1.56 (0.82 - 2.95)	0.233
PC.aa.C36.1	7.43 (3.06 - 18.05)	<0.001*	2.79 (1.18 - 6.58)	0.079	3.45 (1.61 - 7.41)	0.007*	3.31 (1.68 - 6.53)	0.002*	1.73 (0.89 - 3.36)	0.214	3.04 (1.6 - 5.74)	0.003*
PC.aa.C36.2	3.64 (1.49 - 8.9)	0.017*	1.75 (0.74 - 4.13)	0.363	2.03 (0.95 - 4.36)	0.137	2.06 (1.04 - 4.09)	0.070	1.29 (0.67 - 2.51)	0.571	1.65 (0.87 - 3.12)	0.179
PC.aa.C36.3	8 (3.29 - 19.49)	<0.001*	4.08 (1.74 - 9.58)	0.010*	5.04 (2.36 - 10.76)	<0.001*	3.7 (1.88 - 7.31)	0.001*	3.15 (1.63 - 6.08)	0.004*	3.11 (1.64 - 5.87)	0.002*
PC.aa.C36.4	9.43 (3.9 - 22.82)	<0.001*	5.98 (2.56 - 13.97)	0.001*	5.33 (2.49 - 11.43)	<0.001*	6.77 (3.46 - 13.22)	<0.001*	5.33 (2.78 - 10.2)	<0.001*	4.76 (2.52 - 8.98)	<0.001*
PC.aa.C36.5	5.95 (2.45 - 14.48)	0.001*	1.62 (0.69 - 3.83)	0.451	3.28 (1.54 - 7.02)	0.010*	2.3 (1.17 - 4.54)	0.036*	1.74 (0.9 - 3.38)	0.210	2.82 (1.49 - 5.32)	0.005*
PC.aa.C36.6	5.61 (2.3 - 13.69)	0.001*	1.98 (0.84 - 4.68)	0.260	3.52 (1.65 - 7.51)	0.007*	1.88 (0.95 - 3.71)	0.116	1.69 (0.87 - 3.27)	0.243	2.39 (1.26 - 4.51)	0.020*
PC.aa.C38.0	1.66 (0.68 - 4.06)	0.383	1.04 (0.44 - 2.47)	0.975	1.52 (0.71 - 3.26)	0.410	1.86 (0.94 - 3.68)	0.120	1.29 (0.66 - 2.51)	0.573	2.2 (1.16 - 4.17)	0.033*
PC.aa.C38.2	5.2 (2.13 - 12.69)	0.002*	3.13 (1.33 - 7.36)	0.045*	3.56 (1.67 - 7.62)	0.006*	2.46 (1.24 - 4.86)	0.024*	2.56 (1.33 - 4.95)	0.022*	2.59 (1.37 - 4.89)	0.010*
PC.aa.C38.3	12.46 (5.17 - 30.03)	<0.001*	8.75 (3.75 - 20.39)	<0.001*	9.78 (4.62 - 20.67)	<0.001*	5.54 (2.83 - 10.84)	<0.001*	4.96 (2.58 - 9.53)	<0.001*	5.1 (2.71 - 9.57)	<0.001*
PC.aa.C38.4	7.18 (2.96 - 17.43)	<0.001*	6.75 (2.89 - 15.73)	<0.001*	6.01 (2.81 - 12.84)	<0.001*	7.06 (3.62 - 13.79)	<0.001*	5.09 (2.66 - 9.76)	<0.001*	4.94 (2.62 - 9.32)	<0.001*
PC.aa.C38.5	3.7 (1.52 - 9.03)	0.016*	3.09 (1.32 - 7.24)	0.048*	4.32 (2.02 - 9.26)	0.002*	3.34 (1.69 - 6.57)	0.002*	2.81 (1.46 - 5.42)	0.011*	3.3 (1.74 - 6.23)	0.001*

PC.aa.C38.6	1.73 (0.7 - 4.24)	0.353	1.41 (0.6 - 3.32)	0.624	2.19 (1.02 - 4.71)	0.096	2.22 (1.12 - 4.38)	0.043*	1.83 (0.94 - 3.53)	0.168	2.3 (1.21 - 4.37)	0.025*
PC.aa.C40.0	2.22 (0.91 - 5.44)	0.148	1.17 (0.49 - 2.76)	0.848	1.43 (0.67 - 3.07)	0.496	2.2 (1.11 - 4.34)	0.046*	1.26 (0.65 - 2.45)	0.612	1.61 (0.85 - 3.05)	0.199
PC.aa.C40.1	1.87 (0.76 - 4.58)	0.282	2.07 (0.87 - 4.88)	0.242	1.36 (0.63 - 2.92)	0.558	2.3 (1.16 - 4.55)	0.036	1.53 (0.79 - 2.97)	0.338	1.21 (0.64 - 2.3)	0.617
PC.aa.C40.2	2.87 (1.17 - 7.04)	0.059	1.06 (0.45 - 2.49)	0.962	1.95 (0.91 - 4.19)	0.163	2.15 (1.08 - 4.25)	0.054	1.09 (0.56 - 2.1)	0.873	1.35 (0.71 - 2.57)	0.416
PC.aa.C40.3	3.63 (1.49 - 8.87)	0.017*	1.93 (0.82 - 4.55)	0.272	2.47 (1.15 - 5.32)	0.051	2.85 (1.44 - 5.61)	0.008*	2.21 (1.14 - 4.27)	0.056	1.67 (0.88 - 3.16)	0.171
PC.aa.C40.4	8.37 (3.45 - 20.29)	<0.001*	6.82 (2.93 - 15.91)	<0.001*	6.91 (3.26 - 14.65)	<0.001*	6.63 (3.39 - 12.96)	<0.001*	4.93 (2.57 - 9.45)	<0.001*	4.03 (2.14 - 7.57)	<0.001*
PC.aa.C40.5	2.78 (1.14 - 6.8)	0.064	3.39 (1.45 - 7.96)	0.030*	3.8 (1.77 - 8.13)	0.004*	2.98 (1.51 - 5.86)	0.005*	2.32 (1.2 - 4.47)	0.042*	2.35 (1.24 - 4.46)	0.022*
PC.aa.C40.6	3.73 (1.53 - 9.09)	0.015*	2.39 (1.01 - 5.61)	0.148	3.68 (1.72 - 7.86)	0.005*	3.83 (1.95 - 7.54)	0.001*	2.4 (1.25 - 4.63)	0.035*	3.34 (1.77 - 6.31)	0.001*
PC.aa.C42.0	1.58 (0.64 - 3.88)	0.441	1.2 (0.51 - 2.84)	0.821	2.11 (0.99 - 4.51)	0.111	1.85 (0.94 - 3.67)	0.121	1.31 (0.67 - 2.55)	0.559	1.81 (0.96 - 3.41)	0.109
PC.aa.C42.5	1.15 (0.46 - 2.85)	0.824	0.81 (0.34 - 1.91)	0.781	1.14 (0.53 - 2.45)	0.812	1.62 (0.81 - 3.23)	0.238	1.12 (0.58 - 2.16)	0.837	1.67 (0.88 - 3.15)	0.169
PC.aa.C43.6	1.22 (0.5 - 3)	0.750	1.46 (0.62 - 3.44)	0.588	1.71 (0.8 - 3.68)	0.265	1.61 (0.81 - 3.19)	0.238	1.58 (0.81 - 3.06)	0.308	1.74 (0.92 - 3.29)	0.136
PC.aa.C44.12	2.47 (1.01 - 6.05)	0.099	2.37 (1.01 - 5.59)	0.148	3.06 (1.42 - 6.6)	0.017*	2.89 (1.46 - 5.69)	0.007*	2.25 (1.16 - 4.35)	0.052	2.83 (1.49 - 5.36)	0.005*
PC.ae.C30.0	3.6 (1.47 - 8.81)	0.018*	1.83 (0.78 - 4.3)	0.319	1.76 (0.82 - 3.77)	0.239	1.73 (0.88 - 3.43)	0.171	1.21 (0.62 - 2.34)	0.697	1.12 (0.59 - 2.12)	0.769
PC.ae.C32.0	3.59 (1.47 - 8.8)	0.018*	2.3 (0.98 - 5.41)	0.162	2.41 (1.13 - 5.18)	0.059	2.4 (1.21 - 4.75)	0.030*	1.66 (0.86 - 3.22)	0.256	1.91 (1.01 - 3.62)	0.080
PC.ae.C32.1	2.57 (1.05 - 6.28)	0.086	2.24 (0.95 - 5.27)	0.182	1.42 (0.66 - 3.07)	0.507	2.32 (1.18 - 4.59)	0.034*	1.67 (0.86 - 3.23)	0.253	1.6 (0.84 - 3.04)	0.209
PC.ae.C32.2	1.36 (0.55 - 3.33)	0.606	1.02 (0.43 - 2.4)	0.975	0.79 (0.37 - 1.7)	0.671	1.43 (0.72 - 2.84)	0.396	0.95 (0.49 - 1.83)	0.912	1.12 (0.59 - 2.12)	0.769
PC.ae.C34.0	2.4 (0.98 - 5.88)	0.114	1.89 (0.8 - 4.44)	0.284	1.61 (0.75 - 3.46)	0.333	1.72 (0.87 - 3.41)	0.178	1.3 (0.67 - 2.52)	0.566	1.17 (0.62 - 2.22)	0.688
PC.ae.C34.1	3.1 (1.26 - 7.61)	0.040*	1.91 (0.81 - 4.5)	0.278	2.24 (1.04 - 4.83)	0.088	2.06 (1.04 - 4.08)	0.071	1.46 (0.76 - 2.83)	0.399	1.85 (0.97 - 3.51)	0.098
PC.ae.C34.2	1.42 (0.58 - 3.5)	0.563	1.03 (0.43 - 2.43)	0.975	1.2 (0.56 - 2.59)	0.742	1.27 (0.64 - 2.52)	0.587	0.88 (0.45 - 1.71)	0.804	1.12 (0.59 - 2.13)	0.769

PC.ae.C34.3	1.18 (0.48 - 2.91)	0.790	0.97 (0.41 - 2.29)	0.975	0.72 (0.34 - 1.56)	0.544	1.16 (0.59 - 2.3)	0.755	0.72 (0.37 - 1.39)	0.464	0.94 (0.49 - 1.78)	0.863
PC.ae.C34.4	1.21 (0.49 - 2.97)	0.765	0.79 (0.34 - 1.87)	0.770	1.13 (0.52 - 2.42)	0.833	0.99 (0.5 - 1.95)	0.982	0.76 (0.39 - 1.46)	0.548	1.11 (0.59 - 2.11)	0.778
PC.ae.C36.0	1.12 (0.46 - 2.75)	0.847	1.24 (0.53 - 2.91)	0.781	1.24 (0.58 - 2.67)	0.691	1.91 (0.96 - 3.78)	0.108	1.55 (0.8 - 3)	0.321	1.84 (0.97 - 3.48)	0.100
PC.ae.C36.1	1.96 (0.8 - 4.83)	0.238	1.86 (0.79 - 4.4)	0.299	2.54 (1.19 - 5.43)	0.044	2.26 (1.14 - 4.47)	0.041*	1.62 (0.83 - 3.13)	0.283	1.99 (1.05 - 3.77)	0.064
PC.ae.C36.2	0.93 (0.38 - 2.3)	0.904	0.75 (0.32 - 1.77)	0.706	1.09 (0.51 - 2.34)	0.883	0.93 (0.47 - 1.85)	0.899	0.74 (0.38 - 1.43)	0.515	0.8 (0.42 - 1.52)	0.555
PC.ae.C36.3	2.6 (1.06 - 6.38)	0.082	1.75 (0.74 - 4.15)	0.363	2.57 (1.19 - 5.53)	0.044*	1.93 (0.97 - 3.81)	0.101	1.55 (0.8 - 3)	0.334	2.11 (1.11 - 4.01)	0.047*
PC.ae.C36.4	4.89 (2.01 - 11.91)	0.003*	5.38 (2.29 - 12.6)	0.001*	4.32 (2.02 - 9.24)	0.002*	4.72 (2.41 - 9.26)	<0.001*	3.81 (1.98 - 7.35)	0.001*	3.59 (1.9 - 6.78)	0.001*
PC.ae.C36.5	3.32 (1.36 - 8.11)	0.027*	2.58 (1.09 - 6.08)	0.110	2.35 (1.09 - 5.05)	0.067	3.75 (1.91 - 7.38)	0.001*	2.63 (1.36 - 5.08)	0.019*	2.94 (1.55 - 5.56)	0.004*
PC.ae.C38.0	1.88 (0.77 - 4.61)	0.273	1.02 (0.43 - 2.4)	0.975	1.76 (0.82 - 3.79)	0.240	1.65 (0.84 - 3.27)	0.211	1.54 (0.79 - 2.97)	0.337	1.9 (1 - 3.61)	0.083
PC.ae.C38.2	2.09 (0.85 - 5.14)	0.188	1.75 (0.74 - 4.12)	0.363	1.35 (0.63 - 2.89)	0.563	1.25 (0.63 - 2.48)	0.618	0.99 (0.51 - 1.91)	0.987	0.95 (0.5 - 1.8)	0.897
PC.ae.C38.3	4.14 (1.7 - 10.11)	0.009*	3.82 (1.62 - 8.97)	0.015*	4.73 (2.21 - 10.12)	0.001*	2.94 (1.49 - 5.8)	0.006*	2.82 (1.46 - 5.44)	0.012*	2.38 (1.25 - 4.52)	0.021*
PC.ae.C38.4	2.9 (1.19 - 7.1)	0.055	3.29 (1.4 - 7.72)	0.035*	2.91 (1.35 - 6.27)	0.023*	2.98 (1.51 - 5.87)	0.005*	2.24 (1.16 - 4.32)	0.052	2.22 (1.17 - 4.23)	0.033*
PC.ae.C38.5	2.7 (1.11 - 6.61)	0.072	2.62 (1.11 - 6.17)	0.104	2.67 (1.24 - 5.74)	0.036*	3.35 (1.7 - 6.59)	0.002*	2.63 (1.36 - 5.08)	0.019*	3.1 (1.64 - 5.87)	0.002*
PC.ae.C38.6	2.46 (1 - 6.01)	0.100	1.77 (0.75 - 4.19)	0.356	2.4 (1.12 - 5.17)	0.060	2.93 (1.49 - 5.77)	0.006*	2.01 (1.04 - 3.9)	0.097	2.85 (1.51 - 5.41)	0.005*
PC.ae.C40.0	2.47 (1.01 - 6.05)	0.099	1.3 (0.55 - 3.07)	0.731	2.17 (1.01 - 4.67)	0.100	2.73 (1.38 - 5.38)	0.011*	2.17 (1.13 - 4.2)	0.060	1.86 (0.98 - 3.54)	0.093
PC.ae.C40.1	2.65 (1.08 - 6.48)	0.077	1.25 (0.53 - 2.96)	0.779	2.07 (0.96 - 4.44)	0.124	1.87 (0.95 - 3.7)	0.116	1.26 (0.65 - 2.44)	0.617	1.59 (0.84 - 3.02)	0.209
PC.ae.C40.2	1.13 (0.46 - 2.77)	0.847	0.71 (0.3 - 1.68)	0.624	1.81 (0.84 - 3.87)	0.226	1.28 (0.65 - 2.55)	0.566	0.92 (0.47 - 1.8)	0.873	1.34 (0.71 - 2.54)	0.427
PC.ae.C40.3	2.8 (1.14 - 6.84)	0.063	2.38 (1.01 - 5.6)	0.148	2.41 (1.12 - 5.17)	0.060	2.37 (1.2 - 4.68)	0.031*	1.64 (0.85 - 3.19)	0.268	1.42 (0.75 - 2.7)	0.351
PC.ae.C40.4	2.61 (1.06 - 6.38)	0.082	3.33 (1.42 - 7.83)	0.034*	2.55 (1.18 - 5.5)	0.044*	2.22 (1.12 - 4.38)	0.043*	1.45 (0.75 - 2.81)	0.415	1.54 (0.81 - 2.93)	0.245

PC.ae.C40.5	1.49 (0.61 - 3.65)	0.510	1.7 (0.72 - 4)	0.396	2.22 (1.03 - 4.8)	0.093	2.27 (1.15 - 4.48)	0.039*	1.51 (0.78 - 2.92)	0.354	2.08 (1.09 - 3.97)	0.051
PC.ae.C40.6	1.06 (0.43 - 2.6)	0.924	0.98 (0.41 - 2.3)	0.975	1.69 (0.79 - 3.63)	0.281	1.47 (0.74 - 2.9)	0.367	1.12 (0.58 - 2.17)	0.827	1.66 (0.88 - 3.15)	0.171
PC.ae.C42.1	2.66 (1.09 - 6.51)	0.077	2.51 (1.06 - 5.9)	0.121	2.41 (1.13 - 5.17)	0.059	3.43 (1.74 - 6.76)	0.002*	3.61 (1.87 - 6.95)	0.001*	1.76 (0.93 - 3.34)	0.125
PC.ae.C42.3	0.89 (0.36 - 2.19)	0.847	0.57 (0.24 - 1.36)	0.368	1.28 (0.6 - 2.75)	0.655	0.96 (0.49 - 1.91)	0.961	0.57 (0.29 - 1.1)	0.206	1 (0.53 - 1.89)	0.995
PC.ae.C42.4	2.68 (1.09 - 6.56)	0.076	2.1 (0.89 - 4.96)	0.227	2.75 (1.28 - 5.9)	0.030*	1.71 (0.86 - 3.38)	0.180	1.48 (0.76 - 2.86)	0.381	1.64 (0.87 - 3.11)	0.182
PC.ae.C42.5	2.02 (0.82 - 4.95)	0.211	2.12 (0.9 - 5)	0.222	2.77 (1.29 - 5.97)	0.030*	2.29 (1.16 - 4.52)	0.036*	1.6 (0.83 - 3.09)	0.291	1.92 (1.01 - 3.66)	0.079
PC.ae.C42.6	1.42 (0.58 - 3.47)	0.563	1.23 (0.52 - 2.9)	0.795	1.7 (0.79 - 3.65)	0.275	1.54 (0.78 - 3.05)	0.293	1.15 (0.59 - 2.23)	0.793	1.62 (0.85 - 3.06)	0.199
SM.a.C30.1	4.05 (1.65 - 9.94)	0.010*	3 (1.27 - 7.06)	0.057	3.01 (1.41 - 6.44)	0.018*	3.33 (1.68 - 6.58)	0.002*	2.21 (1.14 - 4.27)	0.056	2.26 (1.2 - 4.27)	0.028*
SM.a.C32.1	3.41 (1.39 - 8.38)	0.024*	2.58 (1.1 - 6.06)	0.110	2.92 (1.36 - 6.27)	0.023*	3.1 (1.56 - 6.13)	0.005*	2.67 (1.38 - 5.14)	0.017*	2.16 (1.14 - 4.1)	0.040*
SM.a.C32.2	8.42 (3.46 - 20.46)	<0.001*	6.53 (2.8 - 15.2)	<0.001*	7.89 (3.72 - 16.74)	<0.001*	7.84 (4.01 - 15.33)	<0.001*	5.6 (2.93 - 10.7)	<0.001*	6.24 (3.33 - 11.69)	<0.001*
SM.a.C33.1	1.37 (0.55 - 3.39)	0.598	1.33 (0.57 - 3.13)	0.706	1.52 (0.71 - 3.27)	0.410	1.85 (0.93 - 3.69)	0.123	1.51 (0.78 - 2.91)	0.354	1.32 (0.69 - 2.51)	0.459
SM.a.C34.1	1.52 (0.62 - 3.73)	0.491	1.7 (0.72 - 4)	0.396	1.89 (0.87 - 4.09)	0.194	2.62 (1.32 - 5.17)	0.015*	1.9 (0.98 - 3.67)	0.135	1.77 (0.93 - 3.38)	0.126
SM.a.C34.2	5.45 (2.24 - 13.29)	0.001*	4.7 (2.01 - 10.98)	0.004*	5.17 (2.42 - 11.07)	<0.001*	8.17 (4.19 - 15.93)	<0.001*	4.86 (2.54 - 9.31)	<0.001*	5.14 (2.73 - 9.69)	<0.001*
SM.a.C35.0	1.03 (0.41 - 2.58)	0.962	1.1 (0.46 - 2.63)	0.913	2.03 (0.93 - 4.42)	0.146	2.18 (1.08 - 4.38)	0.055	1.64 (0.83 - 3.25)	0.283	2.35 (1.21 - 4.54)	0.026*
SM.a.C35.1	0.57 (0.23 - 1.4)	0.335	0.9 (0.38 - 2.13)	0.913	1 (0.46 - 2.15)	0.996	1.55 (0.78 - 3.08)	0.287	1.53 (0.79 - 2.96)	0.338	1.42 (0.74 - 2.69)	0.352
SM.a.C36.1	2.8 (1.14 - 6.86)	0.063	2.46 (1.05 - 5.79)	0.127	2.39 (1.11 - 5.15)	0.063	4.35 (2.21 - 8.57)	<0.001*	3.21 (1.67 - 6.18)	0.003*	4.22 (2.23 - 7.98)	<0.001*
SM.a.C36.2	2.48 (1.01 - 6.07)	0.099	2.64 (1.12 - 6.19)	0.102	2.7 (1.25 - 5.81)	0.035*	5.75 (2.94 - 11.25)	<0.001*	4.04 (2.1 - 7.76)	<0.001*	4.89 (2.59 - 9.23)	<0.001*
SM.a.C36.3	3.3 (1.35 - 8.07)	0.027*	2.2 (0.94 - 5.16)	0.191	2.5 (1.16 - 5.4)	0.050	5.01 (2.56 - 9.83)	<0.001*	3.66 (1.91 - 7.03)	0.001*	4.06 (2.14 - 7.67)	<0.001*
SM.a.C37.1	2.73 (1.11 - 6.73)	0.072	2.05 (0.87 - 4.84)	0.243	3.12 (1.46 - 6.65)	0.014*	2.23 (1.12 - 4.42)	0.043*	2.5 (1.3 - 4.83)	0.027*	3.06 (1.62 - 5.76)	0.002*

SM.a.C38.2	2.68 (1.08 - 6.62)	0.077	2.37 (1.01 - 5.57)	0.148	2.36 (1.1 - 5.08)	0.066	2.33 (1.17 - 4.64)	0.036*	2.07 (1.07 - 3.99)	0.081	2.73 (1.44 - 5.18)	0.007*
SM.a.C38.3	2.6 (1.05 - 6.41)	0.085	1.95 (0.83 - 4.59)	0.267	1.59 (0.74 - 3.43)	0.351	2.36 (1.19 - 4.68)	0.033*	2.11 (1.09 - 4.08)	0.070	2.26 (1.19 - 4.29)	0.028*
SM.a.C39.1	2.41 (0.97 - 5.95)	0.114	1.95 (0.83 - 4.59)	0.267	2.65 (1.24 - 5.67)	0.036*	2.38 (1.2 - 4.73)	0.031*	1.94 (1 - 3.74)	0.120	1.92 (1.02 - 3.63)	0.078
SM.a.C39.2	1.12 (0.45 - 2.75)	0.851	0.75 (0.32 - 1.77)	0.706	1.81 (0.84 - 3.87)	0.228	1.67 (0.84 - 3.32)	0.207	1.32 (0.68 - 2.55)	0.550	1.99 (1.05 - 3.77)	0.064
SM.a.C40.2	3.48 (1.42 - 8.54)	0.022*	2.83 (1.21 - 6.66)	0.074	3.29 (1.53 - 7.06)	0.010*	3.52 (1.78 - 6.95)	0.002*	2.37 (1.23 - 4.58)	0.037*	2.8 (1.48 - 5.31)	0.005*
SM.a.C40.5	5.5 (2.26 - 13.39)	0.001*	2.01 (0.85 - 4.74)	0.258	4.79 (2.26 - 10.19)	0.001*	3.02 (1.53 - 5.95)	0.005*	2.06 (1.07 - 4)	0.081	4.06 (2.16 - 7.62)	<0.001*
SM.a.C41.1	4.17 (1.69 - 10.29)	0.009*	2 (0.85 - 4.7)	0.258	2.62 (1.22 - 5.61)	0.038	3.14 (1.58 - 6.24)	0.004*	2.57 (1.33 - 4.95)	0.022*	3.01 (1.59 - 5.69)	0.003*
SM.a.C41.2	1.91 (0.77 - 4.71)	0.266	1.1 (0.47 - 2.59)	0.913	2.69 (1.26 - 5.76)	0.033*	2.38 (1.2 - 4.73)	0.031*	1.77 (0.92 - 3.42)	0.194	2.51 (1.33 - 4.74)	0.013*
SM.a.C42.1	2.28 (0.93 - 5.59)	0.135	1.42 (0.6 - 3.34)	0.617	2.07 (0.97 - 4.43)	0.122	2.83 (1.43 - 5.59)	0.008*	1.81 (0.94 - 3.5)	0.171	2.3 (1.22 - 4.34)	0.025*
SM.a.C42.2	2.47 (1.01 - 6.06)	0.099	1.34 (0.57 - 3.16)	0.704	2.87 (1.33 - 6.18)	0.026*	3.86 (1.96 - 7.6)	0.001*	2.04 (1.06 - 3.94)	0.086	3.35 (1.76 - 6.35)	0.001*
SM.a.C42.3	3.17 (1.3 - 7.74)	0.035*	2.52 (1.07 - 5.92)	0.120	3.67 (1.71 - 7.87)	0.006*	4.61 (2.35 - 9.05)	<0.001*	2.7 (1.4 - 5.2)	0.015*	3.68 (1.95 - 6.96)	<0.001*
SM.a.C42.4	5.51 (2.27 - 13.4)	0.001*	4.51 (1.93 - 10.55)	0.005*	4.31 (2 - 9.25)	0.002*	5.74 (2.93 - 11.23)	<0.001*	4.86 (2.54 - 9.32)	<0.001*	5.23 (2.77 - 9.87)	<0.001*
SM.a.C42.6	2.56 (1.04 - 6.28)	0.087	1.97 (0.84 - 4.64)	0.260	2.66 (1.23 - 5.72)	0.037*	2.88 (1.46 - 5.69)	0.007*	2.38 (1.23 - 4.58)	0.037*	2.82 (1.49 - 5.36)	0.005*
SM.a.C43.1	2.3 (0.94 - 5.65)	0.130	1.19 (0.5 - 2.8)	0.830	1.57 (0.73 - 3.36)	0.365	3.06 (1.55 - 6.04)	0.005*	1.52 (0.78 - 2.94)	0.348	2.25 (1.19 - 4.24)	0.029*
SM.a.C43.2	1.8 (0.73 - 4.41)	0.318	1.21 (0.51 - 2.86)	0.805	1.42 (0.66 - 3.04)	0.507	3.27 (1.66 - 6.46)	0.003*	2.33 (1.21 - 4.51)	0.042*	1.92 (1.01 - 3.64)	0.078
SM.a.C44.6	2.3 (0.94 - 5.64)	0.130	1.92 (0.82 - 4.52)	0.274	3.18 (1.49 - 6.8)	0.012*	3.06 (1.55 - 6.03)	0.005*	1.82 (0.94 - 3.52)	0.168	3.66 (1.95 - 6.89)	<0.001*
SM.e.C36.2	1.3 (0.53 - 3.2)	0.666	1.11 (0.47 - 2.61)	0.913	1.79 (0.83 - 3.87)	0.234	3.46 (1.76 - 6.82)	0.002*	2 (1.03 - 3.86)	0.097	2.37 (1.25 - 4.5)	0.022*
SM.e.C38.3	0.96 (0.39 - 2.37)	0.955	1.02 (0.43 - 2.41)	0.975	1.08 (0.5 - 2.32)	0.903	1.19 (0.6 - 2.36)	0.707	1.07 (0.55 - 2.08)	0.889	1.6 (0.85 - 3.04)	0.203
SM.e.C40.5	2.9 (1.19 - 7.09)	0.055	2.34 (0.99 - 5.53)	0.155	2.01 (0.94 - 4.32)	0.143	2.71 (1.38 - 5.35)	0.011*	2.18 (1.12 - 4.21)	0.060	3.13 (1.66 - 5.92)	0.002*

Carn	0.91 (0.36 - 2.26)	0.865	1.95 (0.82 - 4.59)	0.267	2.64 (1.24 - 5.66)	0.036	1.31 (0.65 - 2.62)	0.547	1.36 (0.7 - 2.63)	0.515	2.49 (1.32 - 4.71)	0.014
Carn.a.C10.0	0.41 (0.17 - 1)	0.100	0.83 (0.35 - 1.95)	0.811	1.12 (0.52 - 2.4)	0.840	2.24 (1.14 - 4.43)	0.041	1.6 (0.82 - 3.1)	0.291	1.91 (1.01 - 3.61)	0.080
Carn.a.C10.1	0.57 (0.23 - 1.39)	0.334	0.8 (0.34 - 1.9)	0.781	1.21 (0.56 - 2.61)	0.726	1.83 (0.92 - 3.61)	0.129	1.4 (0.72 - 2.72)	0.457	2.31 (1.22 - 4.37)	0.025*
Carn.a.C12.0	0.56 (0.23 - 1.38)	0.325	0.8 (0.34 - 1.88)	0.773	0.99 (0.46 - 2.13)	0.994	1.45 (0.73 - 2.87)	0.383	1.2 (0.62 - 2.33)	0.697	1.7 (0.9 - 3.23)	0.154
Carn.a.C14.1	0.5 (0.2 - 1.23)	0.220	0.65 (0.28 - 1.53)	0.510	1 (0.47 - 2.16)	0.996	1.3 (0.66 - 2.58)	0.547	1 (0.52 - 1.94)	0.998	1.78 (0.94 - 3.38)	0.120
Carn.a.C14.2	0.39 (0.16 - 0.97)	0.091	0.48 (0.2 - 1.13)	0.231	0.79 (0.37 - 1.71)	0.671	0.89 (0.45 - 1.77)	0.833	0.92 (0.47 - 1.77)	0.870	1.48 (0.78 - 2.8)	0.297
Carn.a.C15.0	0.73 (0.3 - 1.8)	0.598	1.07 (0.45 - 2.51)	0.954	1.72 (0.8 - 3.69)	0.263	0.99 (0.5 - 1.96)	0.983	1.06 (0.55 - 2.06)	0.899	1.35 (0.71 - 2.56)	0.417
Carn.a.C16.0	0.63 (0.26 - 1.54)	0.431	0.84 (0.36 - 1.99)	0.830	0.84 (0.39 - 1.8)	0.749	1.14 (0.57 - 2.25)	0.800	1.41 (0.73 - 2.74)	0.446	1.29 (0.68 - 2.45)	0.507
Carn.a.C16.0.Oxo	0.66 (0.27 - 1.62)	0.492	0.73 (0.31 - 1.71)	0.661	0.74 (0.34 - 1.6)	0.559	0.82 (0.41 - 1.62)	0.653	0.83 (0.43 - 1.6)	0.695	1.19 (0.62 - 2.26)	0.665
Carn.a.C16.1	0.35 (0.14 - 0.86)	0.059	0.57 (0.24 - 1.33)	0.356	1.09 (0.51 - 2.35)	0.883	0.9 (0.45 - 1.78)	0.844	1 (0.52 - 1.93)	0.998	1.67 (0.88 - 3.17)	0.171
Carn.a.C16.2	0.43 (0.17 - 1.05)	0.125	0.55 (0.23 - 1.29)	0.319	1.04 (0.48 - 2.23)	0.952	0.59 (0.3 - 1.17)	0.191	0.77 (0.4 - 1.49)	0.571	1.23 (0.65 - 2.33)	0.594
Carn.a.C18.0	0.99 (0.4 - 2.44)	0.987	0.46 (0.2 - 1.09)	0.207	0.97 (0.45 - 2.08)	0.952	0.76 (0.38 - 1.51)	0.537	0.62 (0.32 - 1.2)	0.286	1.02 (0.54 - 1.93)	0.958
Carn.a.C18.1	0.56 (0.23 - 1.37)	0.318	0.77 (0.33 - 1.81)	0.731	1.79 (0.83 - 3.84)	0.232	1.32 (0.67 - 2.61)	0.533	1.27 (0.65 - 2.45)	0.610	2.32 (1.23 - 4.38)	0.024
Carn.a.C18.2	0.6 (0.24 - 1.46)	0.375	1.02 (0.43 - 2.42)	0.975	1.17 (0.54 - 2.51)	0.783	1.02 (0.52 - 2.02)	0.978	1.14 (0.59 - 2.22)	0.793	1.71 (0.9 - 3.24)	0.150
Carn.a.C18.2.OH	0.7 (0.28 - 1.72)	0.563	0.77 (0.33 - 1.82)	0.731	1.12 (0.52 - 2.42)	0.840	0.8 (0.4 - 1.59)	0.620	0.84 (0.44 - 1.63)	0.721	1.04 (0.54 - 1.98)	0.924
Carn.a.C2.0	1.41 (0.58 - 3.47)	0.563	1.61 (0.68 - 3.78)	0.455	1.92 (0.89 - 4.14)	0.174	2.22 (1.12 - 4.4)	0.043*	1.81 (0.94 - 3.5)	0.171	3.21 (1.7 - 6.07)	0.002*
Carn.a.C20.0	1.29 (0.52 - 3.18)	0.680	1.45 (0.62 - 3.42)	0.588	0.84 (0.39 - 1.81)	0.757	1.41 (0.71 - 2.81)	0.421	1.65 (0.86 - 3.19)	0.259	1.19 (0.63 - 2.25)	0.659
Carn.a.C20.1	1.23 (0.5 - 3.03)	0.745	1.11 (0.47 - 2.61)	0.913	1.36 (0.63 - 2.92)	0.558	1.02 (0.52 - 2.03)	0.978	0.94 (0.49 - 1.82)	0.899	1.4 (0.74 - 2.66)	0.362
Carn.a.C20.3	0.61 (0.25 - 1.5)	0.394	0.91 (0.39 - 2.15)	0.913	0.62 (0.29 - 1.33)	0.331	0.77 (0.39 - 1.52)	0.549	0.88 (0.45 - 1.7)	0.803	0.93 (0.49 - 1.78)	0.863

Carn.a.C20.4	0.54 (0.22 - 1.33)	0.289	0.86 (0.36 - 2.02)	0.847	0.85 (0.4 - 1.84)	0.778	1.01 (0.51 - 2)	0.989	0.97 (0.5 - 1.89)	0.973	1.12 (0.59 - 2.14)	0.769
Carn.a.C3.0	2.31 (0.94 - 5.68)	0.130	2.06 (0.88 - 4.84)	0.242	2.14 (1 - 4.6)	0.105	1.72 (0.87 - 3.41)	0.177	1.44 (0.75 - 2.79)	0.420	2.15 (1.14 - 4.08)	0.040*
Carn.a.C3.0.DC	0.71 (0.29 - 1.74)	0.563	0.96 (0.41 - 2.26)	0.975	1.39 (0.64 - 2.98)	0.541	1.1 (0.56 - 2.18)	0.849	0.9 (0.46 - 1.74)	0.837	1.28 (0.67 - 2.42)	0.519
Carn.a.C4.0	2.36 (0.96 - 5.78)	0.118	1.89 (0.8 - 4.46)	0.285	1.57 (0.73 - 3.37)	0.365	1.39 (0.7 - 2.75)	0.444	1.18 (0.61 - 2.3)	0.728	1.59 (0.84 - 3.01)	0.209
Carn.a.C5.0	2.73 (1.11 - 6.67)	0.071	2.32 (0.99 - 5.45)	0.160	2.74 (1.28 - 5.88)	0.031	2.23 (1.13 - 4.41)	0.043	1.81 (0.94 - 3.5)	0.171	2.55 (1.35 - 4.83)	0.012*
Carn.a.C6.0	0.84 (0.34 - 2.06)	0.785	1 (0.42 - 2.36)	0.999	1.24 (0.58 - 2.67)	0.691	2.03 (1.03 - 4)	0.075	1.42 (0.73 - 2.75)	0.446	1.77 (0.93 - 3.35)	0.123
Carn.a.C6.0.OH	0.45 (0.18 - 1.1)	0.148	0.76 (0.32 - 1.78)	0.713	1.23 (0.57 - 2.64)	0.707	0.91 (0.46 - 1.81)	0.865	0.86 (0.45 - 1.67)	0.770	1.02 (0.54 - 1.94)	0.958
Carn.a.C8.0	0.44 (0.18 - 1.08)	0.138	0.76 (0.32 - 1.79)	0.721	0.97 (0.45 - 2.08)	0.952	1.77 (0.89 - 3.49)	0.155	1.33 (0.69 - 2.58)	0.538	1.43 (0.75 - 2.72)	0.341
Carn.a.C8.1	1.45 (0.59 - 3.57)	0.542	1.95 (0.83 - 4.57)	0.267	1.49 (0.7 - 3.2)	0.430	2.66 (1.35 - 5.26)	0.013	2.89 (1.5 - 5.58)	0.009*	2.99 (1.59 - 5.63)	0.003*
Carn.a.C9.0	1.03 (0.42 - 2.53)	0.962	1.14 (0.49 - 2.7)	0.867	1.46 (0.68 - 3.15)	0.458	1.44 (0.73 - 2.84)	0.395	1.1 (0.57 - 2.12)	0.859	1.14 (0.6 - 2.16)	0.744
Asn/Asp	0.23 (0.09 - 0.57)	0.008*	0.15 (0.07 - 0.36)	<0.001*	0.27 (0.13 - 0.57)	0.005*	0.12 (0.06 - 0.23)	<0.001*	0.18 (0.09 - 0.34)	<0.001*	0.16 (0.08 - 0.29)	<0.001*
Gln/Glu	0.33 (0.13 - 0.8)	0.043*	0.16 (0.07 - 0.38)	<0.001*	0.16 (0.08 - 0.34)	<0.001*	0.25 (0.13 - 0.5)	0.001*	0.2 (0.1 - 0.38)	<0.001*	0.13 (0.07 - 0.24)	<0.001*
NEFA18.1/ NEFA18.0	0.8 (0.33 - 1.97)	0.733	0.7 (0.3 - 1.65)	0.617	0.97 (0.45 - 2.07)	0.953	1.08 (0.55 - 2.15)	0.878	0.9 (0.46 - 1.77)	0.845	1.48 (0.78 - 2.81)	0.297
NEFA16.1/ NEFA16.0	0.84 (0.34 - 2.07)	0.789	0.78 (0.33 - 1.82)	0.741	1.31 (0.61 - 2.79)	0.610	1.67 (0.84 - 3.31)	0.207	1.41 (0.72 - 2.75)	0.455	1.49 (0.78 - 2.83)	0.294
PC.aa/PC.ae	12.01 (4.95 - 29.13)	<0.001*	4.52 (1.93 - 10.6)	0.005*	4.14 (1.95 - 8.8)	0.002*	3.74 (1.89 - 7.38)	0.001*	3.32 (1.7 - 6.49)	0.003*	3.58 (1.89 - 6.79)	0.001*
Lyso.PC.a/PC.aa	1.43 (0.56 - 3.65)	0.563	2.79 (1.19 - 6.56)	0.078	1.33 (0.62 - 2.86)	0.579	0.64 (0.31 - 1.31)	0.304	1.29 (0.66 - 2.54)	0.573	1.42 (0.74 - 2.72)	0.352
(lyso.PC.a.C16.0 + lyso.PC.a.C18.0)/ PC.aa	1.19 (0.47 - 3.02)	0.792	3.47 (1.48 - 8.15)	0.027*	1.65 (0.77 - 3.54)	0.310	0.75 (0.37 - 1.52)	0.531	1.92 (0.98 - 3.76)	0.136	2.05 (1.07 - 3.91)	0.059

(lyso.PC.a.C18.1 + lyso.PC.a.C18.2)	1.12 (0.45 - 2.82)	0.847	0.94 (0.4 - 2.21)	0.954	0.55 (0.26 - 1.18)	0.220	0.38 (0.19 - 0.76)	0.016*	0.4 (0.21 - 0.78)	0.030*	0.46 (0.24 - 0.88)	0.040*
Carn.a.C.16.0/ free Carn	0.7 (0.28 - 1.73)	0.563	0.6 (0.26 - 1.42)	0.421	0.48 (0.22 - 1.02)	0.113	1.02 (0.51 - 2.03)	0.982	1.22 (0.62 - 2.38)	0.692	0.79 (0.41 - 1.51)	0.536
Carn.a.C2.0/ Carn.a.C16.0	1.75 (0.71 - 4.34)	0.342	1.6 (0.68 - 3.77)	0.455	1.77 (0.83 - 3.79)	0.234	1.59 (0.8 - 3.17)	0.259	1.08 (0.55 - 2.12)	0.876	1.6 (0.84 - 3.04)	0.209

Values represent absolute differences in blood pressure (95% confidence interval) and corresponding p-values from linear regression models that reflect the difference in blood pressure (mmHg) per SDS increase in maternal early-pregnancy metabolite concentrations (μmol/L) or metabolite ratio. Model includes gestational age at time of measurement. AA amino acids, NEFA non-esterified fatty acids, PC.aa diacyl-phosphatidylcholines, PC.ae acyl-alkyl-phosphatidylcholines, lyso.PC.a acyl-lysophosphatidylcholines, lyso.PC.e alkyl-lysophosphatidylcholines, Carn.a acyl-carnitines, SM sphingomyelins.

[a] p-value corrected for multiple hypothesis testing using Benjamin-Hochberg FDR correction.

*****Statistically significant

Table S4. Associations of early-pregnancy individual metabolites with systolic blood pressure in early-, mid- and late pregnancy. Full model.

	Differences in systolic blood pressure in mmHg (95% confidence interval)					
**Metabolite**	**Early pregnancy** N = 803	**P-value**[a]	**Mid pregnancy** N = 793	**P-value**[a]	**Late pregnancy** N = 800	**P-value**[a]
Ala	3.12 (1.24 - 7.8)	0,081	2.02 (0.82 - 4.97)	0,362	2.49 (1.13 - 5.5)	0,135
Arg	5.08 (2.04 - 12.69)	0,007*	2.52 (1.02 - 6.25)	0,208	3.28 (1.49 - 7.23)	0,063
Asn	3.31 (1.32 - 8.3)	0,066	1.11 (0.45 - 2.73)	0,917	3.08 (1.4 - 6.77)	0,063
Asp	1.99 (0.79 - 5.04)	0,336	2.76 (1.12 - 6.8)	0,175	3.09 (1.4 - 6.84)	0,063
Cit	1.34 (0.5 - 3.54)	0,750	0.86 (0.33 - 2.22)	0,887	1.15 (0.5 - 2.67)	0,884
Gln	2.29 (0.92 - 5.68)	0,223	1.04 (0.43 - 2.52)	0,968	0.97 (0.44 - 2.13)	0,985
Glu	1.79 (0.71 - 4.52)	0,417	3.47 (1.41 - 8.51)	0,081	3.12 (1.41 - 6.9)	0,063
Gly	0.86 (0.35 - 2.14)	0,874	1 (0.41 - 2.42)	1,000	1.35 (0.62 - 2.97)	0,707
His	3.72 (1.46 - 9.5)	0,044*	1.59 (0.64 - 3.96)	0,560	3.12 (1.41 - 6.93)	0,063
Ile	3.22 (1.27 - 8.16)	0,077	1.32 (0.53 - 3.27)	0,755	1.61 (0.72 - 3.57)	0,498
Leu	2.88 (1.15 - 7.21)	0,110	1.32 (0.54 - 3.23)	0,751	2.12 (0.97 - 4.65)	0,222
Lys	0.81 (0.32 - 2.04)	0,810	1.39 (0.56 - 3.44)	0,700	2.99 (1.36 - 6.6)	0,068
Met	2.96 (1.19 - 7.41)	0,101	1.49 (0.6 - 3.66)	0,614	1.82 (0.83 - 4.02)	0,351
Orn	1.28 (0.5 - 3.25)	0,781	1.29 (0.53 - 3.17)	0,755	2.08 (0.94 - 4.59)	0,256
Phe	5.83 (2.38 - 14.27)	0,003*	2.51 (1.04 - 6.07)	0,208	2.4 (1.11 - 5.22)	0,135
Pro	2.39 (0.96 - 5.96)	0,193	1.88 (0.77 - 4.58)	0,385	1.56 (0.71 - 3.42)	0,498
Trp	7.77 (3.13 - 19.29)	0,001*	2.93 (1.2 - 7.17)	0,158	3.06 (1.4 - 6.68)	0,063
Ser	2.22 (0.89 - 5.52)	0,243	1.2 (0.5 - 2.9)	0,834	2.58 (1.18 - 5.64)	0,123
Thr	1.6 (0.62 - 4.12)	0,523	1.57 (0.62 - 3.95)	0,579	2.18 (0.98 - 4.87)	0,214
Tyr	5.55 (2.24 - 13.7)	0,004*	2.12 (0.88 - 5.15)	0,300	2.75 (1.26 - 5.98)	0,091
Val	5.6 (2.25 - 13.96)	0,004*	1.95 (0.8 - 4.74)	0,374	2.72 (1.24 - 5.96)	0,094
Cys	0.97 (0.38 - 2.51)	0,983	1.12 (0.44 - 2.81)	0,917	1.47 (0.65 - 3.33)	0,620
NEFA_14_0	1.59 (0.63 - 3.98)	0,520	1.46 (0.6 - 3.56)	0,628	0.85 (0.39 - 1.87)	0,873
NEFA_14_1	1.03 (0.41 - 2.61)	0,983	1.28 (0.52 - 3.14)	0,755	0.99 (0.45 - 2.21)	0,993
NEFA_15_0	1.68 (0.67 - 4.19)	0,463	1.58 (0.65 - 3.82)	0,555	0.68 (0.31 - 1.49)	0,596
NEFA_16_0	1.81 (0.72 - 4.57)	0,408	1.79 (0.73 - 4.38)	0,421	0.87 (0.39 - 1.94)	0,884
NEFA_16_1	0.93 (0.37 - 2.34)	0,933	1.4 (0.57 - 3.43)	0,693	1.01 (0.45 - 2.25)	0,993
NEFA_16_2	1.31 (0.52 - 3.33)	0,750	1.45 (0.59 - 3.57)	0,640	0.88 (0.4 - 1.97)	0,893
NEFA_17_0	1.48 (0.6 - 3.67)	0,593	1.39 (0.58 - 3.35)	0,693	0.92 (0.42 - 2.01)	0,933
NEFA_17_1	1.57 (0.63 - 3.92)	0,523	1.44 (0.6 - 3.49)	0,638	0.86 (0.39 - 1.9)	0,873
NEFA_17_2	1.73 (0.69 - 4.32)	0,439	1.1 (0.45 - 2.69)	0,923	0.82 (0.37 - 1.8)	0,864
NEFA_18_0	1.55 (0.61 - 3.93)	0,546	1.25 (0.51 - 3.07)	0,801	0.82 (0.37 - 1.81)	0,864
NEFA_18_1	1.36 (0.54 - 3.43)	0,703	1.37 (0.56 - 3.34)	0,707	1.07 (0.49 - 2.38)	0,944
NEFA_18_2	1.57 (0.63 - 3.91)	0,523	1.12 (0.46 - 2.72)	0,910	0.71 (0.32 - 1.56)	0,656
NEFA_18_3	1.9 (0.76 - 4.73)	0,356	1.37 (0.57 - 3.32)	0,705	0.64 (0.29 - 1.41)	0,498
NEFA_19_1	1.24 (0.5 - 3.09)	0,804	1.07 (0.45 - 2.58)	0,960	0.82 (0.38 - 1.8)	0,864
NEFA_20_1	1.6 (0.63 - 4.02)	0,520	1.15 (0.47 - 2.81)	0,889	1.16 (0.52 - 2.57)	0,873
NEFA_20_2	1.26 (0.5 - 3.17)	0,784	1.3 (0.53 - 3.17)	0,755	1.18 (0.53 - 2.61)	0,873
NEFA_20_3	2.69 (1.04 - 6.98)	0,166	3.08 (1.23 - 7.7)	0,149	1.77 (0.78 - 4.01)	0,399
NEFA_20_4	2.19 (0.87 - 5.47)	0,257	1.99 (0.82 - 4.85)	0,363	1.71 (0.78 - 3.76)	0,417

NEFA_20_5	2.35 (0.93 - 5.94)	0,213	1.49 (0.61 - 3.67)	0,605	1.65 (0.75 - 3.64)	0,458
NEFA_22_3	1.48 (0.59 - 3.73)	0,602	2.6 (1.06 - 6.35)	0,207	2.44 (1.1 - 5.43)	0,142
NEFA_22_4	2.5 (0.97 - 6.43)	0,191	2.85 (1.15 - 7.09)	0,174	2 (0.88 - 4.5)	0,306
NEFA_22_5	2.2 (0.87 - 5.55)	0,257	1.81 (0.74 - 4.44)	0,416	1.58 (0.71 - 3.52)	0,498
NEFA_22_6	1.77 (0.7 - 4.48)	0,422	1.29 (0.52 - 3.19)	0,755	1.27 (0.57 - 2.83)	0,816
NEFA_24_0	2.36 (0.96 - 5.82)	0,193	2.08 (0.86 - 5.03)	0,320	0.9 (0.41 - 1.95)	0,904
NEFA_24_1	1.1 (0.44 - 2.73)	0,914	0.75 (0.31 - 1.83)	0,745	0.99 (0.45 - 2.18)	0,993
NEFA_24_2	1.01 (0.39 - 2.58)	0,990	1.46 (0.59 - 3.59)	0,638	1.43 (0.64 - 3.19)	0,640
NEFA_24_4	3.18 (1.27 - 7.96)	0,077	2.83 (1.17 - 6.86)	0,169	2.58 (1.17 - 5.66)	0,126
NEFA_24_5	3.49 (1.38 - 8.8)	0,052	2.48 (1.01 - 6.07)	0,208	3.72 (1.69 - 8.21)	0,030*
NEFA_26_0	3.71 (1.5 - 9.18)	0,041*	1.81 (0.75 - 4.37)	0,416	1.64 (0.75 - 3.57)	0,458
NEFA_26_1	2.02 (0.8 - 5.09)	0,320	2.53 (1.03 - 6.2)	0,208	1.88 (0.85 - 4.16)	0,325
NEFA_26_2	1.43 (0.55 - 3.71)	0,651	1.76 (0.71 - 4.38)	0,440	1.4 (0.63 - 3.14)	0,660
lyso.PC.a.C14.0	14.36 (5.71 - 36.13)	0,000*	8.73 (3.51 - 21.7)	0,001*	5.74 (2.57 - 12.83)	0,005*
lyso.PC.a.C16.0	3.67 (1.43 - 9.45)	0,049*	4.91 (1.97 - 12.28)	0,028*	2.64 (1.17 - 5.96)	0,127
lyso.PC.a.C16.1	5.19 (1.97 - 13.68)	0,011*	6.23 (2.46 - 15.82)	0,009*	4.9 (2.15 - 11.16)	0,011*
lyso.PC.a.C18.0	2.01 (0.78 - 5.18)	0,342	3.84 (1.54 - 9.54)	0,072	1.8 (0.81 - 4.04)	0,373
lyso.PC.a.C18.1	5.45 (2.18 - 13.63)	0,005*	3 (1.22 - 7.37)	0,149	1.93 (0.87 - 4.28)	0,306
lyso.PC.a.C18.2	5.44 (2.17 - 13.66)	0,005*	2.17 (0.89 - 5.28)	0,288	1.37 (0.62 - 3.02)	0,690
lyso.PC.a.C18.3	5.96 (2.45 - 14.48)	0,003*	2.16 (0.9 - 5.18)	0,288	2.86 (1.33 - 6.19)	0,073
lyso.PC.a.C20.3	8.33 (3.34 - 20.78)	0,000*	6.94 (2.84 - 16.96)	0,002*	4.94 (2.25 - 10.88)	0,008*
lyso.PC.a.C20.4	5.81 (2.31 - 14.62)	0,004*	4.87 (2 - 11.88)	0,027*	2.48 (1.12 - 5.47)	0,135
lyso.PC.a.C20.5	2.61 (1.03 - 6.6)	0,167	1.3 (0.53 - 3.14)	0,755	2.3 (1.05 - 5.02)	0,163
lyso.PC.a.C22.6	2.83 (1.12 - 7.14)	0,118	1.64 (0.67 - 4.01)	0,514	1.24 (0.56 - 2.73)	0,851
lyso.PC.e.C16.0	2.84 (1.12 - 7.21)	0,118	2.95 (1.19 - 7.28)	0,159	1.99 (0.89 - 4.44)	0,306
lyso.PC.e.C18.0	0.88 (0.35 - 2.22)	0,890	1.74 (0.71 - 4.24)	0,440	1.27 (0.58 - 2.8)	0,813
lyso.PC.e.C18.1	2.54 (0.98 - 6.63)	0,191	1.3 (0.51 - 3.29)	0,755	1.56 (0.69 - 3.55)	0,519
PC.aa.C30.0	6.15 (2.46 - 15.34)	0,003*	3.06 (1.25 - 7.52)	0,149	2.37 (1.07 - 5.22)	0,155
PC.aa.C30.3	1.16 (0.46 - 2.91)	0,879	1.82 (0.75 - 4.42)	0,416	0.87 (0.39 - 1.92)	0,876
PC.aa.C32.0	3.84 (1.51 - 9.78)	0,041*	2.63 (1.06 - 6.5)	0,207	1.79 (0.8 - 4.01)	0,378
PC.aa.C32.1	5.44 (2.13 - 13.89)	0,006*	3.82 (1.54 - 9.47)	0,072	3.94 (1.77 - 8.75)	0,023*
PC.aa.C32.2	6.56 (2.61 - 16.49)	0,003*	2.8 (1.14 - 6.87)	0,174	2.55 (1.15 - 5.63)	0,129
PC.aa.C32.3	1.42 (0.54 - 3.71)	0,668	1.8 (0.72 - 4.54)	0,428	1.17 (0.52 - 2.63)	0,873
PC.aa.C34.1	3.14 (1.24 - 7.97)	0,083	3.29 (1.35 - 8.04)	0,104	2.85 (1.29 - 6.31)	0,084
PC.aa.C34.2	2.31 (0.9 - 5.89)	0,237	1.9 (0.78 - 4.68)	0,385	1.48 (0.67 - 3.3)	0,596
PC.aa.C34.3	3.22 (1.27 - 8.2)	0,077	2 (0.81 - 4.94)	0,366	1.89 (0.85 - 4.21)	0,325
PC.aa.C34.4	7.95 (3.23 - 19.53)	0,000*	3.8 (1.57 - 9.19)	0,072	2.72 (1.24 - 5.97)	0,094
PC.aa.C34.5	4.64 (1.81 - 11.95)	0,016*	1.94 (0.77 - 4.88)	0,385	1.88 (0.84 - 4.23)	0,343
PC.aa.C36.0	1.21 (0.47 - 3.15)	0,836	1.7 (0.67 - 4.32)	0,498	1.18 (0.52 - 2.68)	0,873
PC.aa.C36.1	4.12 (1.65 - 10.3)	0,026*	2.4 (0.98 - 5.88)	0,233	2.28 (1.03 - 5.04)	0,170
PC.aa.C36.2	2.43 (0.95 - 6.22)	0,193	1.82 (0.74 - 4.51)	0,416	1.4 (0.63 - 3.12)	0,660
PC.aa.C36.3	3.26 (1.27 - 8.37)	0,077	2.75 (1.11 - 6.83)	0,179	2.57 (1.16 - 5.71)	0,129
PC.aa.C36.4	3.51 (1.39 - 8.84)	0,052	3.71 (1.51 - 9.1)	0,074	2.47 (1.11 - 5.49)	0,135

PC.aa.C36.5	4.65 (1.85 - 11.68)	0,013*	1.84 (0.74 - 4.54)	0,416	3.07 (1.39 - 6.78)	0,063
PC.aa.C36.6	4.56 (1.79 - 11.61)	0,016*	2.28 (0.91 - 5.69)	0,278	2.94 (1.32 - 6.58)	0,080
PC.aa.C38.0	1.2 (0.46 - 3.14)	0,839	1.13 (0.44 - 2.88)	0,910	1.01 (0.44 - 2.28)	0,993
PC.aa.C38.2	2.57 (0.99 - 6.63)	0,182	2.65 (1.06 - 6.65)	0,207	2.34 (1.04 - 5.26)	0,170
PC.aa.C38.3	3.91 (1.51 - 10.11)	0,042*	4.77 (1.89 - 12.02)	0,033*	4.1 (1.82 - 9.25)	0,023*
PC.aa.C38.4	2.42 (0.96 - 6.14)	0,193	3.64 (1.47 - 8.99)	0,078	2.4 (1.07 - 5.37)	0,155
PC.aa.C38.5	2.59 (1.03 - 6.47)	0,167	2.66 (1.09 - 6.48)	0,189	3.34 (1.52 - 7.36)	0,063
PC.aa.C38.6	1.5 (0.57 - 3.93)	0,605	1.52 (0.6 - 3.89)	0,605	1.84 (0.8 - 4.21)	0,372
PC.aa.C40.0	1.79 (0.69 - 4.69)	0,426	0.98 (0.38 - 2.5)	0,974	0.93 (0.41 - 2.1)	0,944
PC.aa.C40.1	1.81 (0.71 - 4.63)	0,412	2.04 (0.82 - 5.07)	0,362	1.17 (0.52 - 2.61)	0,873
PC.aa.C40.2	4.1 (1.63 - 10.31)	0,027*	1.79 (0.73 - 4.38)	0,421	2.46 (1.12 - 5.44)	0,135
PC.aa.C40.3	2.58 (1.02 - 6.53)	0,168	1.93 (0.78 - 4.77)	0,385	2.46 (1.11 - 5.48)	0,135
PC.aa.C40.4	3.69 (1.46 - 9.32)	0,044*	4.39 (1.79 - 10.77)	0,037*	4 (1.81 - 8.83)	0,023*
PC.aa.C40.5	2.04 (0.82 - 5.05)	0,307	3.5 (1.45 - 8.43)	0,078	3.08 (1.41 - 6.74)	0,063
PC.aa.C40.6	2.04 (0.79 - 5.25)	0,329	1.97 (0.78 - 4.95)	0,385	2.34 (1.04 - 5.3)	0,170
PC.aa.C42.0	1.13 (0.45 - 2.86)	0,890	1.04 (0.42 - 2.58)	0,968	1.61 (0.73 - 3.54)	0,495
PC.aa.C42.5	1.38 (0.55 - 3.46)	0,694	0.96 (0.39 - 2.35)	0,968	1.27 (0.58 - 2.78)	0,813
PC.aa.C43.6	0.88 (0.34 - 2.23)	0,885	1.31 (0.53 - 3.25)	0,755	1.22 (0.55 - 2.71)	0,864
PC.aa.C44.12	1.3 (0.51 - 3.32)	0,763	1.75 (0.7 - 4.37)	0,440	1.58 (0.7 - 3.55)	0,498
PC.ae.C30.0	3.85 (1.53 - 9.68)	0,039*	2.38 (0.97 - 5.85)	0,233	1.96 (0.89 - 4.33)	0,306
PC.ae.C32.0	2.54 (1 - 6.42)	0,178	2.53 (1.04 - 6.19)	0,208	1.91 (0.87 - 4.23)	0,307
PC.ae.C32.1	1.94 (0.75 - 4.99)	0,356	2.52 (1.02 - 6.26)	0,208	1.18 (0.52 - 2.64)	0,873
PC.ae.C32.2	1.58 (0.62 - 3.97)	0,523	1.43 (0.59 - 3.48)	0,654	0.75 (0.34 - 1.66)	0,742
PC.ae.C34.0	2.81 (1.11 - 7.1)	0,120	2.56 (1.04 - 6.27)	0,208	1.71 (0.77 - 3.78)	0,417
PC.ae.C34.1	2.22 (0.88 - 5.63)	0,257	2.1 (0.85 - 5.15)	0,320	1.82 (0.82 - 4.05)	0,354
PC.ae.C34.2	1.63 (0.63 - 4.23)	0,514	1.52 (0.61 - 3.8)	0,605	1.15 (0.51 - 2.6)	0,884
PC.ae.C34.3	1.66 (0.67 - 4.14)	0,473	1.63 (0.67 - 3.92)	0,514	0.86 (0.39 - 1.9)	0,873
PC.ae.C34.4	1.84 (0.72 - 4.71)	0,399	1.38 (0.56 - 3.42)	0,705	1.49 (0.66 - 3.32)	0,596
PC.ae.C36.0	0.97 (0.38 - 2.46)	0,983	1.79 (0.73 - 4.4)	0,421	1.09 (0.49 - 2.43)	0,933
PC.ae.C36.1	1.83 (0.72 - 4.64)	0,399	2.45 (0.99 - 6.05)	0,225	2.53 (1.14 - 5.6)	0,134
PC.ae.C36.2	1.12 (0.43 - 2.91)	0,902	1.04 (0.42 - 2.61)	0,968	1.07 (0.48 - 2.41)	0,948
PC.ae.C36.3	1.88 (0.74 - 4.76)	0,381	1.78 (0.72 - 4.4)	0,428	1.84 (0.83 - 4.09)	0,351
PC.ae.C36.4	2.52 (1.01 - 6.27)	0,175	4.2 (1.73 - 10.2)	0,040*	2.19 (1 - 4.81)	0,200
PC.ae.C36.5	2.02 (0.81 - 5)	0,317	2.22 (0.92 - 5.39)	0,278	1.42 (0.65 - 3.11)	0,640
PC.ae.C38.0	1.64 (0.63 - 4.26)	0,513	1.03 (0.41 - 2.6)	0,968	1.59 (0.7 - 3.59)	0,498
PC.ae.C38.2	1.99 (0.76 - 5.18)	0,352	2.35 (0.93 - 5.93)	0,271	1.31 (0.57 - 2.97)	0,800
PC.ae.C38.3	2.5 (0.97 - 6.42)	0,191	3.6 (1.44 - 8.97)	0,081	3.11 (1.39 - 6.98)	0,063
PC.ae.C38.4	1.94 (0.78 - 4.82)	0,346	3.08 (1.27 - 7.47)	0,137	1.93 (0.88 - 4.25)	0,306
PC.ae.C38.5	1.67 (0.67 - 4.19)	0,468	2.26 (0.93 - 5.52)	0,276	1.57 (0.71 - 3.46)	0,498
PC.ae.C38.6	1.72 (0.67 - 4.38)	0,454	1.77 (0.71 - 4.43)	0,440	1.59 (0.71 - 3.54)	0,498
PC.ae.C40.0	1.79 (0.67 - 4.73)	0,439	1.15 (0.45 - 2.97)	0,892	1.48 (0.64 - 3.43)	0,628
PC.ae.C40.1	2.97 (1.13 - 7.8)	0,118	1.33 (0.52 - 3.42)	0,755	1.74 (0.76 - 3.98)	0,422
PC.ae.C40.2	1.22 (0.48 - 3.14)	0,828	0.97 (0.39 - 2.42)	0,968	2.13 (0.96 - 4.74)	0,230
PC.ae.C40.3	2.08 (0.8 - 5.37)	0,318	2.25 (0.89 - 5.69)	0,288	1.63 (0.72 - 3.7)	0,498

PC.ae.C40.4	2.18 (0.86 - 5.53)	0,263	3.52 (1.43 - 8.7)	0,081	2.04 (0.91 - 4.57)	0,289
PC.ae.C40.5	1.14 (0.45 - 2.91)	0,885	1.55 (0.63 - 3.84)	0,579	1.61 (0.72 - 3.6)	0,498
PC.ae.C40.6	1.02 (0.39 - 2.63)	0,988	1.22 (0.49 - 3.06)	0,831	1.48 (0.65 - 3.34)	0,613
PC.ae.C42.1	2.17 (0.83 - 5.65)	0,286	2.25 (0.88 - 5.71)	0,288	1.6 (0.71 - 3.64)	0,498
PC.ae.C42.3	0.86 (0.33 - 2.26)	0,881	0.64 (0.25 - 1.62)	0,579	1.17 (0.51 - 2.66)	0,873
PC.ae.C42.4	2.18 (0.86 - 5.56)	0,263	2.17 (0.88 - 5.38)	0,300	1.94 (0.87 - 4.31)	0,306
PC.ae.C42.5	1.25 (0.49 - 3.18)	0,804	1.74 (0.7 - 4.32)	0,440	1.79 (0.8 - 4.01)	0,378
PC.ae.C42.6	1.02 (0.4 - 2.62)	0,988	0.95 (0.38 - 2.37)	0,968	1.16 (0.52 - 2.59)	0,873
SM.a.C30.1	2.27 (0.9 - 5.75)	0,241	2.53 (1.03 - 6.2)	0,208	1.93 (0.88 - 4.24)	0,306
SM.a.C32.1	1.84 (0.72 - 4.7)	0,399	2.41 (0.98 - 5.94)	0,233	2 (0.9 - 4.45)	0,302
SM.a.C32.2	1.86 (0.7 - 4.93)	0,412	3.18 (1.25 - 8.11)	0,149	3.17 (1.4 - 7.19)	0,063
SM.a.C33.1	1.02 (0.39 - 2.63)	0,988	1.54 (0.62 - 3.8)	0,582	1.27 (0.57 - 2.84)	0,816
SM.a.C34.1	1.01 (0.39 - 2.63)	0,988	1.63 (0.65 - 4.09)	0,531	1.3 (0.57 - 2.93)	0,801
SM.a.C34.2	1.12 (0.41 - 3.05)	0,910	2 (0.76 - 5.29)	0,385	1.67 (0.71 - 3.9)	0,495
SM.a.C35.0	0.77 (0.3 - 1.96)	0,761	0.88 (0.36 - 2.17)	0,900	1.25 (0.56 - 2.79)	0,846
SM.a.C35.1	0.37 (0.14 - 0.98)	0,168	0.83 (0.33 - 2.09)	0,837	0.73 (0.33 - 1.65)	0,711
SM.a.C36.1	0.74 (0.28 - 1.96)	0,738	1.46 (0.57 - 3.75)	0,648	1 (0.44 - 2.3)	0,993
SM.a.C36.2	0.43 (0.16 - 1.18)	0,263	1.19 (0.45 - 3.16)	0,871	0.88 (0.37 - 2.07)	0,893
SM.a.C36.3	0.76 (0.28 - 2.06)	0,765	0.88 (0.34 - 2.31)	0,910	0.99 (0.43 - 2.31)	0,993
SM.a.C37.1	1.22 (0.47 - 3.17)	0,829	1.29 (0.51 - 3.22)	0,755	2.18 (0.98 - 4.85)	0,214
SM.a.C38.2	1.29 (0.5 - 3.36)	0,768	1.9 (0.77 - 4.71)	0,385	1.3 (0.58 - 2.9)	0,800
SM.a.C38.3	1.24 (0.48 - 3.16)	0,810	1.49 (0.61 - 3.65)	0,605	0.96 (0.44 - 2.13)	0,985
SM.a.C39.1	2.2 (0.83 - 5.83)	0,286	2.26 (0.89 - 5.76)	0,288	2.32 (1.02 - 5.29)	0,180
SM.a.C39.2	0.73 (0.28 - 1.93)	0,723	0.76 (0.3 - 1.91)	0,755	1.21 (0.54 - 2.73)	0,864
SM.a.C40.2	1.88 (0.73 - 4.83)	0,384	2.83 (1.14 - 7)	0,174	2.02 (0.91 - 4.5)	0,295
SM.a.C40.5	3.42 (1.36 - 8.59)	0,056	2.05 (0.83 - 5.03)	0,351	4.07 (1.86 - 8.9)	0,023*
SM.a.C41.1	2.9 (1.14 - 7.36)	0,111	1.94 (0.79 - 4.72)	0,385	1.71 (0.78 - 3.75)	0,417
SM.a.C41.2	1.33 (0.52 - 3.41)	0,744	1.03 (0.42 - 2.54)	0,968	1.99 (0.9 - 4.4)	0,305
SM.a.C42.1	1.51 (0.6 - 3.82)	0,579	1.06 (0.43 - 2.6)	0,968	1.3 (0.59 - 2.86)	0,800
SM.a.C42.2	1.09 (0.42 - 2.84)	0,930	0.85 (0.34 - 2.16)	0,875	1.42 (0.63 - 3.22)	0,656
SM.a.C42.3	1 (0.38 - 2.61)	0,992	1.56 (0.62 - 3.95)	0,579	1.59 (0.7 - 3.6)	0,498
SM.a.C42.4	2.4 (0.95 - 6.03)	0,193	2.78 (1.13 - 6.81)	0,174	1.95 (0.88 - 4.33)	0,306
SM.a.C42.6	1.96 (0.75 - 5.11)	0,356	1.95 (0.77 - 4.94)	0,385	1.94 (0.85 - 4.43)	0,319
SM.a.C43.1	1.59 (0.61 - 4.13)	0,523	1.07 (0.42 - 2.69)	0,966	1.05 (0.47 - 2.37)	0,969
SM.a.C43.2	1.17 (0.46 - 2.98)	0,874	1.22 (0.49 - 3.04)	0,831	0.93 (0.42 - 2.08)	0,944
SM.a.C44.6	1.39 (0.54 - 3.6)	0,694	1.63 (0.65 - 4.12)	0,534	1.86 (0.82 - 4.18)	0,351
SM.e.C36.2	0.47 (0.18 - 1.27)	0,320	0.73 (0.28 - 1.87)	0,720	0.89 (0.39 - 2.05)	0,906
SM.e.C38.3	0.97 (0.39 - 2.45)	0,983	1.28 (0.53 - 3.13)	0,755	1.02 (0.46 - 2.24)	0,993
SM.e.C40.5	1.49 (0.6 - 3.71)	0,590	1.85 (0.76 - 4.51)	0,398	1.11 (0.51 - 2.43)	0,906
Carn	1.14 (0.44 - 2.95)	0,885	1.93 (0.78 - 4.77)	0,385	2.5 (1.13 - 5.56)	0,135
Carn.a.C10.0	0.47 (0.19 - 1.18)	0,274	0.83 (0.34 - 2.04)	0,834	1.03 (0.46 - 2.29)	0,985
Carn.a.C10.1	0.56 (0.22 - 1.43)	0,417	0.6 (0.24 - 1.5)	0,514	0.84 (0.37 - 1.87)	0,873
Carn.a.C12.0	0.68 (0.27 - 1.72)	0,608	0.82 (0.33 - 2.01)	0,831	1.17 (0.53 - 2.58)	0,873
Carn.a.C14.1	0.61 (0.24 - 1.52)	0,487	0.65 (0.26 - 1.59)	0,579	1 (0.45 - 2.21)	0,993
Carn.a.C14.2	0.52 (0.21 - 1.3)	0,356	0.56 (0.23 - 1.35)	0,416	0.97 (0.44 - 2.13)	0,985

Carn.a.C15.0	0.89 (0.36 - 2.24)	0,902	1 (0.41 - 2.42)	0,999	1.74 (0.79 - 3.83)	0,399
Carn.a.C16.0	0.74 (0.3 - 1.85)	0,720	0.84 (0.34 - 2.03)	0,837	0.89 (0.4 - 1.95)	0,893
Carn.a.C16.0.Oxo	1.11 (0.44 - 2.83)	0,910	0.96 (0.38 - 2.39)	0,968	1.03 (0.46 - 2.32)	0,985
Carn.a.C16.1	0.34 (0.14 - 0.86)	0,108	0.56 (0.23 - 1.37)	0,421	1.19 (0.53 - 2.64)	0,873
Carn.a.C16.2	0.57 (0.23 - 1.41)	0,417	0.65 (0.27 - 1.56)	0,579	1.19 (0.54 - 2.61)	0,873
Carn.a.C18.0	1.21 (0.48 - 3.01)	0,834	0.51 (0.21 - 1.23)	0,367	0.99 (0.45 - 2.19)	0,993
Carn.a.C18.1	0.53 (0.21 - 1.35)	0,381	0.73 (0.29 - 1.81)	0,708	1.43 (0.64 - 3.18)	0,640
Carn.a.C18.2	0.68 (0.27 - 1.69)	0,598	0.91 (0.37 - 2.21)	0,923	0.94 (0.43 - 2.07)	0,962
Carn.a.C18.2.OH	0.93 (0.38 - 2.31)	0,938	0.87 (0.36 - 2.1)	0,887	1.22 (0.55 - 2.68)	0,864
Carn.a.C2.0	1.69 (0.67 - 4.26)	0,465	1.82 (0.75 - 4.45)	0,416	1.85 (0.83 - 4.1)	0,347
Carn.a.C20.0	1.45 (0.57 - 3.65)	0,618	1.87 (0.77 - 4.53)	0,391	0.85 (0.38 - 1.87)	0,873
Carn.a.C20.1	1.58 (0.64 - 3.91)	0,520	1.03 (0.43 - 2.46)	0,968	1.38 (0.64 - 3.01)	0,660
Carn.a.C20.3	0.91 (0.37 - 2.24)	0,914	1.25 (0.52 - 2.99)	0,786	0.7 (0.32 - 1.53)	0,640
Carn.a.C20.4	0.77 (0.31 - 1.92)	0,760	1.03 (0.42 - 2.48)	0,968	0.95 (0.43 - 2.09)	0,969
Carn.a.C3.0	2.9 (1.16 - 7.23)	0,107	2.28 (0.94 - 5.51)	0,263	2.32 (1.06 - 5.07)	0,162
Carn.a.C3.0.DC	0.79 (0.31 - 2)	0,784	1.06 (0.43 - 2.62)	0,968	1.6 (0.72 - 3.58)	0,498
Carn.a.C4.0	3.54 (1.43 - 8.75)	0,044*	2.36 (0.97 - 5.7)	0,233	1.9 (0.87 - 4.16)	0,306
Carn.a.C5.0	2.9 (1.16 - 7.21)	0,107	2.47 (1.02 - 6.02)	0,208	2.82 (1.29 - 6.2)	0,084
Carn.a.C6.0	0.86 (0.34 - 2.18)	0,874	1.08 (0.44 - 2.67)	0,958	1.09 (0.49 - 2.42)	0,934
Carn.a.C6.0.OH	0.58 (0.23 - 1.48)	0,448	1.22 (0.49 - 3.03)	0,831	1.75 (0.78 - 3.91)	0,406
Carn.a.C8.0	0.51 (0.2 - 1.29)	0,346	0.74 (0.3 - 1.83)	0,730	0.86 (0.38 - 1.91)	0,873
Carn.a.C8.1	0.85 (0.33 - 2.19)	0,865	1.03 (0.41 - 2.6)	0,968	0.7 (0.31 - 1.59)	0,656
Carn.a.C9.0	1.63 (0.65 - 4.08)	0,504	1.29 (0.53 - 3.16)	0,755	1.68 (0.76 - 3.7)	0,436
Asn/Asp	0.97 (0.39 - 2.38)	0,983	0.45 (0.19 - 1.08)	0,278	0.79 (0.36 - 1.71)	0,811
Gln/Glu	1.04 (0.41 - 2.62)	0,983	0.37 (0.15 - 0.89)	0,175	0.36 (0.16 - 0.81)	0,095
NEFA18.1/NEFA18.0	0.83 (0.33 - 2.13)	0,839	0.65 (0.26 - 1.61)	0,581	0.9 (0.4 - 2.02)	0,906
NEFA16.1/NEFA16.0	0.44 (0.17 - 1.15)	0,257	0.66 (0.26 - 1.67)	0,605	1.22 (0.53 - 2.79)	0,864
PC.aa/PC.ae	3.66 (1.45 - 9.27)	0,044*	2.21 (0.9 - 5.41)	0,288	2.29 (1.04 - 5.07)	0,170
Lyso.PC.a/PC.aa	1.53 (0.59 - 3.95)	0,578	2 (0.82 - 4.89)	0,362	1.11 (0.5 - 2.47)	0,906
(lyso.PC.a.C16.0 + lyso.PC.a.C18.0)/ PC.aa	0.97 (0.37 - 2.51)	0,983	2.11 (0.86 - 5.2)	0,320	1.13 (0.5 - 2.54)	0,893
(lyso.PC.a.C18.1 + lyso.PC.a.C18.2)	2.42 (0.95 - 6.12)	0,193	1.19 (0.49 - 2.89)	0,837	0.79 (0.36 - 1.74)	0,813
Carn.a.C.16.0/ free Carn	0.69 (0.27 - 1.72)	0,608	0.61 (0.25 - 1.48)	0,511	0.52 (0.24 - 1.15)	0,306
Carn.a.C2.0/ Carn.a.C16.0	1.89 (0.76 - 4.71)	0,356	1.89 (0.78 - 4.58)	0,385	1.7 (0.77 - 3.74)	0,417

Values represent absolute differences in blood pressure (95% confidence interval) and corresponding p-values from linear regression models that reflect the difference in blood pressure (mmHg) per SDS increase in maternal early-pregnancy metabolite concentrations (μmol/L) or metabolite ratio. Model includes gestational age at time of measurement, age, parity, pre-pregnancy body mass index, educational level, smoking and folic acid supplementation. AA amino acids, NEFA non-esterified fatty acids, PC.aa diacyl-phosphatidylcholines, PC.ae acyl-alkyl-phosphatidylcholines, lyso.PC.a acyl-lysophosphatidylcholines, lyso.PC.e alkyl-lysophosphatidylcholines, Carn.a acyl-carnitines, SM sphingomyelins

[a] p-value corrected for multiple hypothesis testing using Benjamin-Hochberg FDR correction.

*****Statistically significant

Table S5. Associations of early-pregnancy individual metabolites with diastolic blood pressure in early-, mid- and late pregnancy. Full model.

	**Differences in diastolic blood pressure in mmHg (95% confidence interval)**					
**Metabolite**	**Early pregnancy** N = 803	**P-value**[a]	**Mid pregnancy** N = 793	**P-value**[a]	**Late pregnancy** N = 800	**P-value**[a]
Ala	1.1 (0.55 - 2.18)	0,839	1.55 (0.8 - 3.02)	0,419	2.41 (1.29 - 4.51)	0,054
Arg	1.95 (0.98 - 3.87)	0,147	1.13 (0.58 - 2.2)	0,838	1.78 (0.95 - 3.33)	0,136
Asn	0.95 (0.47 - 1.88)	0,908	0.84 (0.43 - 1.64)	0,769	1.81 (0.96 - 3.4)	0,131
Asp	2.69 (1.34 - 5.41)	0,038*	1.77 (0.91 - 3.43)	0,280	3.15 (1.68 - 5.9)	0,009
Cit	0.98 (0.47 - 2.04)	0,977	0.73 (0.36 - 1.46)	0,594	1.14 (0.58 - 2.21)	0,728
Gln	1.31 (0.66 - 2.59)	0,542	0.68 (0.35 - 1.31)	0,461	0.78 (0.42 - 1.47)	0,503
Glu	2.67 (1.34 - 5.31)	0,038*	2.02 (1.04 - 3.92)	0,203	3.75 (2.01 - 6.99)	0,008*
Gly	0.77 (0.39 - 1.52)	0,552	0.9 (0.47 - 1.72)	0,852	1.6 (0.86 - 2.98)	0,212
His	0.99 (0.49 - 2)	0,983	0.83 (0.42 - 1.64)	0,769	1.98 (1.04 - 3.77)	0,102
Ile	0.67 (0.34 - 1.35)	0,385	0.83 (0.42 - 1.63)	0,769	1.47 (0.78 - 2.79)	0,300
Leu	0.79 (0.4 - 1.57)	0,591	0.84 (0.43 - 1.63)	0,769	1.72 (0.92 - 3.21)	0,152
Lys	0.47 (0.23 - 0.93)	0,099	1.19 (0.61 - 2.31)	0,769	2.21 (1.18 - 4.14)	0,064
Met	0.97 (0.49 - 1.93)	0,957	0.88 (0.45 - 1.71)	0,814	1.27 (0.68 - 2.38)	0,508
Orn	0.68 (0.34 - 1.36)	0,390	1 (0.51 - 1.94)	0,999	2.13 (1.13 - 4.01)	0,071
Phe	1.71 (0.87 - 3.36)	0,216	1.27 (0.66 - 2.44)	0,687	2.08 (1.12 - 3.84)	0,071
Pro	0.96 (0.49 - 1.9)	0,939	0.81 (0.42 - 1.55)	0,715	1.43 (0.77 - 2.67)	0,316
Trp	1.11 (0.56 - 2.22)	0,821	1.05 (0.54 - 2.04)	0,927	1.48 (0.79 - 2.76)	0,289
Ser	1.57 (0.79 - 3.1)	0,306	0.97 (0.5 - 1.85)	0,939	2.1 (1.13 - 3.91)	0,071
Thr	0.82 (0.41 - 1.66)	0,669	1.04 (0.52 - 2.05)	0,939	1.65 (0.87 - 3.12)	0,199
Tyr	1.29 (0.65 - 2.54)	0,566	0.95 (0.49 - 1.83)	0,927	1.62 (0.87 - 3.01)	0,202
Val	1.24 (0.63 - 2.47)	0,615	0.95 (0.49 - 1.82)	0,926	1.72 (0.92 - 3.21)	0,152
Cys	0.36 (0.18 - 0.73)	0,038*	0.71 (0.36 - 1.39)	0,521	0.99 (0.52 - 1.89)	0,977
NEFA_14_0	2.16 (1.09 - 4.27)	0,094	1.37 (0.71 - 2.65)	0,553	1.1 (0.59 - 2.05)	0,786
NEFA_14_1	3.23 (1.63 - 6.42)	0,019*	2 (1.03 - 3.88)	0,203	1.39 (0.73 - 2.62)	0,377
NEFA_15_0	2.19 (1.11 - 4.31)	0,091	1.49 (0.78 - 2.87)	0,443	0.87 (0.46 - 1.62)	0,675
NEFA_16_0	2.78 (1.4 - 5.52)	0,037*	1.72 (0.88 - 3.33)	0,309	1.46 (0.78 - 2.76)	0,300
NEFA_16_1	3.3 (1.66 - 6.56)	0,018*	2.2 (1.14 - 4.27)	0,144	2.16 (1.15 - 4.06)	0,067
NEFA_16_2	2.62 (1.32 - 5.23)	0,043*	1.59 (0.81 - 3.09)	0,407	1.62 (0.86 - 3.06)	0,208
NEFA_17_0	2.25 (1.14 - 4.41)	0,085	1.66 (0.86 - 3.19)	0,340	1.17 (0.63 - 2.18)	0,646
NEFA_17_1	2.69 (1.36 - 5.31)	0,038*	1.94 (1.01 - 3.73)	0,217	1.27 (0.68 - 2.38)	0,509
NEFA_17_2	1.81 (0.91 - 3.59)	0,187	1.08 (0.56 - 2.1)	0,883	1.37 (0.73 - 2.56)	0,390
NEFA_18_0	2.49 (1.25 - 4.97)	0,054	1.27 (0.65 - 2.47)	0,704	0.96 (0.51 - 1.83)	0,917
NEFA_18_1	2.92 (1.48 - 5.79)	0,032*	1.56 (0.81 - 3.02)	0,414	1.87 (1 - 3.51)	0,111
NEFA_18_2	2.38 (1.21 - 4.69)	0,064	1.16 (0.6 - 2.24)	0,788	1.33 (0.71 - 2.48)	0,428
NEFA_18_3	1.72 (0.87 - 3.4)	0,216	1.09 (0.57 - 2.1)	0,872	1.26 (0.68 - 2.35)	0,510
NEFA_19_1	2.26 (1.15 - 4.44)	0,085	1.45 (0.76 - 2.79)	0,468	1.21 (0.65 - 2.27)	0,574
NEFA_20_1	2.93 (1.47 - 5.81)	0,032*	1.37 (0.7 - 2.66)	0,566	2.17 (1.16 - 4.09)	0,066
NEFA_20_2	2.73 (1.38 - 5.4)	0,038*	2.01 (1.04 - 3.88)	0,203	2.29 (1.22 - 4.29)	0,064
NEFA_20_3	3.45 (1.7 - 7)	0,018*	2.8 (1.43 - 5.52)	0,049*	1.91 (1 - 3.66)	0,109
NEFA_20_4	3.82 (1.94 - 7.53)	0,007*	2.02 (1.05 - 3.88)	0,203	2.68 (1.44 - 4.99)	0,032*

NEFA_20_5	2.08 (1.04 - 4.15)	0,113	1.2 (0.62 - 2.32)	0,769	2.09 (1.12 - 3.92)	0,071
NEFA_22_3	3.09 (1.55 - 6.14)	0,027*	3.01 (1.55 - 5.84)	0,047*	2.4 (1.27 - 4.55)	0,054
NEFA_22_4	4.51 (2.25 - 9.06)	0,005*	2.82 (1.43 - 5.53)	0,049*	2.73 (1.43 - 5.2)	0,033*
NEFA_22_5	3.99 (2.01 - 7.91)	0,007*	2.18 (1.12 - 4.22)	0,150	2.25 (1.19 - 4.24)	0,064
NEFA_22_6	3.56 (1.79 - 7.08)	0,012*	1.77 (0.91 - 3.44)	0,280	1.99 (1.06 - 3.75)	0,095
NEFA_24_0	1.25 (0.64 - 2.46)	0,605	0.9 (0.47 - 1.73)	0,856	0.95 (0.51 - 1.76)	0,883
NEFA_24_1	1.51 (0.76 - 2.98)	0,351	0.79 (0.41 - 1.52)	0,691	1.7 (0.91 - 3.18)	0,158
NEFA_24_2	2.46 (1.22 - 4.95)	0,061	1.44 (0.74 - 2.81)	0,489	1.62 (0.85 - 3.07)	0,212
NEFA_24_4	2.8 (1.41 - 5.54)	0,034*	2.58 (1.34 - 4.96)	0,058	2.19 (1.18 - 4.1)	0,064
NEFA_24_5	3.85 (1.93 - 7.65)	0,007*	2.99 (1.55 - 5.79)	0,047*	3.15 (1.68 - 5.92)	0,009*
NEFA_26_0	2.54 (1.29 - 5)	0,046*	1.22 (0.63 - 2.34)	0,755	1.47 (0.79 - 2.74)	0,297
NEFA_26_1	2.82 (1.42 - 5.61)	0,034*	1.84 (0.94 - 3.58)	0,250	1.75 (0.93 - 3.29)	0,151
NEFA_26_2	2.28 (1.12 - 4.65)	0,091	1.61 (0.81 - 3.19)	0,407	1.35 (0.7 - 2.6)	0,428
lyso.PC.a.C14.0	2.33 (1.15 - 4.69)	0,085	3.44 (1.75 - 6.76)	0,047*	3.07 (1.61 - 5.83)	0,015*
lyso.PC.a.C16.0	1.51 (0.74 - 3.07)	0,370	2.77 (1.41 - 5.44)	0,050	3.35 (1.77 - 6.37)	0,009*
lyso.PC.a.C16.1	1.96 (0.95 - 4.06)	0,162	2.91 (1.46 - 5.82)	0,049*	3.81 (1.97 - 7.35)	0,008*
lyso.PC.a.C18.0	1.33 (0.66 - 2.71)	0,537	1.91 (0.97 - 3.76)	0,237	2.29 (1.21 - 4.35)	0,064
lyso.PC.a.C18.1	1.82 (0.91 - 3.64)	0,187	1.82 (0.94 - 3.54)	0,255	2.69 (1.44 - 5.03)	0,032*
lyso.PC.a.C18.2	1.25 (0.62 - 2.49)	0,615	0.92 (0.48 - 1.78)	0,880	1.23 (0.65 - 2.31)	0,559
lyso.PC.a.C18.3	1.13 (0.58 - 2.21)	0,794	1.08 (0.57 - 2.05)	0,890	1.89 (1.02 - 3.48)	0,102
lyso.PC.a.C20.3	2.34 (1.17 - 4.65)	0,077	2.8 (1.44 - 5.43)	0,049*	2.26 (1.21 - 4.24)	0,064
lyso.PC.a.C20.4	3.09 (1.55 - 6.18)	0,027*	2.66 (1.37 - 5.14)	0,052	2.67 (1.43 - 5.01)	0,032*
lyso.PC.a.C20.5	1.25 (0.62 - 2.51)	0,614	1.41 (0.73 - 2.7)	0,511	2.02 (1.09 - 3.76)	0,079
lyso.PC.a.C22.6	1.62 (0.81 - 3.24)	0,284	1.46 (0.76 - 2.83)	0,468	1.46 (0.78 - 2.74)	0,300
lyso.PC.e.C16.0	1.16 (0.58 - 2.33)	0,760	1.89 (0.97 - 3.68)	0,237	1.83 (0.97 - 3.46)	0,129
lyso.PC.e.C18.0	1.01 (0.51 - 2.01)	0,983	2.08 (1.08 - 4)	0,184	1.63 (0.87 - 3.06)	0,201
lyso.PC.e.C18.1	1.8 (0.88 - 3.67)	0,210	2.01 (1.01 - 3.98)	0,217	2.25 (1.18 - 4.31)	0,064
PC.aa.C30.0	2.05 (1.03 - 4.08)	0,117	1.76 (0.91 - 3.41)	0,284	1.47 (0.78 - 2.77)	0,300
PC.aa.C30.3	1.76 (0.88 - 3.51)	0,210	1.41 (0.73 - 2.73)	0,511	1.31 (0.69 - 2.49)	0,459
PC.aa.C32.0	2.89 (1.44 - 5.8)	0,034*	2.22 (1.14 - 4.32)	0,144	2.41 (1.27 - 4.57)	0,054
PC.aa.C32.1	2.53 (1.26 - 5.11)	0,054	2.78 (1.42 - 5.44)	0,049	2.59 (1.36 - 4.92)	0,043*
PC.aa.C32.2	1.87 (0.93 - 3.75)	0,169	2.16 (1.12 - 4.19)	0,152	1.64 (0.87 - 3.1)	0,200
PC.aa.C32.3	2.19 (1.07 - 4.48)	0,104	2.13 (1.08 - 4.21)	0,184	2.4 (1.25 - 4.6)	0,060
PC.aa.C34.1	2.27 (1.13 - 4.53)	0,088	2.26 (1.17 - 4.38)	0,126	2.56 (1.36 - 4.82)	0,043*
PC.aa.C34.2	1.59 (0.78 - 3.21)	0,308	1.47 (0.76 - 2.87)	0,468	1.79 (0.94 - 3.39)	0,140
PC.aa.C34.3	1.55 (0.77 - 3.12)	0,336	1.87 (0.96 - 3.64)	0,237	2.03 (1.07 - 3.84)	0,087
PC.aa.C34.4	2.44 (1.24 - 4.81)	0,054	2.49 (1.3 - 4.78)	0,068	1.72 (0.92 - 3.21)	0,152
PC.aa.C34.5	2.31 (1.13 - 4.69)	0,088	1.61 (0.81 - 3.19)	0,407	1.84 (0.96 - 3.52)	0,131
PC.aa.C36.0	1.83 (0.9 - 3.73)	0,195	1.71 (0.86 - 3.4)	0,335	1.76 (0.92 - 3.38)	0,152
PC.aa.C36.1	2.16 (1.09 - 4.29)	0,094	1.51 (0.78 - 2.93)	0,438	2.33 (1.24 - 4.37)	0,060
PC.aa.C36.2	1.58 (0.78 - 3.2)	0,309	1.31 (0.67 - 2.57)	0,651	1.61 (0.85 - 3.07)	0,215
PC.aa.C36.3	1.75 (0.86 - 3.55)	0,221	1.88 (0.96 - 3.68)	0,237	1.84 (0.97 - 3.5)	0,128
PC.aa.C36.4	2.53 (1.27 - 5.05)	0,052	2.81 (1.45 - 5.42)	0,049	2.23 (1.18 - 4.21)	0,064
PC.aa.C36.5	1.78 (0.89 - 3.56)	0,201	1.8 (0.92 - 3.5)	0,269	2.54 (1.35 - 4.78)	0,043*
PC.aa.C36.6	1.44 (0.71 - 2.9)	0,430	1.81 (0.92 - 3.56)	0,269	1.94 (1.02 - 3.69)	0,102

PC.aa.C38.0	1.4 (0.68 - 2.86)	0,465	1.19 (0.59 - 2.37)	0,772	1.97 (1.03 - 3.79)	0,102
PC.aa.C38.2	1.38 (0.68 - 2.8)	0,481	1.89 (0.96 - 3.72)	0,237	1.82 (0.96 - 3.48)	0,131
PC.aa.C38.3	2.14 (1.05 - 4.37)	0,109	2.47 (1.25 - 4.89)	0,096	2.12 (1.11 - 4.06)	0,074
PC.aa.C38.4	2.77 (1.39 - 5.54)	0,038*	2.54 (1.31 - 4.94)	0,068	2.3 (1.22 - 4.35)	0,064
PC.aa.C38.5	2.18 (1.1 - 4.33)	0,093	2.32 (1.21 - 4.47)	0,111	2.7 (1.44 - 5.05)	0,032*
PC.aa.C38.6	1.64 (0.8 - 3.36)	0,294	1.69 (0.84 - 3.37)	0,360	1.98 (1.02 - 3.83)	0,102
PC.aa.C40.0	1.91 (0.93 - 3.91)	0,170	1.06 (0.53 - 2.13)	0,926	1.59 (0.83 - 3.07)	0,236
PC.aa.C40.1	2.61 (1.3 - 5.26)	0,046*	1.48 (0.75 - 2.91)	0,468	1.38 (0.73 - 2.62)	0,388
PC.aa.C40.2	2.69 (1.35 - 5.36)	0,038*	1.56 (0.81 - 3.04)	0,414	1.93 (1.03 - 3.63)	0,102
PC.aa.C40.3	2.88 (1.44 - 5.75)	0,034*	2.22 (1.14 - 4.32)	0,144	1.92 (1.02 - 3.63)	0,105
PC.aa.C40.4	3.34 (1.68 - 6.64)	0,018*	3.1 (1.6 - 6.01)	0,047*	2.34 (1.24 - 4.39)	0,060
PC.aa.C40.5	2.08 (1.06 - 4.1)	0,107	2.35 (1.23 - 4.49)	0,096	2.1 (1.13 - 3.91)	0,071
PC.aa.C40.6	2.08 (1.03 - 4.21)	0,120	1.72 (0.87 - 3.39)	0,316	2.1 (1.1 - 4)	0,075
PC.aa.C42.0	1.68 (0.84 - 3.34)	0,249	1.28 (0.66 - 2.51)	0,687	1.92 (1.02 - 3.59)	0,102
PC.aa.C42.5	1.86 (0.94 - 3.7)	0,169	1.45 (0.75 - 2.81)	0,478	1.82 (0.97 - 3.41)	0,127
PC.aa.C43.6	1.45 (0.72 - 2.92)	0,414	1.61 (0.82 - 3.15)	0,407	1.92 (1.01 - 3.62)	0,105
PC.aa.C44.12	1.75 (0.87 - 3.52)	0,216	1.54 (0.78 - 3.02)	0,433	2.22 (1.17 - 4.22)	0,064
PC.ae.C30.0	1.97 (0.99 - 3.93)	0,143	1.74 (0.9 - 3.38)	0,298	1.38 (0.73 - 2.6)	0,378
PC.ae.C32.0	1.83 (0.91 - 3.67)	0,187	1.67 (0.86 - 3.24)	0,345	1.66 (0.88 - 3.14)	0,189
PC.ae.C32.1	1.97 (0.97 - 3.99)	0,148	1.79 (0.91 - 3.51)	0,280	1.78 (0.93 - 3.4)	0,150
PC.ae.C32.2	1.58 (0.79 - 3.16)	0,306	1.16 (0.6 - 2.25)	0,793	1.39 (0.73 - 2.63)	0,376
PC.ae.C34.0	1.97 (0.98 - 3.93)	0,144	1.71 (0.88 - 3.33)	0,311	1.25 (0.66 - 2.36)	0,533
PC.ae.C34.1	1.65 (0.82 - 3.32)	0,269	1.51 (0.77 - 2.93)	0,443	1.74 (0.92 - 3.29)	0,152
PC.ae.C34.2	1.42 (0.7 - 2.89)	0,462	1.16 (0.58 - 2.31)	0,795	1.64 (0.85 - 3.16)	0,212
PC.ae.C34.3	1.46 (0.73 - 2.89)	0,400	1.14 (0.59 - 2.21)	0,809	1.47 (0.78 - 2.77)	0,300
PC.ae.C34.4	1.52 (0.75 - 3.07)	0,356	1.22 (0.62 - 2.4)	0,754	1.74 (0.91 - 3.31)	0,157
PC.ae.C36.0	1.9 (0.95 - 3.82)	0,165	1.94 (1 - 3.77)	0,227	2.09 (1.1 - 3.96)	0,075
PC.ae.C36.1	2.23 (1.11 - 4.45)	0,091	1.98 (1.02 - 3.86)	0,217	2.41 (1.28 - 4.53)	0,054
PC.ae.C36.2	1.15 (0.56 - 2.35)	0,773	1.05 (0.53 - 2.08)	0,932	1.19 (0.61 - 2.29)	0,639
PC.ae.C36.3	1.67 (0.83 - 3.36)	0,258	1.55 (0.79 - 3.04)	0,431	2.25 (1.19 - 4.26)	0,064
PC.ae.C36.4	2.8 (1.42 - 5.52)	0,034*	2.63 (1.37 - 5.06)	0,052	2.38 (1.28 - 4.44)	0,054
PC.ae.C36.5	2.5 (1.27 - 4.91)	0,050	2.05 (1.07 - 3.94)	0,184	2.16 (1.16 - 4.02)	0,064
PC.ae.C38.0	1.27 (0.62 - 2.61)	0,599	1.51 (0.76 - 2.99)	0,461	1.83 (0.95 - 3.53)	0,134
PC.ae.C38.2	1.41 (0.69 - 2.89)	0,464	1.32 (0.66 - 2.62)	0,651	1.25 (0.65 - 2.42)	0,543
PC.ae.C38.3	2.04 (1 - 4.14)	0,133	2.48 (1.26 - 4.87)	0,093	2.1 (1.1 - 4)	0,075
PC.ae.C38.4	2.06 (1.04 - 4.06)	0,113	1.93 (1 - 3.7)	0,223	1.91 (1.02 - 3.57)	0,102
PC.ae.C38.5	2.18 (1.1 - 4.32)	0,093	2 (1.03 - 3.85)	0,203	2.37 (1.27 - 4.43)	0,054
PC.ae.C38.6	2.23 (1.11 - 4.49)	0,091	1.74 (0.89 - 3.42)	0,307	2.45 (1.3 - 4.64)	0,054
PC.ae.C40.0	1.99 (0.96 - 4.14)	0,154	1.91 (0.95 - 3.85)	0,243	1.59 (0.81 - 3.11)	0,248
PC.ae.C40.1	1.81 (0.87 - 3.73)	0,210	1.35 (0.67 - 2.72)	0,626	1.58 (0.81 - 3.07)	0,248
PC.ae.C40.2	1.62 (0.8 - 3.28)	0,291	1.16 (0.59 - 2.3)	0,793	1.92 (1.01 - 3.65)	0,109
PC.ae.C40.3	2.23 (1.1 - 4.54)	0,094	1.56 (0.78 - 3.09)	0,433	1.49 (0.77 - 2.86)	0,300
PC.ae.C40.4	1.94 (0.97 - 3.87)	0,150	1.44 (0.74 - 2.81)	0,491	1.67 (0.88 - 3.17)	0,189
PC.ae.C40.5	1.76 (0.88 - 3.54)	0,210	1.34 (0.68 - 2.61)	0,622	2.23 (1.18 - 4.24)	0,064
PC.ae.C40.6	1.4 (0.69 - 2.85)	0,464	1.25 (0.63 - 2.48)	0,715	1.97 (1.03 - 3.77)	0,102

PC.ae.C42.1	2.79 (1.37 - 5.7)	0,038	3.23 (1.63 - 6.4)	0,047*	1.58 (0.82 - 3.04)	0,242
PC.ae.C42.3	1.07 (0.52 - 2.19)	0,903	0.68 (0.34 - 1.36)	0,483	1.26 (0.65 - 2.46)	0,533
PC.ae.C42.4	1.59 (0.79 - 3.21)	0,306	1.42 (0.73 - 2.78)	0,511	1.85 (0.98 - 3.5)	0,126
PC.ae.C42.5	1.59 (0.79 - 3.19)	0,306	1.19 (0.61 - 2.34)	0,769	1.93 (1.02 - 3.67)	0,105
PC.ae.C42.6	1.21 (0.6 - 2.45)	0,676	0.96 (0.49 - 1.9)	0,939	1.6 (0.84 - 3.04)	0,226
SM.a.C30.1	1.89 (0.94 - 3.81)	0,168	1.79 (0.92 - 3.48)	0,272	1.59 (0.85 - 3)	0,215
SM.a.C32.1	1.79 (0.88 - 3.62)	0,209	2.28 (1.17 - 4.43)	0,126	1.64 (0.86 - 3.11)	0,204
SM.a.C32.2	2.2 (1.06 - 4.59)	0,109	2.35 (1.18 - 4.65)	0,126	2.47 (1.28 - 4.76)	0,054
SM.a.C33.1	1.42 (0.69 - 2.9)	0,464	1.5 (0.76 - 2.96)	0,456	1.37 (0.72 - 2.64)	0,397
SM.a.C34.1	2.02 (0.99 - 4.12)	0,141	1.58 (0.8 - 3.12)	0,414	1.57 (0.82 - 3.01)	0,248
SM.a.C34.2	2.72 (1.28 - 5.76)	0,054	1.96 (0.96 - 3.98)	0,237	2.04 (1.03 - 4)	0,102
SM.a.C35.0	1.66 (0.82 - 3.35)	0,272	1.32 (0.67 - 2.6)	0,649	1.82 (0.95 - 3.5)	0,135
SM.a.C35.1	1.16 (0.56 - 2.39)	0,766	1.45 (0.73 - 2.87)	0,497	1.44 (0.75 - 2.78)	0,335
SM.a.C36.1	1.85 (0.89 - 3.82)	0,196	1.9 (0.96 - 3.79)	0,237	2.35 (1.22 - 4.53)	0,064
SM.a.C36.2	1.78 (0.84 - 3.79)	0,237	1.95 (0.96 - 3.98)	0,237	2.24 (1.13 - 4.42)	0,071
SM.a.C36.3	1.71 (0.81 - 3.64)	0,272	1.57 (0.77 - 3.18)	0,433	2.04 (1.04 - 4.01)	0,102
SM.a.C37.1	1.32 (0.64 - 2.69)	0,555	1.55 (0.79 - 3.04)	0,433	2.4 (1.27 - 4.55)	0,054
SM.a.C38.2	1.37 (0.67 - 2.8)	0,497	1.6 (0.82 - 3.14)	0,407	2.19 (1.15 - 4.18)	0,067
SM.a.C38.3	1.33 (0.66 - 2.69)	0,533	1.59 (0.82 - 3.08)	0,407	1.83 (0.97 - 3.46)	0,129
SM.a.C39.1	1.97 (0.95 - 4.1)	0,163	1.78 (0.89 - 3.56)	0,303	1.94 (1 - 3.76)	0,109
SM.a.C39.2	1.11 (0.53 - 2.32)	0,835	1.1 (0.55 - 2.2)	0,872	1.67 (0.86 - 3.23)	0,204
SM.a.C40.2	2 (0.99 - 4.07)	0,143	2.11 (1.08 - 4.13)	0,184	2.27 (1.2 - 4.32)	0,064
SM.a.C40.5	2.03 (1.02 - 4.05)	0,124	1.91 (0.98 - 3.7)	0,237	3.38 (1.81 - 6.32)	0,009*
SM.a.C41.1	1.95 (0.97 - 3.92)	0,148	1.89 (0.98 - 3.66)	0,237	2.14 (1.15 - 4)	0,067
SM.a.C41.2	1.57 (0.77 - 3.18)	0,320	1.48 (0.76 - 2.9)	0,461	2.12 (1.12 - 4)	0,071
SM.a.C42.1	2.03 (1.02 - 4.04)	0,123	1.26 (0.65 - 2.43)	0,705	1.7 (0.91 - 3.18)	0,163
SM.a.C42.2	2.16 (1.06 - 4.43)	0,109	1.24 (0.63 - 2.47)	0,729	2.14 (1.11 - 4.1)	0,073
SM.a.C42.3	2.11 (1.03 - 4.32)	0,117	1.57 (0.79 - 3.11)	0,419	2.15 (1.12 - 4.14)	0,071
SM.a.C42.4	2.67 (1.34 - 5.31)	0,038	2.78 (1.44 - 5.37)	0,049	2.73 (1.45 - 5.13)	0,032*
SM.a.C42.6	1.99 (0.97 - 4.07)	0,148	2.02 (1.02 - 4)	0,217	2.24 (1.17 - 4.32)	0,066
SM.a.C43.1	2.34 (1.15 - 4.75)	0,085	1.18 (0.6 - 2.34)	0,772	1.56 (0.82 - 2.99)	0,248
SM.a.C43.2	2.17 (1.07 - 4.37)	0,103	1.84 (0.94 - 3.62)	0,250	1.46 (0.77 - 2.77)	0,309
SM.a.C44.6	1.84 (0.9 - 3.73)	0,193	1.4 (0.71 - 2.75)	0,552	2.3 (1.21 - 4.38)	0,064
SM.e.C36.2	1.63 (0.78 - 3.42)	0,306	1.27 (0.63 - 2.56)	0,709	1.54 (0.78 - 3.02)	0,283
SM.e.C38.3	1.26 (0.63 - 2.52)	0,605	1.32 (0.68 - 2.57)	0,626	2.12 (1.13 - 4)	0,071
SM.e.C40.5	1.7 (0.86 - 3.36)	0,221	1.56 (0.81 - 3.01)	0,414	2.14 (1.15 - 3.97)	0,067
Carn	1.5 (0.74 - 3.04)	0,377	1.28 (0.65 - 2.49)	0,691	2.26 (1.2 - 4.26)	0,064
Carn.a.C10.0	2.22 (1.12 - 4.42)	0,091	1.24 (0.63 - 2.41)	0,729	1.65 (0.87 - 3.1)	0,199
Carn.a.C10.1	1.64 (0.82 - 3.31)	0,276	1.03 (0.53 - 2.03)	0,939	1.91 (1.01 - 3.62)	0,109
Carn.a.C12.0	1.72 (0.86 - 3.41)	0,221	1.17 (0.61 - 2.28)	0,774	1.93 (1.03 - 3.63)	0,102
Carn.a.C14.1	1.39 (0.7 - 2.76)	0,464	0.84 (0.43 - 1.63)	0,769	1.88 (1 - 3.52)	0,109
Carn.a.C14.2	1.09 (0.55 - 2.16)	0,849	0.91 (0.47 - 1.75)	0,872	1.79 (0.96 - 3.34)	0,131
Carn.a.C15.0	1.19 (0.6 - 2.37)	0,694	1.03 (0.54 - 1.99)	0,939	1.5 (0.8 - 2.81)	0,277
Carn.a.C16.0	1.36 (0.69 - 2.68)	0,488	1.51 (0.79 - 2.92)	0,433	1.44 (0.77 - 2.69)	0,316
Carn.a.C16.0.Oxo	1.16 (0.58 - 2.33)	0,758	1 (0.51 - 1.97)	0,998	1.55 (0.82 - 2.96)	0,248

Carn.a.C16.1	1.03 (0.52 - 2.06)	0,956	1.01 (0.52 - 1.96)	0,979	1.89 (1 - 3.56)	0,109
Carn.a.C16.2	0.76 (0.39 - 1.5)	0,537	0.84 (0.44 - 1.61)	0,769	1.59 (0.86 - 2.96)	0,212
Carn.a.C18.0	0.89 (0.45 - 1.76)	0,804	0.66 (0.35 - 1.28)	0,438	1.05 (0.56 - 1.96)	0,889
Carn.a.C18.1	1.39 (0.69 - 2.77)	0,465	1.07 (0.55 - 2.09)	0,909	2.09 (1.11 - 3.94)	0,072
Carn.a.C18.2	1.27 (0.65 - 2.51)	0,585	1.09 (0.57 - 2.11)	0,872	1.73 (0.93 - 3.22)	0,151
Carn.a.C18.2.OH	1.07 (0.55 - 2.11)	0,883	0.92 (0.48 - 1.75)	0,872	1.19 (0.64 - 2.22)	0,619
Carn.a.C2.0	2.36 (1.18 - 4.69)	0,074	1.58 (0.82 - 3.07)	0,407	3.16 (1.68 - 5.94)	0,009*
Carn.a.C20.0	1.56 (0.78 - 3.11)	0,313	1.84 (0.96 - 3.55)	0,237	1.31 (0.7 - 2.46)	0,459
Carn.a.C20.1	1.29 (0.65 - 2.53)	0,566	0.98 (0.51 - 1.87)	0,971	1.59 (0.86 - 2.95)	0,212
Carn.a.C20.3	1.13 (0.58 - 2.22)	0,790	1.14 (0.6 - 2.16)	0,814	1.22 (0.66 - 2.26)	0,569
Carn.a.C20.4	1.4 (0.71 - 2.76)	0,457	1.18 (0.62 - 2.27)	0,769	1.46 (0.78 - 2.72)	0,300
Carn.a.C3.0	1.79 (0.9 - 3.53)	0,195	1.31 (0.68 - 2.52)	0,635	1.79 (0.96 - 3.33)	0,131
Carn.a.C3.0.DC	1.03 (0.51 - 2.05)	0,958	0.89 (0.45 - 1.73)	0,838	1.27 (0.67 - 2.4)	0,510
Carn.a.C4.0	1.84 (0.94 - 3.63)	0,169	1.37 (0.71 - 2.64)	0,553	1.75 (0.94 - 3.25)	0,142
Carn.a.C5.0	1.92 (0.97 - 3.79)	0,148	1.64 (0.85 - 3.15)	0,362	1.85 (0.99 - 3.45)	0,116
Carn.a.C6.0	2.27 (1.14 - 4.53)	0,087	1.26 (0.65 - 2.47)	0,705	1.54 (0.82 - 2.92)	0,248
Carn.a.C6.0.OH	1.39 (0.69 - 2.8)	0,465	1.16 (0.59 - 2.27)	0,793	1.75 (0.93 - 3.32)	0,152
Carn.a.C8.0	1.8 (0.9 - 3.6)	0,195	1.21 (0.62 - 2.35)	0,769	1.31 (0.69 - 2.48)	0,459
Carn.a.C8.1	1.64 (0.81 - 3.33)	0,284	1.63 (0.83 - 3.22)	0,402	1.96 (1.03 - 3.74)	0,102
Carn.a.C9.0	2.19 (1.11 - 4.35)	0,091	1.1 (0.57 - 2.14)	0,872	1.42 (0.76 - 2.67)	0,335
Asn/Asp	0.35 (0.18 - 0.68)	0,032	0.54 (0.28 - 1.04)	0,237	0.5 (0.27 - 0.94)	0,092
Gln/Glu	0.57 (0.29 - 1.14)	0,213	0.49 (0.25 - 0.95)	0,203	0.31 (0.16 - 0.58)	0,009*
NEFA18.1/NEFA18.0	1 (0.5 - 2.02)	0,998	0.83 (0.42 - 1.63)	0,769	1.53 (0.8 - 2.92)	0,270
NEFA16.1/NEFA16.0	1.07 (0.52 - 2.19)	0,903	1.27 (0.64 - 2.54)	0,705	1.23 (0.63 - 2.39)	0,574
PC.aa/PC.ae	1.27 (0.63 - 2.56)	0,591	1.49 (0.76 - 2.93)	0,461	1.41 (0.74 - 2.69)	0,355
Lyso.PC.a/PC.aa	0.71 (0.35 - 1.44)	0,464	1.19 (0.61 - 2.32)	0,769	1.3 (0.68 - 2.48)	0,477
(lyso.PC.a.C16.0 + lyso.PC.a.C18.0)/ PC.aa	0.65 (0.32 - 1.33)	0,356	1.46 (0.74 - 2.88)	0,478	1.55 (0.81 - 2.97)	0,260
(lyso.PC.a.C18.1 + lyso.PC.a.C18.2)	0.75 (0.37 - 1.51)	0,533	0.64 (0.33 - 1.24)	0,415	0.78 (0.41 - 1.46)	0,486
Carn.a.C.16.0/ free Carn	1.1 (0.55 - 2.2)	0,837	1.34 (0.69 - 2.59)	0,609	0.89 (0.47 - 1.68)	0,743
Carn.a.C2.0/ Carn.a.C16.0	1.43 (0.72 - 2.82)	0,430	0.95 (0.49 - 1.84)	0,927	1.47 (0.78 - 2.76)	0,300

Values represent absolute differences in blood pressure (95% confidence interval) and corresponding p-values from linear regression models that reflect the difference in blood pressure (mmHg) per SDS increase in maternal early-pregnancy metabolite concentrations (μmol/L) or metabolite ratio. Model includes gestational age at time of measurement, age, parity, pre-pregnancy body mass index, educational level, smoking and folic acid supplementation. AA amino acids, NEFA non-esterified fatty acids, PC.aa diacyl-phosphatidylcholines, PC.ae acyl-alkyl-phosphatidylcholines, lyso.PC.a acyl-lysophosphatidylcholines, lyso.PC.e alkyl-lysophosphatidylcholines, Carn.a acyl-carnitines, SM sphingomyelins.
[a] p-value corrected for multiple hypothesis testing using Benjamin-Hochberg FDR correction.
*****Statistically significant

Table S6. Associations of early-pregnancy individual metabolites with systolic blood pressure in early-, mid- and late pregnancy. Full model, additional adjustment for family history of hypertensive disorders.

	**Differences in systolic blood pressure in mmHg (95% confidence interval)**					
**Metabolite**	**Early pregnancy** N = 803	**P-value**[a]	**Mid pregnancy** N = 793	**P-value**[a]	**Late pregnancy** N = 800	**P-value**[a]
Ala	3.01 (1.2 - 7.51)	0,095	1.97 (0.8 - 4.85)	0,378	2.4 (1.09 - 5.3)	0,143
Arg	4.92 (1.98 - 12.25)	0,009*	2.47 (1 - 6.12)	0,231	3.19 (1.45 - 7)	0,073
Asn	3.11 (1.24 - 7.81)	0,082	1.07 (0.43 - 2.63)	0,962	2.92 (1.33 - 6.43)	0,077
Asp	1.92 (0.76 - 4.83)	0,362	2.7 (1.1 - 6.65)	0,186	2.98 (1.35 - 6.56)	0,077
Cit	1.35 (0.51 - 3.57)	0,726	0.87 (0.34 - 2.23)	0,907	1.16 (0.5 - 2.67)	0,885
Gln	2.19 (0.88 - 5.43)	0,261	1.01 (0.41 - 2.45)	0,986	0.93 (0.42 - 2.05)	0,950
Glu	1.74 (0.69 - 4.39)	0,436	3.41 (1.39 - 8.37)	0,086	3.03 (1.37 - 6.68)	0,077
Gly	0.82 (0.33 - 2.04)	0,829	0.97 (0.4 - 2.35)	0,983	1.3 (0.59 - 2.83)	0,797
His	3.69 (1.45 - 9.4)	0,047*	1.58 (0.63 - 3.93)	0,566	3.1 (1.4 - 6.86)	0,077
Ile	3.23 (1.28 - 8.16)	0,074	1.31 (0.53 - 3.25)	0,778	1.61 (0.72 - 3.56)	0,498
Leu	2.78 (1.11 - 6.95)	0,128	1.29 (0.53 - 3.15)	0,778	2.05 (0.94 - 4.49)	0,262
Lys	0.78 (0.31 - 1.95)	0,771	1.35 (0.55 - 3.35)	0,717	2.9 (1.32 - 6.38)	0,077
Met	2.89 (1.16 - 7.23)	0,110	1.46 (0.59 - 3.6)	0,625	1.78 (0.81 - 3.92)	0,376
Orn	1.19 (0.47 - 3.05)	0,838	1.23 (0.5 - 3.03)	0,808	1.94 (0.88 - 4.3)	0,318
Phe	5.55 (2.26 - 13.59)	0,005*	2.43 (1.01 - 5.88)	0,225	2.29 (1.05 - 4.97)	0,159
Pro	2.29 (0.92 - 5.71)	0,234	1.83 (0.75 - 4.45)	0,418	1.49 (0.68 - 3.27)	0,581
Trp	7.33 (2.95 - 18.19)	0,001*	2.81 (1.15 - 6.88)	0,175	2.89 (1.32 - 6.31)	0,077
Ser	2.05 (0.82 - 5.12)	0,301	1.14 (0.47 - 2.76)	0,910	2.4 (1.1 - 5.24)	0,143
Thr	1.57 (0.61 - 4.04)	0,541	1.56 (0.62 - 3.93)	0,579	2.17 (0.98 - 4.83)	0,221
Tyr	5.34 (2.17 - 13.19)	0,005*	2.06 (0.85 - 5.01)	0,338	2.65 (1.22 - 5.76)	0,108
Val	5.35 (2.15 - 13.32)	0,006*	1.89 (0.78 - 4.59)	0,404	2.61 (1.19 - 5.7)	0,121
Cys	0.99 (0.38 - 2.55)	0,996	1.12 (0.45 - 2.83)	0,910	1.49 (0.66 - 3.36)	0,614
NEFA_14_0	1.64 (0.66 - 4.1)	0,496	1.49 (0.61 - 3.61)	0,605	0.87 (0.4 - 1.92)	0,885
NEFA_14_1	1.05 (0.42 - 2.65)	0,968	1.29 (0.53 - 3.17)	0,778	1.02 (0.46 - 2.26)	0,982
NEFA_15_0	1.7 (0.68 - 4.22)	0,450	1.59 (0.66 - 3.84)	0,549	0.69 (0.32 - 1.51)	0,616
NEFA_16_0	1.83 (0.73 - 4.61)	0,396	1.8 (0.74 - 4.41)	0,422	0.89 (0.4 - 1.97)	0,903
NEFA_16_1	0.97 (0.38 - 2.45)	0,990	1.43 (0.58 - 3.52)	0,648	1.06 (0.48 - 2.35)	0,960
NEFA_16_2	1.35 (0.53 - 3.41)	0,714	1.47 (0.6 - 3.61)	0,622	0.91 (0.41 - 2.01)	0,928
NEFA_17_0	1.48 (0.6 - 3.67)	0,585	1.39 (0.58 - 3.34)	0,689	0.93 (0.42 - 2.02)	0,947
NEFA_17_1	1.59 (0.64 - 3.95)	0,523	1.45 (0.6 - 3.5)	0,622	0.88 (0.4 - 1.92)	0,885
NEFA_17_2	1.73 (0.69 - 4.32)	0,436	1.1 (0.45 - 2.69)	0,928	0.82 (0.37 - 1.81)	0,871
NEFA_18_0	1.54 (0.61 - 3.91)	0,545	1.24 (0.5 - 3.05)	0,808	0.82 (0.37 - 1.81)	0,864
NEFA_18_1	1.38 (0.55 - 3.46)	0,679	1.38 (0.57 - 3.36)	0,694	1.09 (0.5 - 2.41)	0,938
NEFA_18_2	1.58 (0.64 - 3.93)	0,523	1.12 (0.46 - 2.73)	0,910	0.72 (0.33 - 1.58)	0,683
NEFA_18_3	1.94 (0.78 - 4.82)	0,345	1.39 (0.57 - 3.36)	0,689	0.66 (0.3 - 1.44)	0,546
NEFA_19_1	1.26 (0.51 - 3.11)	0,797	1.08 (0.45 - 2.59)	0,954	0.84 (0.38 - 1.83)	0,871
NEFA_20_1	1.6 (0.64 - 4)	0,523	1.15 (0.47 - 2.81)	0,907	1.17 (0.53 - 2.58)	0,884
NEFA_20_2	1.25 (0.5 - 3.12)	0,797	1.29 (0.53 - 3.14)	0,778	1.17 (0.53 - 2.59)	0,884
NEFA_20_3	2.73 (1.06 - 7.04)	0,154	3.11 (1.25 - 7.77)	0,141	1.81 (0.8 - 4.08)	0,376
NEFA_20_4	2.13 (0.85 - 5.32)	0,286	1.95 (0.8 - 4.76)	0,378	1.67 (0.76 - 3.66)	0,449

NEFA_20_5	2.4 (0.95 - 6.06)	0,202	1.5 (0.61 - 3.69)	0,602	1.68 (0.76 - 3.69)	0,449
NEFA_22_3	1.48 (0.59 - 3.73)	0,588	2.61 (1.07 - 6.36)	0,196	2.48 (1.12 - 5.5)	0,143
NEFA_22_4	2.57 (1 - 6.6)	0,173	2.9 (1.17 - 7.21)	0,175	2.06 (0.92 - 4.64)	0,281
NEFA_22_5	2.25 (0.89 - 5.66)	0,255	1.83 (0.75 - 4.49)	0,418	1.63 (0.73 - 3.61)	0,489
NEFA_22_6	1.75 (0.69 - 4.41)	0,436	1.28 (0.52 - 3.16)	0,784	1.26 (0.57 - 2.8)	0,828
NEFA_24_0	2.21 (0.9 - 5.47)	0,255	2 (0.82 - 4.85)	0,365	0.85 (0.39 - 1.84)	0,882
NEFA_24_1	1.03 (0.41 - 2.56)	0,990	0.72 (0.3 - 1.76)	0,694	0.95 (0.43 - 2.07)	0,960
NEFA_24_2	1 (0.39 - 2.55)	0,996	1.45 (0.59 - 3.56)	0,629	1.44 (0.65 - 3.19)	0,627
NEFA_24_4	3.19 (1.28 - 7.95)	0,074	2.84 (1.17 - 6.89)	0,175	2.6 (1.19 - 5.7)	0,121
NEFA_24_5	3.58 (1.42 - 8.99)	0,047*	2.52 (1.03 - 6.16)	0,216	3.83 (1.74 - 8.43)	0,022*
NEFA_26_0	3.58 (1.45 - 8.81)	0,043*	1.76 (0.73 - 4.25)	0,439	1.58 (0.73 - 3.44)	0,504
NEFA_26_1	1.95 (0.78 - 4.89)	0,345	2.47 (1.01 - 6.07)	0,225	1.82 (0.83 - 4.01)	0,359
NEFA_26_2	1.38 (0.53 - 3.55)	0,698	1.72 (0.69 - 4.27)	0,471	1.35 (0.6 - 3.02)	0,732
lyso.PC.a.C14.0	14.01 (5.58 - 35.18)	0,000*	8.56 (3.45 - 21.29)	0,001*	5.59 (2.51 - 12.47)	0,006*
lyso.PC.a.C16.0	3.55 (1.38 - 9.12)	0,055	4.8 (1.92 - 11.99)	0,030*	2.55 (1.13 - 5.74)	0,143
lyso.PC.a.C16.1	5.17 (1.97 - 13.58)	0,011*	6.16 (2.43 - 15.62)	0,010*	4.85 (2.13 - 11.01)	0,012*
lyso.PC.a.C18.0	1.88 (0.73 - 4.85)	0,389	3.68 (1.48 - 9.18)	0,076	1.69 (0.76 - 3.79)	0,449
lyso.PC.a.C18.1	5.25 (2.1 - 13.12)	0,006*	2.91 (1.18 - 7.16)	0,175	1.86 (0.84 - 4.1)	0,343
lyso.PC.a.C18.2	5.09 (2.03 - 12.79)	0,008*	2.07 (0.85 - 5.05)	0,338	1.28 (0.58 - 2.81)	0,815
lyso.PC.a.C18.3	5.83 (2.41 - 14.14)	0,003*	2.12 (0.89 - 5.08)	0,309	2.8 (1.3 - 6.03)	0,078
lyso.PC.a.C20.3	8.42 (3.39 - 20.92)	0,000*	6.98 (2.86 - 17.05)	0,002*	4.99 (2.27 - 10.94)	0,007*
lyso.PC.a.C20.4	5.6 (2.23 - 14.09)	0,005*	4.74 (1.94 - 11.59)	0,030*	2.39 (1.09 - 5.28)	0,143
lyso.PC.a.C20.5	2.54 (1 - 6.41)	0,173	1.26 (0.52 - 3.06)	0,786	2.23 (1.02 - 4.86)	0,179
lyso.PC.a.C22.6	2.78 (1.1 - 7)	0,129	1.61 (0.66 - 3.94)	0,542	1.21 (0.55 - 2.67)	0,871
lyso.PC.e.C16.0	2.69 (1.06 - 6.83)	0,151	2.84 (1.15 - 7.03)	0,175	1.88 (0.84 - 4.21)	0,337
lyso.PC.e.C18.0	0.86 (0.34 - 2.15)	0,852	1.7 (0.69 - 4.14)	0,471	1.23 (0.56 - 2.71)	0,864
lyso.PC.e.C18.1	2.57 (0.99 - 6.67)	0,184	1.3 (0.52 - 3.29)	0,778	1.57 (0.69 - 3.54)	0,537
PC.aa.C30.0	6.31 (2.54 - 15.68)	0,003*	3.12 (1.27 - 7.64)	0,141	2.44 (1.11 - 5.37)	0,143
PC.aa.C30.3	1.14 (0.46 - 2.87)	0,879	1.8 (0.74 - 4.39)	0,418	0.87 (0.39 - 1.92)	0,885
PC.aa.C32.0	3.92 (1.54 - 9.94)	0,038*	2.67 (1.08 - 6.6)	0,189	1.84 (0.82 - 4.11)	0,359
PC.aa.C32.1	5.78 (2.27 - 14.72)	0,005*	3.98 (1.61 - 9.86)	0,060	4.19 (1.89 - 9.3)	0,017*
PC.aa.C32.2	6.4 (2.55 - 16.06)	0,003*	2.77 (1.13 - 6.78)	0,176	2.51 (1.14 - 5.53)	0,143
PC.aa.C32.3	1.4 (0.54 - 3.65)	0,679	1.79 (0.71 - 4.51)	0,442	1.16 (0.52 - 2.62)	0,885
PC.aa.C34.1	3.26 (1.29 - 8.26)	0,074	3.39 (1.39 - 8.27)	0,086	2.99 (1.35 - 6.6)	0,077
PC.aa.C34.2	2.24 (0.88 - 5.72)	0,261	1.88 (0.77 - 4.62)	0,411	1.46 (0.66 - 3.24)	0,616
PC.aa.C34.3	3.26 (1.29 - 8.28)	0,074	2.02 (0.82 - 4.98)	0,365	1.92 (0.87 - 4.28)	0,322
PC.aa.C34.4	8.03 (3.28 - 19.67)	0,000*	3.83 (1.58 - 9.27)	0,060	2.77 (1.27 - 6.05)	0,093
PC.aa.C34.5	4.4 (1.71 - 11.32)	0,023*	1.87 (0.74 - 4.72)	0,418	1.79 (0.8 - 4.04)	0,378
PC.aa.C36.0	1.21 (0.47 - 3.12)	0,837	1.69 (0.67 - 4.28)	0,498	1.17 (0.52 - 2.64)	0,884
PC.aa.C36.1	4.15 (1.66 - 10.34)	0,024*	2.41 (0.98 - 5.9)	0,231	2.31 (1.05 - 5.1)	0,161
PC.aa.C36.2	2.33 (0.91 - 5.96)	0,238	1.78 (0.72 - 4.41)	0,439	1.35 (0.61 - 3.02)	0,732
PC.aa.C36.3	3.25 (1.27 - 8.31)	0,077	2.76 (1.11 - 6.84)	0,182	2.58 (1.16 - 5.72)	0,134
PC.aa.C36.4	3.47 (1.38 - 8.73)	0,055	3.7 (1.51 - 9.07)	0,076	2.47 (1.11 - 5.48)	0,143

PC.aa.C36.5	4.62 (1.84 - 11.59)	0,014*	1.82 (0.74 - 4.5)	0,418	3.06 (1.38 - 6.74)	0,077
PC.aa.C36.6	4.6 (1.82 - 11.68)	0,015*	2.3 (0.92 - 5.72)	0,279	2.98 (1.34 - 6.63)	0,077
PC.aa.C38.0	1.16 (0.44 - 3.01)	0,871	1.1 (0.43 - 2.81)	0,936	0.98 (0.43 - 2.21)	0,977
PC.aa.C38.2	2.69 (1.04 - 6.93)	0,158	2.73 (1.09 - 6.85)	0,186	2.44 (1.09 - 5.49)	0,143
PC.aa.C38.3	3.97 (1.54 - 10.23)	0,038*	4.82 (1.91 - 12.14)	0,030*	4.18 (1.86 - 9.41)	0,017*
PC.aa.C38.4	2.44 (0.96 - 6.17)	0,196	3.66 (1.48 - 9.03)	0,076	2.43 (1.09 - 5.43)	0,143
PC.aa.C38.5	2.6 (1.04 - 6.5)	0,158	2.67 (1.1 - 6.51)	0,186	3.39 (1.54 - 7.43)	0,055
PC.aa.C38.6	1.51 (0.57 - 3.96)	0,588	1.53 (0.6 - 3.91)	0,602	1.87 (0.82 - 4.26)	0,359
PC.aa.C40.0	1.76 (0.68 - 4.59)	0,441	0.97 (0.38 - 2.48)	0,983	0.91 (0.4 - 2.07)	0,940
PC.aa.C40.1	1.77 (0.69 - 4.51)	0,436	2 (0.81 - 4.98)	0,378	1.15 (0.52 - 2.55)	0,885
PC.aa.C40.2	3.9 (1.55 - 9.81)	0,038*	1.74 (0.71 - 4.27)	0,456	2.37 (1.08 - 5.23)	0,144
PC.aa.C40.3	2.54 (1.01 - 6.39)	0,173	1.91 (0.77 - 4.72)	0,404	2.43 (1.1 - 5.39)	0,143
PC.aa.C40.4	3.78 (1.5 - 9.52)	0,040*	4.47 (1.82 - 10.97)	0,032*	4.1 (1.86 - 9.03)	0,017*
PC.aa.C40.5	2.12 (0.86 - 5.25)	0,285	3.59 (1.49 - 8.65)	0,076	3.22 (1.47 - 7.02)	0,070
PC.aa.C40.6	2.11 (0.82 - 5.44)	0,301	2.01 (0.8 - 5.06)	0,378	2.43 (1.08 - 5.48)	0,144
PC.aa.C42.0	1.12 (0.45 - 2.82)	0,889	1.04 (0.42 - 2.57)	0,983	1.6 (0.73 - 3.51)	0,494
PC.aa.C42.5	1.4 (0.56 - 3.51)	0,669	0.97 (0.4 - 2.38)	0,983	1.29 (0.59 - 2.83)	0,800
PC.aa.C43.6	0.86 (0.34 - 2.18)	0,852	1.29 (0.52 - 3.21)	0,778	1.2 (0.54 - 2.66)	0,871
PC.aa.C44.12	1.31 (0.51 - 3.36)	0,756	1.77 (0.71 - 4.41)	0,448	1.6 (0.71 - 3.59)	0,504
PC.ae.C30.0	3.73 (1.49 - 9.38)	0,040*	2.34 (0.95 - 5.75)	0,255	1.92 (0.87 - 4.23)	0,322
PC.ae.C32.0	2.6 (1.03 - 6.55)	0,165	2.57 (1.05 - 6.28)	0,205	1.96 (0.89 - 4.33)	0,302
PC.ae.C32.1	1.97 (0.77 - 5.07)	0,347	2.55 (1.03 - 6.33)	0,216	1.2 (0.53 - 2.69)	0,871
PC.ae.C32.2	1.64 (0.65 - 4.13)	0,497	1.46 (0.6 - 3.57)	0,622	0.78 (0.35 - 1.72)	0,815
PC.ae.C34.0	2.83 (1.12 - 7.12)	0,126	2.57 (1.05 - 6.28)	0,205	1.73 (0.78 - 3.82)	0,406
PC.ae.C34.1	2.24 (0.89 - 5.68)	0,258	2.11 (0.86 - 5.19)	0,327	1.86 (0.84 - 4.11)	0,345
PC.ae.C34.2	1.6 (0.62 - 4.14)	0,527	1.5 (0.6 - 3.76)	0,605	1.13 (0.5 - 2.56)	0,897
PC.ae.C34.3	1.59 (0.64 - 3.96)	0,523	1.58 (0.65 - 3.82)	0,550	0.83 (0.38 - 1.82)	0,871
PC.ae.C34.4	1.79 (0.7 - 4.56)	0,424	1.36 (0.55 - 3.35)	0,717	1.45 (0.65 - 3.23)	0,623
PC.ae.C36.0	0.99 (0.39 - 2.52)	0,996	1.82 (0.74 - 4.47)	0,418	1.12 (0.5 - 2.48)	0,919
PC.ae.C36.1	1.83 (0.72 - 4.63)	0,396	2.44 (0.99 - 6.03)	0,231	2.54 (1.15 - 5.62)	0,140
PC.ae.C36.2	1.08 (0.41 - 2.8)	0,948	1.02 (0.41 - 2.54)	0,986	1.04 (0.46 - 2.33)	0,974
PC.ae.C36.3	1.82 (0.72 - 4.62)	0,404	1.74 (0.7 - 4.32)	0,456	1.79 (0.81 - 3.98)	0,375
PC.ae.C36.4	2.45 (0.98 - 6.09)	0,186	4.13 (1.7 - 10.03)	0,045*	2.15 (0.98 - 4.71)	0,220
PC.ae.C36.5	1.96 (0.79 - 4.87)	0,341	2.18 (0.9 - 5.3)	0,298	1.4 (0.64 - 3.05)	0,663
PC.ae.C38.0	1.6 (0.62 - 4.15)	0,527	1.02 (0.4 - 2.56)	0,986	1.55 (0.69 - 3.51)	0,543
PC.ae.C38.2	1.92 (0.74 - 5.01)	0,376	2.3 (0.91 - 5.82)	0,282	1.28 (0.56 - 2.9)	0,817
PC.ae.C38.3	2.48 (0.97 - 6.34)	0,196	3.57 (1.43 - 8.91)	0,084	3.1 (1.39 - 6.93)	0,077
PC.ae.C38.4	1.89 (0.76 - 4.69)	0,362	3.04 (1.26 - 7.37)	0,141	1.9 (0.87 - 4.17)	0,322
PC.ae.C38.5	1.62 (0.65 - 4.06)	0,503	2.22 (0.91 - 5.43)	0,286	1.53 (0.7 - 3.38)	0,543
PC.ae.C38.6	1.68 (0.66 - 4.28)	0,480	1.74 (0.7 - 4.36)	0,461	1.56 (0.7 - 3.48)	0,534
PC.ae.C40.0	1.73 (0.65 - 4.59)	0,474	1.13 (0.44 - 2.91)	0,910	1.45 (0.62 - 3.35)	0,653
PC.ae.C40.1	2.87 (1.09 - 7.52)	0,135	1.3 (0.51 - 3.34)	0,781	1.69 (0.74 - 3.85)	0,462
PC.ae.C40.2	1.19 (0.46 - 3.06)	0,838	0.95 (0.38 - 2.38)	0,968	2.09 (0.94 - 4.63)	0,262
PC.ae.C40.3	2.02 (0.78 - 5.22)	0,341	2.22 (0.88 - 5.6)	0,309	1.61 (0.71 - 3.63)	0,504

PC.ae.C40.4	2.16 (0.85 - 5.47)	0,285	3.51 (1.42 - 8.65)	0,084	2.04 (0.92 - 4.56)	0,281
PC.ae.C40.5	1.12 (0.44 - 2.85)	0,889	1.53 (0.62 - 3.8)	0,586	1.59 (0.71 - 3.56)	0,504
PC.ae.C40.6	1 (0.39 - 2.58)	0,996	1.21 (0.48 - 3.03)	0,842	1.46 (0.65 - 3.3)	0,616
PC.ae.C42.1	2.06 (0.79 - 5.37)	0,333	2.18 (0.86 - 5.55)	0,327	1.54 (0.68 - 3.5)	0,556
PC.ae.C42.3	0.84 (0.32 - 2.2)	0,846	0.63 (0.25 - 1.59)	0,566	1.15 (0.51 - 2.61)	0,885
PC.ae.C42.4	2.14 (0.84 - 5.45)	0,288	2.16 (0.87 - 5.34)	0,321	1.91 (0.86 - 4.25)	0,329
PC.ae.C42.5	1.22 (0.48 - 3.11)	0,829	1.72 (0.69 - 4.26)	0,470	1.77 (0.79 - 3.95)	0,389
PC.ae.C42.6	0.99 (0.39 - 2.53)	0,996	0.93 (0.37 - 2.33)	0,962	1.14 (0.51 - 2.53)	0,892
SM.a.C30.1	2.2 (0.87 - 5.55)	0,269	2.48 (1.01 - 6.08)	0,225	1.88 (0.86 - 4.11)	0,331
SM.a.C32.1	1.81 (0.71 - 4.61)	0,408	2.39 (0.97 - 5.87)	0,243	1.99 (0.9 - 4.41)	0,302
SM.a.C32.2	1.87 (0.71 - 4.95)	0,403	3.19 (1.25 - 8.14)	0,141	3.21 (1.42 - 7.25)	0,077
SM.a.C33.1	1.01 (0.39 - 2.6)	0,996	1.53 (0.62 - 3.79)	0,586	1.27 (0.57 - 2.84)	0,817
SM.a.C34.1	1.01 (0.39 - 2.63)	0,996	1.64 (0.66 - 4.11)	0,529	1.31 (0.58 - 2.96)	0,797
SM.a.C34.2	1.13 (0.42 - 3.1)	0,889	2.03 (0.77 - 5.36)	0,402	1.71 (0.73 - 3.98)	0,462
SM.a.C35.0	0.77 (0.3 - 1.96)	0,771	0.88 (0.36 - 2.18)	0,910	1.26 (0.56 - 2.8)	0,828
SM.a.C35.1	0.38 (0.14 - 0.99)	0,173	0.84 (0.33 - 2.11)	0,854	0.74 (0.33 - 1.67)	0,742
SM.a.C36.1	0.79 (0.3 - 2.09)	0,797	1.54 (0.6 - 3.94)	0,602	1.07 (0.47 - 2.47)	0,950
SM.a.C36.2	0.44 (0.16 - 1.22)	0,297	1.22 (0.46 - 3.23)	0,842	0.91 (0.39 - 2.15)	0,941
SM.a.C36.3	0.77 (0.28 - 2.08)	0,786	0.89 (0.34 - 2.32)	0,910	1.01 (0.43 - 2.33)	0,996
SM.a.C37.1	1.2 (0.46 - 3.1)	0,838	1.27 (0.51 - 3.18)	0,786	2.15 (0.97 - 4.77)	0,226
SM.a.C38.2	1.26 (0.49 - 3.28)	0,797	1.89 (0.76 - 4.67)	0,411	1.28 (0.57 - 2.86)	0,815
SM.a.C38.3	1.24 (0.49 - 3.16)	0,810	1.5 (0.61 - 3.66)	0,602	0.98 (0.44 - 2.16)	0,977
SM.a.C39.1	2.16 (0.82 - 5.73)	0,301	2.25 (0.88 - 5.73)	0,309	2.32 (1.02 - 5.27)	0,179
SM.a.C39.2	0.73 (0.28 - 1.91)	0,707	0.76 (0.3 - 1.91)	0,776	1.22 (0.54 - 2.73)	0,871
SM.a.C40.2	1.88 (0.73 - 4.83)	0,387	2.84 (1.15 - 7.02)	0,175	2.04 (0.92 - 4.53)	0,281
SM.a.C40.5	3.36 (1.34 - 8.43)	0,061	2.02 (0.82 - 4.96)	0,365	4.01 (1.84 - 8.76)	0,017*
SM.a.C41.1	3.04 (1.2 - 7.68)	0,095	2 (0.82 - 4.88)	0,365	1.79 (0.82 - 3.92)	0,370
SM.a.C41.2	1.31 (0.51 - 3.35)	0,762	1.02 (0.41 - 2.52)	0,986	1.97 (0.89 - 4.35)	0,302
SM.a.C42.1	1.56 (0.62 - 3.95)	0,536	1.09 (0.44 - 2.67)	0,948	1.34 (0.61 - 2.96)	0,732
SM.a.C42.2	1.13 (0.43 - 2.95)	0,889	0.88 (0.35 - 2.22)	0,910	1.48 (0.65 - 3.35)	0,616
SM.a.C42.3	0.99 (0.38 - 2.59)	0,996	1.56 (0.62 - 3.95)	0,579	1.59 (0.71 - 3.6)	0,510
SM.a.C42.4	2.42 (0.96 - 6.07)	0,196	2.79 (1.14 - 6.84)	0,175	1.98 (0.89 - 4.39)	0,302
SM.a.C42.6	1.96 (0.75 - 5.1)	0,362	1.96 (0.77 - 4.95)	0,402	1.96 (0.86 - 4.46)	0,322
SM.a.C43.1	1.57 (0.61 - 4.07)	0,541	1.06 (0.42 - 2.67)	0,966	1.05 (0.47 - 2.35)	0,969
SM.a.C43.2	1.21 (0.47 - 3.08)	0,836	1.24 (0.5 - 3.09)	0,808	0.96 (0.43 - 2.14)	0,974
SM.a.C44.6	1.42 (0.55 - 3.66)	0,666	1.65 (0.66 - 4.17)	0,526	1.9 (0.84 - 4.27)	0,337
SM.e.C36.2	0.47 (0.18 - 1.27)	0,333	0.73 (0.28 - 1.86)	0,717	0.9 (0.39 - 2.05)	0,921
SM.e.C38.3	0.94 (0.37 - 2.37)	0,960	1.26 (0.52 - 3.06)	0,792	0.99 (0.45 - 2.18)	0,996
SM.e.C40.5	1.48 (0.6 - 3.68)	0,588	1.85 (0.76 - 4.49)	0,418	1.11 (0.51 - 2.42)	0,921
Carn	1.12 (0.43 - 2.87)	0,897	1.88 (0.76 - 4.65)	0,411	2.43 (1.1 - 5.38)	0,143
Carn.a.C10.0	0.47 (0.19 - 1.18)	0,288	0.83 (0.34 - 2.05)	0,842	1.05 (0.47 - 2.32)	0,969
Carn.a.C10.1	0.54 (0.21 - 1.36)	0,389	0.59 (0.24 - 1.46)	0,473	0.81 (0.36 - 1.81)	0,864
Carn.a.C12.0	0.68 (0.27 - 1.69)	0,588	0.81 (0.33 - 1.99)	0,808	1.16 (0.53 - 2.57)	0,884
Carn.a.C14.1	0.6 (0.24 - 1.5)	0,480	0.64 (0.26 - 1.57)	0,567	1 (0.45 - 2.2)	0,998
Carn.a.C14.2	0.52 (0.21 - 1.28)	0,345	0.55 (0.23 - 1.34)	0,418	0.97 (0.44 - 2.12)	0,974

Carn.a.C15.0	0.84 (0.34 - 2.11)	0,838	0.96 (0.4 - 2.34)	0,983	1.67 (0.76 - 3.66)	0,449
Carn.a.C16.0	0.7 (0.28 - 1.73)	0,630	0.8 (0.33 - 1.96)	0,806	0.84 (0.38 - 1.86)	0,882
Carn.a.C16.0.Oxo	1.06 (0.42 - 2.7)	0,960	0.94 (0.38 - 2.33)	0,962	1 (0.44 - 2.24)	0,998
Carn.a.C16.1	0.34 (0.13 - 0.84)	0,096	0.55 (0.22 - 1.35)	0,418	1.17 (0.53 - 2.6)	0,884
Carn.a.C16.2	0.55 (0.22 - 1.37)	0,396	0.63 (0.26 - 1.54)	0,551	1.17 (0.54 - 2.56)	0,884
Carn.a.C18.0	1.17 (0.47 - 2.92)	0,848	0.5 (0.21 - 1.21)	0,365	0.98 (0.45 - 2.14)	0,977
Carn.a.C18.1	0.52 (0.21 - 1.32)	0,362	0.72 (0.29 - 1.78)	0,694	1.41 (0.64 - 3.14)	0,658
Carn.a.C18.2	0.66 (0.27 - 1.65)	0,567	0.9 (0.37 - 2.18)	0,910	0.93 (0.42 - 2.03)	0,949
Carn.a.C18.2.OH	0.9 (0.36 - 2.23)	0,898	0.86 (0.35 - 2.06)	0,872	1.19 (0.54 - 2.62)	0,871
Carn.a.C2.0	1.65 (0.65 - 4.15)	0,496	1.78 (0.73 - 4.36)	0,430	1.82 (0.82 - 4.02)	0,361
Carn.a.C20.0	1.4 (0.56 - 3.51)	0,669	1.84 (0.76 - 4.45)	0,418	0.83 (0.38 - 1.83)	0,871
Carn.a.C20.1	1.55 (0.63 - 3.82)	0,535	1.02 (0.42 - 2.44)	0,986	1.37 (0.63 - 2.97)	0,694
Carn.a.C20.3	0.88 (0.36 - 2.17)	0,883	1.23 (0.51 - 2.94)	0,808	0.69 (0.32 - 1.5)	0,616
Carn.a.C20.4	0.75 (0.3 - 1.85)	0,714	1.01 (0.42 - 2.44)	0,986	0.93 (0.43 - 2.05)	0,950
Carn.a.C3.0	2.84 (1.14 - 7.05)	0,115	2.23 (0.93 - 5.39)	0,279	2.26 (1.04 - 4.95)	0,169
Carn.a.C3.0.DC	0.79 (0.31 - 2)	0,797	1.06 (0.43 - 2.63)	0,966	1.62 (0.73 - 3.62)	0,490
Carn.a.C4.0	3.48 (1.41 - 8.57)	0,047*	2.32 (0.96 - 5.6)	0,255	1.87 (0.86 - 4.08)	0,330
Carn.a.C5.0	2.77 (1.11 - 6.89)	0,126	2.4 (0.98 - 5.83)	0,231	2.71 (1.24 - 5.95)	0,105
Carn.a.C6.0	0.83 (0.33 - 2.1)	0,836	1.06 (0.43 - 2.61)	0,968	1.06 (0.48 - 2.36)	0,960
Carn.a.C6.0.OH	0.57 (0.23 - 1.45)	0,436	1.21 (0.49 - 3.01)	0,842	1.74 (0.78 - 3.89)	0,406
Carn.a.C8.0	0.5 (0.2 - 1.27)	0,341	0.74 (0.3 - 1.82)	0,717	0.86 (0.39 - 1.9)	0,884
Carn.a.C8.1	0.82 (0.32 - 2.11)	0,829	1.01 (0.4 - 2.54)	0,986	0.68 (0.3 - 1.54)	0,616
Carn.a.C9.0	1.59 (0.63 - 3.98)	0,523	1.27 (0.52 - 3.12)	0,784	1.66 (0.75 - 3.65)	0,458
Asn/Asp	0.97 (0.4 - 2.37)	0,990	0.45 (0.19 - 1.08)	0,279	0.79 (0.36 - 1.71)	0,815
Gln/Glu	1.05 (0.42 - 2.63)	0,974	0.37 (0.15 - 0.9)	0,182	0.37 (0.17 - 0.82)	0,108
NEFA18.1/NEFA18.0	0.8 (0.31 - 2.03)	0,797	0.63 (0.25 - 1.56)	0,554	0.87 (0.39 - 1.95)	0,885
NEFA16.1/NEFA16.0	0.46 (0.18 - 1.2)	0,291	0.67 (0.27 - 1.7)	0,622	1.26 (0.55 - 2.88)	0,828
PC.aa/PC.ae	3.84 (1.53 - 9.67)	0,038*	2.3 (0.94 - 5.62)	0,269	2.41 (1.09 - 5.32)	0,143
Lyso.PC.a/PC.aa	1.43 (0.56 - 3.68)	0,656	1.91 (0.78 - 4.66)	0,402	1.03 (0.46 - 2.29)	0,977
(lyso.PC.a.C16.0 + lyso.PC.a.C18.0)/ PC.aa	0.91 (0.35 - 2.35)	0,912	2.02 (0.82 - 4.97)	0,365	1.06 (0.47 - 2.37)	0,960
(lyso.PC.a.C18.1 + lyso.PC.a.C18.2)	2.26 (0.89 - 5.71)	0,255	1.13 (0.47 - 2.75)	0,910	0.73 (0.33 - 1.61)	0,700
Carn.a.C.16.0/ free Carn	0.65 (0.26 - 1.63)	0,545	0.6 (0.25 - 1.44)	0,473	0.5 (0.23 - 1.11)	0,302
Carn.a.C2.0/ Carn.a.C16.0	1.96 (0.79 - 4.85)	0,341	1.92 (0.79 - 4.64)	0,393	1.75 (0.8 - 3.83)	0,388

Values represent absolute differences in blood pressure (95% confidence interval) and corresponding p-values from linear regression models that reflect the difference in blood pressure (mmHg) per SDS increase in maternal early-pregnancy metabolite concentrations (µmol/L) or metabolite ratio. Model includes gestational age at time of measurement, age, parity, pre-pregnancy body mass index, educational level, smoking, folic acid supplementation and family history of hypertension.
AA amino acids, NEFA non-esterified fatty acids, PC.aa diacyl-phosphatidylcholines, PC.ae acyl-alkyl-phosphatidylcholines, lyso.PC.a acyl-lysophosphatidylcholines, lyso.PC.e alkyl-lysophosphatidylcholines, Carn.a acyl-carnitines, SM sphingomyelins

[a] p-value corrected for multiple hypothesis testing using Benjamin-Hochberg FDR correction.

*****Statistically significant

Table S7. Associations of early-pregnancy individual metabolites with diastolic blood pressure in early-, mid- and late pregnancy. Full model, additional adjustment for family history of hypertensive disorders.

	**Differences in diastolic blood pressure in mmHg (95% confidence interval)**					
**Metabolite**	**Early pregnancy** N = 803	**P-value**[a]	**Mid pregnancy** N = 793	**P-value**[a]	**Late pregnancy** N = 800	**P-value**[a]
Ala	1.07 (0.54 - 2.12)	0,898	1.52 (0.78 - 2.95)	0,430	2.33 (1.25 - 4.34)	0,055
Arg	1.89 (0.96 - 3.75)	0,162	1.1 (0.57 - 2.15)	0,861	1.72 (0.92 - 3.21)	0,158
Asn	0.9 (0.45 - 1.79)	0,835	0.81 (0.42 - 1.58)	0,727	1.72 (0.92 - 3.22)	0,161
Asp	2.62 (1.31 - 5.23)	0,045*	1.73 (0.89 - 3.35)	0,305	3.03 (1.62 - 5.66)	0,014*
Cit	0.99 (0.48 - 2.05)	0,983	0.73 (0.37 - 1.47)	0,607	1.14 (0.59 - 2.21)	0,713
Gln	1.27 (0.64 - 2.51)	0,603	0.66 (0.34 - 1.27)	0,430	0.75 (0.4 - 1.42)	0,438
Glu	2.62 (1.32 - 5.2)	0,045*	1.99 (1.03 - 3.85)	0,203	3.63 (1.95 - 6.76)	0,008
Gly	0.74 (0.38 - 1.46)	0,498	0.87 (0.46 - 1.67)	0,804	1.53 (0.82 - 2.84)	0,253
His	0.98 (0.49 - 1.99)	0,975	0.82 (0.42 - 1.63)	0,757	1.96 (1.03 - 3.73)	0,104
Ile	0.67 (0.34 - 1.35)	0,390	0.83 (0.42 - 1.62)	0,757	1.47 (0.78 - 2.78)	0,307
Leu	0.77 (0.39 - 1.52)	0,553	0.82 (0.42 - 1.58)	0,739	1.66 (0.89 - 3.1)	0,182
Lys	0.45 (0.23 - 0.89)	0,091	1.16 (0.59 - 2.25)	0,804	2.14 (1.15 - 4)	0,064
Met	0.95 (0.48 - 1.89)	0,924	0.86 (0.44 - 1.68)	0,803	1.24 (0.66 - 2.32)	0,547
Orn	0.64 (0.32 - 1.29)	0,327	0.95 (0.49 - 1.86)	0,930	1.99 (1.06 - 3.75)	0,095
Phe	1.64 (0.84 - 3.22)	0,259	1.23 (0.64 - 2.36)	0,725	1.98 (1.07 - 3.66)	0,090
Pro	0.93 (0.47 - 1.83)	0,879	0.78 (0.41 - 1.5)	0,675	1.37 (0.74 - 2.55)	0,380
Trp	1.06 (0.53 - 2.11)	0,912	1 (0.52 - 1.94)	0,998	1.39 (0.75 - 2.59)	0,359
Ser	1.47 (0.74 - 2.92)	0,390	0.91 (0.47 - 1.76)	0,864	1.95 (1.04 - 3.63)	0,101
Thr	0.81 (0.4 - 1.64)	0,656	1.03 (0.52 - 2.04)	0,956	1.64 (0.87 - 3.1)	0,200
Tyr	1.25 (0.63 - 2.47)	0,622	0.92 (0.48 - 1.77)	0,873	1.56 (0.84 - 2.89)	0,239
Val	1.2 (0.6 - 2.37)	0,707	0.91 (0.47 - 1.76)	0,864	1.65 (0.88 - 3.07)	0,190
Cys	0.37 (0.18 - 0.74)	0,041*	0.71 (0.36 - 1.4)	0,535	1 (0.53 - 1.91)	0,995
NEFA_14_0	2.21 (1.12 - 4.36)	0,091	1.4 (0.73 - 2.69)	0,529	1.13 (0.61 - 2.1)	0,717
NEFA_14_1	3.29 (1.66 - 6.51)	0,015*	2.03 (1.05 - 3.92)	0,203	1.42 (0.75 - 2.67)	0,340
NEFA_15_0	2.21 (1.12 - 4.34)	0,091	1.5 (0.79 - 2.88)	0,430	0.88 (0.47 - 1.63)	0,705
NEFA_16_0	2.8 (1.41 - 5.55)	0,036*	1.73 (0.89 - 3.35)	0,305	1.49 (0.79 - 2.79)	0,288
NEFA_16_1	3.42 (1.73 - 6.79)	0,013*	2.26 (1.17 - 4.39)	0,127	2.26 (1.21 - 4.24)	0,059
NEFA_16_2	2.67 (1.34 - 5.32)	0,041*	1.61 (0.83 - 3.13)	0,410	1.66 (0.88 - 3.12)	0,187
NEFA_17_0	2.25 (1.15 - 4.4)	0,083	1.66 (0.87 - 3.18)	0,332	1.18 (0.64 - 2.19)	0,635
NEFA_17_1	2.71 (1.38 - 5.33)	0,036*	1.95 (1.02 - 3.74)	0,203	1.29 (0.69 - 2.4)	0,478
NEFA_17_2	1.82 (0.92 - 3.59)	0,179	1.09 (0.56 - 2.1)	0,873	1.37 (0.74 - 2.57)	0,380
NEFA_18_0	2.48 (1.24 - 4.94)	0,053	1.26 (0.65 - 2.46)	0,701	0.96 (0.51 - 1.82)	0,919
NEFA_18_1	2.95 (1.5 - 5.83)	0,030*	1.57 (0.82 - 3.04)	0,418	1.9 (1.02 - 3.56)	0,109
NEFA_18_2	2.4 (1.22 - 4.71)	0,059	1.17 (0.61 - 2.25)	0,793	1.35 (0.73 - 2.51)	0,401
NEFA_18_3	1.75 (0.89 - 3.45)	0,204	1.11 (0.58 - 2.13)	0,856	1.29 (0.7 - 2.4)	0,474
NEFA_19_1	2.28 (1.16 - 4.46)	0,078	1.46 (0.77 - 2.8)	0,470	1.23 (0.66 - 2.29)	0,547
NEFA_20_1	2.93 (1.48 - 5.78)	0,030*	1.37 (0.71 - 2.66)	0,562	2.19 (1.17 - 4.09)	0,062
NEFA_20_2	2.7 (1.37 - 5.33)	0,038*	2 (1.04 - 3.84)	0,203	2.28 (1.22 - 4.26)	0,056
NEFA_20_3	3.48 (1.73 - 7.03)	0,013*	2.84 (1.44 - 5.57)	0,047*	1.95 (1.02 - 3.71)	0,109
NEFA_20_4	3.74 (1.9 - 7.36)	0,007*	1.98 (1.03 - 3.81)	0,203	2.61 (1.41 - 4.85)	0,032*

NEFA_20_5	2.11 (1.06 - 4.21)	0,101	1.2 (0.62 - 2.33)	0,757	2.13 (1.14 - 3.97)	0,065
NEFA_22_3	3.1 (1.57 - 6.13)	0,025*	3.03 (1.57 - 5.85)	0,045*	2.45 (1.3 - 4.6)	0,053
NEFA_22_4	4.61 (2.31 - 9.24)	0,004*	2.87 (1.46 - 5.62)	0,046*	2.81 (1.48 - 5.34)	0,028*
NEFA_22_5	4.06 (2.06 - 8.03)	0,006*	2.21 (1.14 - 4.27)	0,137	2.31 (1.23 - 4.33)	0,055
NEFA_22_6	3.53 (1.78 - 6.99)	0,013*	1.75 (0.9 - 3.4)	0,294	1.98 (1.05 - 3.72)	0,096
NEFA_24_0	1.19 (0.6 - 2.33)	0,710	0.86 (0.45 - 1.66)	0,803	0.9 (0.49 - 1.66)	0,746
NEFA_24_1	1.44 (0.73 - 2.84)	0,416	0.76 (0.39 - 1.46)	0,643	1.63 (0.87 - 3.03)	0,200
NEFA_24_2	2.45 (1.23 - 4.89)	0,059	1.44 (0.74 - 2.79)	0,492	1.62 (0.86 - 3.06)	0,210
NEFA_24_4	2.8 (1.42 - 5.53)	0,036*	2.59 (1.35 - 4.98)	0,060	2.21 (1.19 - 4.12)	0,062
NEFA_24_5	3.92 (1.97 - 7.77)	0,007*	3.04 (1.58 - 5.88)	0,045	3.25 (1.74 - 6.07)	0,011*
NEFA_26_0	2.46 (1.26 - 4.84)	0,052	1.18 (0.62 - 2.28)	0,773	1.42 (0.76 - 2.64)	0,333
NEFA_26_1	2.75 (1.39 - 5.44)	0,036*	1.8 (0.93 - 3.5)	0,274	1.69 (0.9 - 3.17)	0,171
NEFA_26_2	2.22 (1.09 - 4.49)	0,095	1.57 (0.8 - 3.11)	0,430	1.3 (0.68 - 2.49)	0,478
lyso.PC.a.C14.0	2.28 (1.13 - 4.59)	0,091	3.37 (1.71 - 6.61)	0,045*	2.99 (1.57 - 5.66)	0,019*
lyso.PC.a.C16.0	1.47 (0.73 - 2.99)	0,404	2.7 (1.37 - 5.3)	0,060	3.24 (1.71 - 6.14)	0,011*
lyso.PC.a.C16.1	1.96 (0.95 - 4.03)	0,165	2.87 (1.44 - 5.73)	0,048*	3.77 (1.96 - 7.25)	0,008*
lyso.PC.a.C18.0	1.27 (0.62 - 2.58)	0,618	1.83 (0.93 - 3.6)	0,268	2.16 (1.14 - 4.08)	0,065
lyso.PC.a.C18.1	1.77 (0.89 - 3.52)	0,204	1.76 (0.91 - 3.43)	0,293	2.58 (1.38 - 4.83)	0,036*
lyso.PC.a.C18.2	1.17 (0.59 - 2.35)	0,738	0.87 (0.45 - 1.69)	0,807	1.15 (0.61 - 2.15)	0,699
lyso.PC.a.C18.3	1.11 (0.57 - 2.16)	0,835	1.06 (0.55 - 2.01)	0,918	1.84 (1 - 3.39)	0,112
lyso.PC.a.C20.3	2.35 (1.19 - 4.67)	0,072	2.82 (1.46 - 5.45)	0,046*	2.28 (1.22 - 4.26)	0,056
lyso.PC.a.C20.4	3.01 (1.51 - 6)	0,030*	2.58 (1.34 - 4.99)	0,062	2.58 (1.38 - 4.83)	0,036*
lyso.PC.a.C20.5	1.22 (0.61 - 2.45)	0,660	1.37 (0.71 - 2.63)	0,560	1.96 (1.05 - 3.64)	0,095
lyso.PC.a.C22.6	1.6 (0.8 - 3.19)	0,304	1.44 (0.74 - 2.78)	0,492	1.43 (0.76 - 2.67)	0,331
lyso.PC.e.C16.0	1.11 (0.55 - 2.23)	0,836	1.82 (0.93 - 3.54)	0,268	1.73 (0.92 - 3.28)	0,159
lyso.PC.e.C18.0	0.99 (0.5 - 1.96)	0,975	2.03 (1.05 - 3.91)	0,203	1.58 (0.84 - 2.96)	0,231
lyso.PC.e.C18.1	1.81 (0.89 - 3.69)	0,204	2.01 (1.02 - 3.97)	0,203	2.25 (1.18 - 4.3)	0,062
PC.aa.C30.0	2.09 (1.05 - 4.15)	0,102	1.79 (0.92 - 3.46)	0,277	1.52 (0.81 - 2.84)	0,273
PC.aa.C30.3	1.75 (0.88 - 3.48)	0,214	1.41 (0.73 - 2.71)	0,529	1.31 (0.7 - 2.48)	0,456
PC.aa.C32.0	2.93 (1.46 - 5.88)	0,034*	2.25 (1.16 - 4.37)	0,128	2.48 (1.31 - 4.68)	0,053
PC.aa.C32.1	2.65 (1.32 - 5.34)	0,045*	2.89 (1.48 - 5.66)	0,046	2.75 (1.45 - 5.21)	0,030*
PC.aa.C32.2	1.84 (0.92 - 3.67)	0,179	2.14 (1.1 - 4.14)	0,166	1.62 (0.86 - 3.04)	0,211
PC.aa.C32.3	2.16 (1.06 - 4.41)	0,102	2.12 (1.07 - 4.18)	0,198	2.39 (1.25 - 4.57)	0,055
PC.aa.C34.1	2.33 (1.17 - 4.66)	0,078	2.33 (1.21 - 4.49)	0,106	2.68 (1.43 - 5.03)	0,031*
PC.aa.C34.2	1.55 (0.77 - 3.13)	0,338	1.46 (0.75 - 2.84)	0,483	1.76 (0.93 - 3.33)	0,153
PC.aa.C34.3	1.56 (0.78 - 3.14)	0,327	1.88 (0.97 - 3.67)	0,238	2.06 (1.1 - 3.89)	0,078
PC.aa.C34.4	2.46 (1.25 - 4.83)	0,052	2.52 (1.32 - 4.82)	0,065	1.75 (0.94 - 3.25)	0,148
PC.aa.C34.5	2.21 (1.09 - 4.49)	0,095	1.55 (0.78 - 3.08)	0,430	1.75 (0.92 - 3.35)	0,159
PC.aa.C36.0	1.82 (0.9 - 3.7)	0,198	1.7 (0.86 - 3.36)	0,336	1.75 (0.92 - 3.34)	0,159
PC.aa.C36.1	2.17 (1.1 - 4.31)	0,094	1.52 (0.78 - 2.94)	0,430	2.36 (1.26 - 4.42)	0,055
PC.aa.C36.2	1.53 (0.76 - 3.09)	0,352	1.28 (0.65 - 2.51)	0,688	1.56 (0.82 - 2.96)	0,253
PC.aa.C36.3	1.74 (0.86 - 3.53)	0,226	1.88 (0.96 - 3.68)	0,238	1.85 (0.98 - 3.5)	0,122
PC.aa.C36.4	2.51 (1.26 - 5)	0,052	2.8 (1.45 - 5.41)	0,046*	2.23 (1.19 - 4.2)	0,062
PC.aa.C36.5	1.78 (0.89 - 3.54)	0,204	1.78 (0.92 - 3.47)	0,277	2.53 (1.35 - 4.74)	0,043*
PC.aa.C36.6	1.45 (0.72 - 2.92)	0,418	1.83 (0.93 - 3.58)	0,268	1.96 (1.04 - 3.71)	0,102

PC.aa.C38.0	1.36 (0.66 - 2.77)	0,509	1.16 (0.58 - 2.32)	0,804	1.92 (1 - 3.67)	0,112
PC.aa.C38.2	1.43 (0.71 - 2.9)	0,444	1.95 (0.99 - 3.83)	0,225	1.91 (1 - 3.62)	0,112
PC.aa.C38.3	2.17 (1.07 - 4.41)	0,101	2.5 (1.27 - 4.94)	0,086	2.16 (1.13 - 4.12)	0,066
PC.aa.C38.4	2.79 (1.4 - 5.56)	0,036*	2.56 (1.32 - 4.96)	0,065	2.33 (1.24 - 4.4)	0,055
PC.aa.C38.5	2.2 (1.11 - 4.35)	0,092	2.33 (1.21 - 4.48)	0,105	2.73 (1.46 - 5.1)	0,028*
PC.aa.C38.6	1.65 (0.8 - 3.38)	0,291	1.7 (0.85 - 3.39)	0,343	2.01 (1.04 - 3.88)	0,102
PC.aa.C40.0	1.88 (0.92 - 3.85)	0,179	1.05 (0.53 - 2.1)	0,930	1.57 (0.82 - 3.02)	0,253
PC.aa.C40.1	2.57 (1.28 - 5.16)	0,051	1.45 (0.74 - 2.85)	0,492	1.35 (0.72 - 2.56)	0,406
PC.aa.C40.2	2.59 (1.3 - 5.15)	0,045*	1.52 (0.78 - 2.96)	0,430	1.86 (0.99 - 3.49)	0,114
PC.aa.C40.3	2.84 (1.43 - 5.66)	0,036*	2.2 (1.13 - 4.28)	0,148	1.9 (1.01 - 3.57)	0,112
PC.aa.C40.4	3.4 (1.71 - 6.76)	0,013*	3.16 (1.63 - 6.11)	0,045*	2.4 (1.28 - 4.49)	0,055
PC.aa.C40.5	2.15 (1.09 - 4.22)	0,094	2.41 (1.26 - 4.61)	0,084	2.19 (1.18 - 4.06)	0,062
PC.aa.C40.6	2.14 (1.06 - 4.32)	0,102	1.76 (0.9 - 3.47)	0,300	2.17 (1.14 - 4.13)	0,065
PC.aa.C42.0	1.66 (0.84 - 3.31)	0,258	1.28 (0.66 - 2.5)	0,681	1.91 (1.02 - 3.56)	0,109
PC.aa.C42.5	1.88 (0.95 - 3.74)	0,165	1.47 (0.76 - 2.84)	0,470	1.86 (1 - 3.46)	0,113
PC.aa.C43.6	1.43 (0.71 - 2.87)	0,440	1.59 (0.81 - 3.11)	0,418	1.88 (1 - 3.55)	0,112
PC.aa.C44.12	1.76 (0.88 - 3.54)	0,214	1.55 (0.79 - 3.04)	0,430	2.25 (1.19 - 4.27)	0,062
PC.ae.C30.0	1.92 (0.97 - 3.83)	0,154	1.71 (0.88 - 3.32)	0,314	1.35 (0.72 - 2.53)	0,406
PC.ae.C32.0	1.86 (0.93 - 3.72)	0,174	1.69 (0.87 - 3.28)	0,323	1.71 (0.91 - 3.21)	0,166
PC.ae.C32.1	2 (0.99 - 4.04)	0,140	1.8 (0.92 - 3.55)	0,277	1.81 (0.95 - 3.47)	0,141
PC.ae.C32.2	1.63 (0.82 - 3.26)	0,282	1.19 (0.61 - 2.31)	0,773	1.44 (0.76 - 2.73)	0,327
PC.ae.C34.0	1.98 (0.99 - 3.94)	0,139	1.72 (0.89 - 3.33)	0,308	1.27 (0.67 - 2.38)	0,511
PC.ae.C34.1	1.67 (0.83 - 3.34)	0,259	1.52 (0.78 - 2.95)	0,430	1.77 (0.94 - 3.34)	0,148
PC.ae.C34.2	1.39 (0.68 - 2.84)	0,479	1.15 (0.58 - 2.28)	0,814	1.61 (0.84 - 3.11)	0,231
PC.ae.C34.3	1.41 (0.71 - 2.79)	0,450	1.11 (0.57 - 2.15)	0,852	1.42 (0.75 - 2.67)	0,340
PC.ae.C34.4	1.48 (0.74 - 2.99)	0,390	1.2 (0.61 - 2.36)	0,757	1.7 (0.89 - 3.23)	0,180
PC.ae.C36.0	1.94 (0.97 - 3.89)	0,154	1.97 (1.02 - 3.83)	0,203	2.14 (1.13 - 4.03)	0,066
PC.ae.C36.1	2.23 (1.12 - 4.44)	0,091	1.97 (1.02 - 3.84)	0,203	2.42 (1.29 - 4.54)	0,053
PC.ae.C36.2	1.12 (0.55 - 2.28)	0,835	1.02 (0.51 - 2.03)	0,971	1.15 (0.6 - 2.21)	0,699
PC.ae.C36.3	1.63 (0.81 - 3.28)	0,284	1.52 (0.78 - 2.98)	0,430	2.19 (1.16 - 4.15)	0,064
PC.ae.C36.4	2.74 (1.39 - 5.4)	0,036*	2.59 (1.35 - 4.97)	0,060	2.34 (1.26 - 4.35)	0,055
PC.ae.C36.5	2.45 (1.24 - 4.81)	0,053	2.02 (1.05 - 3.87)	0,203	2.12 (1.14 - 3.93)	0,064
PC.ae.C38.0	1.25 (0.61 - 2.56)	0,638	1.48 (0.75 - 2.95)	0,470	1.8 (0.94 - 3.45)	0,148
PC.ae.C38.2	1.37 (0.67 - 2.81)	0,498	1.29 (0.65 - 2.56)	0,681	1.23 (0.64 - 2.36)	0,583
PC.ae.C38.3	2.02 (1 - 4.1)	0,136	2.46 (1.25 - 4.84)	0,088	2.09 (1.1 - 3.97)	0,078
PC.ae.C38.4	2.02 (1.02 - 3.98)	0,120	1.9 (0.99 - 3.65)	0,225	1.88 (1.01 - 3.51)	0,112
PC.ae.C38.5	2.13 (1.07 - 4.22)	0,097	1.96 (1.02 - 3.78)	0,203	2.32 (1.24 - 4.32)	0,055
PC.ae.C38.6	2.2 (1.09 - 4.41)	0,095	1.71 (0.87 - 3.37)	0,322	2.41 (1.28 - 4.55)	0,055
PC.ae.C40.0	1.95 (0.94 - 4.04)	0,170	1.87 (0.93 - 3.77)	0,268	1.55 (0.79 - 3.04)	0,278
PC.ae.C40.1	1.76 (0.85 - 3.63)	0,226	1.32 (0.65 - 2.66)	0,672	1.53 (0.79 - 2.97)	0,279
PC.ae.C40.2	1.59 (0.79 - 3.22)	0,311	1.14 (0.58 - 2.26)	0,821	1.88 (0.99 - 3.57)	0,117
PC.ae.C40.3	2.19 (1.08 - 4.44)	0,097	1.53 (0.77 - 3.04)	0,430	1.47 (0.77 - 2.81)	0,320
PC.ae.C40.4	1.92 (0.96 - 3.84)	0,156	1.43 (0.73 - 2.79)	0,503	1.67 (0.88 - 3.16)	0,187
PC.ae.C40.5	1.74 (0.87 - 3.49)	0,217	1.32 (0.67 - 2.58)	0,650	2.21 (1.17 - 4.19)	0,062
PC.ae.C40.6	1.39 (0.68 - 2.81)	0,479	1.24 (0.63 - 2.46)	0,725	1.95 (1.02 - 3.72)	0,109

PC.ae.C42.1	2.69 (1.32 - 5.48)	0,045*	3.14 (1.58 - 6.22)	0,045*	1.52 (0.79 - 2.92)	0,279
PC.ae.C42.3	1.04 (0.51 - 2.15)	0,932	0.67 (0.34 - 1.34)	0,470	1.24 (0.64 - 2.42)	0,561
PC.ae.C42.4	1.57 (0.78 - 3.16)	0,321	1.41 (0.72 - 2.76)	0,529	1.82 (0.96 - 3.45)	0,132
PC.ae.C42.5	1.56 (0.78 - 3.14)	0,327	1.18 (0.6 - 2.3)	0,793	1.9 (1 - 3.61)	0,112
PC.ae.C42.6	1.18 (0.58 - 2.39)	0,735	0.94 (0.48 - 1.87)	0,918	1.56 (0.82 - 2.97)	0,253
SM.a.C30.1	1.85 (0.92 - 3.71)	0,179	1.75 (0.9 - 3.41)	0,294	1.55 (0.83 - 2.9)	0,253
SM.a.C32.1	1.76 (0.87 - 3.57)	0,214	2.25 (1.16 - 4.38)	0,128	1.63 (0.86 - 3.08)	0,211
SM.a.C32.2	2.22 (1.07 - 4.6)	0,101	2.36 (1.19 - 4.67)	0,120	2.5 (1.3 - 4.79)	0,053
SM.a.C33.1	1.41 (0.69 - 2.87)	0,469	1.5 (0.76 - 2.95)	0,455	1.38 (0.72 - 2.63)	0,390
SM.a.C34.1	2.02 (0.99 - 4.12)	0,139	1.59 (0.81 - 3.13)	0,421	1.58 (0.82 - 3.04)	0,250
SM.a.C34.2	2.75 (1.3 - 5.83)	0,051	1.98 (0.98 - 4.03)	0,236	2.08 (1.06 - 4.08)	0,095
SM.a.C35.0	1.66 (0.82 - 3.36)	0,264	1.32 (0.67 - 2.6)	0,650	1.84 (0.96 - 3.51)	0,134
SM.a.C35.1	1.17 (0.57 - 2.41)	0,762	1.45 (0.73 - 2.89)	0,492	1.46 (0.76 - 2.81)	0,327
SM.a.C36.1	1.95 (0.94 - 4.04)	0,165	2 (1 - 3.98)	0,212	2.53 (1.31 - 4.86)	0,053
SM.a.C36.2	1.83 (0.86 - 3.9)	0,214	2 (0.98 - 4.08)	0,229	2.33 (1.18 - 4.58)	0,062
SM.a.C36.3	1.73 (0.82 - 3.66)	0,261	1.58 (0.78 - 3.2)	0,430	2.07 (1.06 - 4.05)	0,095
SM.a.C37.1	1.3 (0.63 - 2.65)	0,585	1.53 (0.78 - 3)	0,430	2.37 (1.26 - 4.47)	0,055
SM.a.C38.2	1.34 (0.66 - 2.75)	0,525	1.59 (0.81 - 3.11)	0,418	2.17 (1.14 - 4.12)	0,065
SM.a.C38.3	1.34 (0.66 - 2.69)	0,525	1.59 (0.82 - 3.09)	0,418	1.85 (0.98 - 3.49)	0,119
SM.a.C39.1	1.95 (0.94 - 4.05)	0,170	1.77 (0.88 - 3.54)	0,305	1.94 (1 - 3.74)	0,112
SM.a.C39.2	1.11 (0.53 - 2.3)	0,843	1.1 (0.55 - 2.19)	0,865	1.67 (0.86 - 3.22)	0,202
SM.a.C40.2	2.01 (0.99 - 4.06)	0,139	2.12 (1.08 - 4.14)	0,189	2.29 (1.21 - 4.33)	0,059
SM.a.C40.5	2 (1.01 - 3.99)	0,132	1.88 (0.97 - 3.64)	0,238	3.34 (1.79 - 6.22)	0,011*
SM.a.C41.1	2.02 (1.01 - 4.05)	0,132	1.96 (1.01 - 3.77)	0,203	2.24 (1.2 - 4.18)	0,059
SM.a.C41.2	1.55 (0.76 - 3.13)	0,341	1.47 (0.75 - 2.87)	0,470	2.1 (1.11 - 3.96)	0,074
SM.a.C42.1	2.09 (1.05 - 4.14)	0,104	1.29 (0.67 - 2.5)	0,674	1.76 (0.94 - 3.28)	0,148
SM.a.C42.2	2.23 (1.09 - 4.57)	0,095	1.28 (0.65 - 2.54)	0,688	2.22 (1.16 - 4.26)	0,064
SM.a.C42.3	2.11 (1.03 - 4.31)	0,115	1.57 (0.79 - 3.1)	0,430	2.16 (1.13 - 4.13)	0,069
SM.a.C42.4	2.69 (1.35 - 5.34)	0,041	2.8 (1.45 - 5.4)	0,046*	2.76 (1.47 - 5.18)	0,028*
SM.a.C42.6	1.99 (0.97 - 4.06)	0,149	2.02 (1.02 - 4.01)	0,203	2.26 (1.18 - 4.34)	0,062
SM.a.C43.1	2.31 (1.14 - 4.7)	0,090	1.17 (0.59 - 2.32)	0,794	1.55 (0.82 - 2.96)	0,257
SM.a.C43.2	2.22 (1.1 - 4.48)	0,094	1.88 (0.96 - 3.68)	0,240	1.51 (0.8 - 2.85)	0,279
SM.a.C44.6	1.87 (0.92 - 3.78)	0,179	1.41 (0.72 - 2.78)	0,529	2.35 (1.24 - 4.46)	0,055
SM.e.C36.2	1.63 (0.78 - 3.42)	0,308	1.27 (0.63 - 2.55)	0,715	1.55 (0.79 - 3.03)	0,279
SM.e.C38.3	1.23 (0.61 - 2.45)	0,659	1.3 (0.67 - 2.52)	0,672	2.07 (1.1 - 3.9)	0,075
SM.e.C40.5	1.7 (0.86 - 3.35)	0,226	1.56 (0.81 - 2.99)	0,426	2.13 (1.15 - 3.95)	0,064
Carn	1.47 (0.73 - 2.98)	0,403	1.24 (0.64 - 2.42)	0,725	2.19 (1.17 - 4.12)	0,062
Carn.a.C10.0	2.23 (1.13 - 4.43)	0,091	1.24 (0.64 - 2.42)	0,725	1.67 (0.89 - 3.13)	0,182
Carn.a.C10.1	1.59 (0.79 - 3.19)	0,308	1.01 (0.52 - 1.98)	0,990	1.86 (0.99 - 3.5)	0,117
Carn.a.C12.0	1.7 (0.86 - 3.37)	0,226	1.17 (0.6 - 2.26)	0,794	1.93 (1.03 - 3.61)	0,106
Carn.a.C14.1	1.38 (0.7 - 2.73)	0,479	0.83 (0.43 - 1.61)	0,757	1.88 (1.01 - 3.51)	0,112
Carn.a.C14.2	1.08 (0.55 - 2.13)	0,877	0.91 (0.47 - 1.74)	0,857	1.78 (0.96 - 3.32)	0,134
Carn.a.C15.0	1.14 (0.58 - 2.26)	0,788	1 (0.52 - 1.93)	0,998	1.44 (0.77 - 2.69)	0,327
Carn.a.C16.0	1.29 (0.66 - 2.55)	0,565	1.46 (0.76 - 2.81)	0,470	1.37 (0.74 - 2.56)	0,380
Carn.a.C16.0.Oxo	1.12 (0.56 - 2.25)	0,831	0.98 (0.5 - 1.92)	0,971	1.51 (0.79 - 2.86)	0,279

Carn.a.C16.1	1.01 (0.51 - 2.01)	0,975	1 (0.52 - 1.93)	0,998	1.87 (1 - 3.5)	0,113
Carn.a.C16.2	0.74 (0.38 - 1.46)	0,499	0.82 (0.43 - 1.58)	0,742	1.56 (0.84 - 2.9)	0,234
Carn.a.C18.0	0.87 (0.44 - 1.72)	0,776	0.65 (0.34 - 1.26)	0,430	1.03 (0.55 - 1.92)	0,928
Carn.a.C18.1	1.37 (0.69 - 2.72)	0,487	1.06 (0.54 - 2.06)	0,918	2.07 (1.1 - 3.88)	0,075
Carn.a.C18.2	1.25 (0.64 - 2.47)	0,618	1.08 (0.56 - 2.07)	0,882	1.7 (0.92 - 3.16)	0,161
Carn.a.C18.2.OH	1.05 (0.53 - 2.06)	0,924	0.9 (0.47 - 1.72)	0,852	1.17 (0.63 - 2.17)	0,660
Carn.a.C2.0	2.31 (1.16 - 4.58)	0,078	1.55 (0.8 - 3)	0,430	3.11 (1.66 - 5.81)	0,012*
Carn.a.C20.0	1.52 (0.76 - 3.02)	0,349	1.81 (0.94 - 3.48)	0,266	1.29 (0.69 - 2.4)	0,480
Carn.a.C20.1	1.27 (0.65 - 2.48)	0,600	0.97 (0.51 - 1.85)	0,961	1.57 (0.85 - 2.91)	0,231
Carn.a.C20.3	1.1 (0.57 - 2.16)	0,836	1.12 (0.59 - 2.12)	0,845	1.2 (0.65 - 2.21)	0,603
Carn.a.C20.4	1.37 (0.7 - 2.69)	0,479	1.16 (0.61 - 2.23)	0,794	1.43 (0.77 - 2.67)	0,327
Carn.a.C3.0	1.76 (0.89 - 3.47)	0,204	1.28 (0.67 - 2.46)	0,674	1.75 (0.94 - 3.24)	0,148
Carn.a.C3.0.DC	1.03 (0.52 - 2.05)	0,950	0.89 (0.46 - 1.74)	0,845	1.28 (0.68 - 2.42)	0,488
Carn.a.C4.0	1.82 (0.93 - 3.57)	0,179	1.35 (0.7 - 2.59)	0,588	1.72 (0.93 - 3.19)	0,155
Carn.a.C5.0	1.85 (0.94 - 3.65)	0,170	1.58 (0.82 - 3.05)	0,418	1.77 (0.95 - 3.31)	0,141
Carn.a.C6.0	2.21 (1.11 - 4.39)	0,091	1.24 (0.63 - 2.41)	0,725	1.51 (0.8 - 2.84)	0,279
Carn.a.C6.0.OH	1.38 (0.69 - 2.77)	0,479	1.15 (0.59 - 2.25)	0,804	1.74 (0.92 - 3.29)	0,157
Carn.a.C8.0	1.78 (0.9 - 3.56)	0,204	1.2 (0.62 - 2.34)	0,757	1.31 (0.7 - 2.47)	0,456
Carn.a.C8.1	1.6 (0.79 - 3.24)	0,308	1.6 (0.81 - 3.15)	0,418	1.91 (1 - 3.62)	0,112
Carn.a.C9.0	2.15 (1.09 - 4.27)	0,095	1.09 (0.56 - 2.11)	0,870	1.4 (0.75 - 2.63)	0,353
Asn/Asp	0.35 (0.18 - 0.68)	0,030	0.54 (0.28 - 1.04)	0,238	0.51 (0.27 - 0.94)	0,093
Gln/Glu	0.57 (0.29 - 1.14)	0,214	0.49 (0.25 - 0.96)	0,203	0.31 (0.16 - 0.58)	0,011*
NEFA18.1/NEFA18.0	0.97 (0.48 - 1.94)	0,947	0.8 (0.41 - 1.58)	0,725	1.47 (0.77 - 2.81)	0,313
NEFA16.1/NEFA16.0	1.11 (0.54 - 2.27)	0,843	1.3 (0.65 - 2.6)	0,674	1.27 (0.66 - 2.46)	0,517
PC.aa/PC.ae	1.32 (0.66 - 2.65)	0,534	1.56 (0.8 - 3.05)	0,430	1.49 (0.79 - 2.82)	0,294
Lyso.PC.a/PC.aa	0.67 (0.33 - 1.36)	0,390	1.12 (0.58 - 2.19)	0,845	1.21 (0.64 - 2.29)	0,603
(lyso.PC.a.C16.0 + lyso.PC.a.C18.0)/ PC.aa	0.62 (0.3 - 1.26)	0,306	1.39 (0.71 - 2.73)	0,555	1.44 (0.75 - 2.76)	0,333
(lyso.PC.a.C18.1 + lyso.PC.a.C18.2)	0.71 (0.35 - 1.42)	0,450	0.61 (0.31 - 1.17)	0,348	0.72 (0.38 - 1.35)	0,359
Carn.a.C.16.0/ free Carn	1.06 (0.53 - 2.1)	0,912	1.31 (0.68 - 2.53)	0,650	0.87 (0.46 - 1.63)	0,683
Carn.a.C2.0/ Carn.a.C16.0	1.47 (0.74 - 2.89)	0,390	0.96 (0.5 - 1.86)	0,950	1.51 (0.81 - 2.82)	0,278

Values represent absolute differences in blood pressure (95% confidence interval) and corresponding p-values from linear regression models that reflect the difference in blood pressure (mmHg) per SDS increase in maternal early-pregnancy metabolite concentrations (μmol/L) or metabolite ratio. Model includes gestational age at time of measurement, age, parity, pre-pregnancy body mass index, educational level, smoking, folic acid supplementation and family history of hypertension. AA amino acids, NEFA non-esterified fatty acids, PC.aa diacyl-phosphatidylcholines, PC.ae acyl-alkyl-phosphatidylcholines, lyso.PC.a acyl-lysophosphatidylcholines, lyso.PC.e alkyl-lysophosphatidylcholines, Carn.a acyl-carnitines, SM sphingomyelins

[a] p-value corrected for multiple hypothesis testing using Benjamin-Hochberg FDR correction.

*****Statistically significant

Table S8. Associations of early-pregnancy individual metabolites with systolic blood pressure in early-, mid- and late pregnancy. Full model, sensitivity analysis excluding women without preexisting hypertension.

	Differences in systolic blood pressure in mmHg (95% confidence interval)					
**Metabolite**	**Early pregnancy** N = 790	**P-value**[a]	**Mid pregnancy** N = 780	**P-value**[a]	**Late pregnancy** N = 786	**P-value**[a]
Ala	3.12 (1.24 - 7.8)	0,081	2.02 (0.82 - 4.97)	0,372	2.49 (1.13 - 5.5)	0,135
Arg	5.08 (2.04 - 12.69)	0,007*	2.52 (1.02 - 6.25)	0,208	3.28 (1.49 - 7.23)	0,063
Asn	3.31 (1.32 - 8.3)	0,066	1.11 (0.45 - 2.73)	0,917	3.08 (1.4 - 6.77)	0,063
Asp	1.99 (0.79 - 5.04)	0,336	2.76 (1.12 - 6.8)	0,181	3.09 (1.4 - 6.84)	0,063
Cit	1.34 (0.5 - 3.54)	0,750	0.86 (0.33 - 2.22)	0,887	1.15 (0.5 - 2.67)	0,884
Gln	2.29 (0.92 - 5.68)	0,223	1.04 (0.43 - 2.52)	0,968	0.97 (0.44 - 2.13)	0,985
Glu	1.79 (0.71 - 4.52)	0,417	3.47 (1.41 - 8.51)	0,081	3.12 (1.41 - 6.9)	0,063
Gly	0.86 (0.35 - 2.14)	0,874	1 (0.41 - 2.42)	1,000	1.35 (0.62 - 2.97)	0,707
His	3.72 (1.46 - 9.5)	0,046*	1.59 (0.64 - 3.96)	0,560	3.12 (1.41 - 6.93)	0,063
Ile	3.22 (1.27 - 8.16)	0,077	1.32 (0.53 - 3.27)	0,755	1.61 (0.72 - 3.57)	0,498
Leu	2.88 (1.15 - 7.21)	0,110	1.32 (0.54 - 3.23)	0,751	2.12 (0.97 - 4.65)	0,222
Lys	0.81 (0.32 - 2.04)	0,810	1.39 (0.56 - 3.44)	0,700	2.99 (1.36 - 6.6)	0,068
Met	2.96 (1.19 - 7.41)	0,101	1.49 (0.6 - 3.66)	0,614	1.82 (0.83 - 4.02)	0,351
Orn	1.28 (0.5 - 3.25)	0,781	1.29 (0.53 - 3.17)	0,755	2.08 (0.94 - 4.59)	0,256
Phe	5.83 (2.38 - 14.27)	0,003*	2.51 (1.04 - 6.07)	0,208	2.4 (1.11 - 5.22)	0,135
Pro	2.39 (0.96 - 5.96)	0,193	1.88 (0.77 - 4.58)	0,390	1.56 (0.71 - 3.42)	0,498
Trp	7.77 (3.13 - 19.29)	0,001*	2.93 (1.2 - 7.17)	0,158	3.06 (1.4 - 6.68)	0,063
Ser	2.22 (0.89 - 5.52)	0,243	1.2 (0.5 - 2.9)	0,834	2.58 (1.18 - 5.64)	0,123
Thr	1.6 (0.62 - 4.12)	0,523	1.57 (0.62 - 3.95)	0,579	2.18 (0.98 - 4.87)	0,214
Tyr	5.55 (2.24 - 13.7)	0,004*	2.12 (0.88 - 5.15)	0,303	2.75 (1.26 - 5.98)	0,091
Val	5.6 (2.25 - 13.96)	0,004*	1.95 (0.8 - 4.74)	0,384	2.72 (1.24 - 5.96)	0,094
Cys	0.97 (0.38 - 2.51)	0,983	1.12 (0.44 - 2.81)	0,917	1.47 (0.65 - 3.33)	0,620
NEFA_14_0	1.59 (0.63 - 3.98)	0,520	1.46 (0.6 - 3.56)	0,628	0.85 (0.39 - 1.87)	0,873
NEFA_14_1	1.03 (0.41 - 2.61)	0,983	1.28 (0.52 - 3.14)	0,755	0.99 (0.45 - 2.21)	0,993
NEFA_15_0	1.68 (0.67 - 4.19)	0,463	1.58 (0.65 - 3.82)	0,555	0.68 (0.31 - 1.49)	0,596
NEFA_16_0	1.81 (0.72 - 4.57)	0,408	1.79 (0.73 - 4.38)	0,421	0.87 (0.39 - 1.94)	0,884
NEFA_16_1	0.93 (0.37 - 2.34)	0,928	1.4 (0.57 - 3.43)	0,693	1.01 (0.45 - 2.25)	0,993
NEFA_16_2	1.31 (0.52 - 3.33)	0,750	1.45 (0.59 - 3.57)	0,640	0.88 (0.4 - 1.97)	0,897
NEFA_17_0	1.48 (0.6 - 3.67)	0,589	1.39 (0.58 - 3.35)	0,693	0.92 (0.42 - 2.01)	0,945
NEFA_17_1	1.57 (0.63 - 3.92)	0,523	1.44 (0.6 - 3.49)	0,638	0.86 (0.39 - 1.9)	0,873
NEFA_17_2	1.73 (0.69 - 4.32)	0,439	1.1 (0.45 - 2.69)	0,923	0.82 (0.37 - 1.8)	0,870
NEFA_18_0	1.55 (0.61 - 3.93)	0,542	1.25 (0.51 - 3.07)	0,801	0.82 (0.37 - 1.81)	0,870
NEFA_18_1	1.36 (0.54 - 3.43)	0,703	1.37 (0.56 - 3.34)	0,707	1.07 (0.49 - 2.38)	0,955
NEFA_18_2	1.57 (0.63 - 3.91)	0,523	1.12 (0.46 - 2.72)	0,910	0.71 (0.32 - 1.56)	0,656
NEFA_18_3	1.9 (0.76 - 4.73)	0,356	1.37 (0.57 - 3.32)	0,705	0.64 (0.29 - 1.41)	0,498
NEFA_19_1	1.24 (0.5 - 3.09)	0,804	1.07 (0.45 - 2.58)	0,960	0.82 (0.38 - 1.8)	0,870
NEFA_20_1	1.6 (0.63 - 4.02)	0,520	1.15 (0.47 - 2.81)	0,889	1.16 (0.52 - 2.57)	0,873
NEFA_20_2	1.26 (0.5 - 3.17)	0,784	1.3 (0.53 - 3.17)	0,755	1.18 (0.53 - 2.61)	0,873
NEFA_20_3	2.69 (1.04 - 6.98)	0,166	3.08 (1.23 - 7.7)	0,149	1.77 (0.78 - 4.01)	0,399
NEFA_20_4	2.19 (0.87 - 5.47)	0,257	1.99 (0.82 - 4.85)	0,373	1.71 (0.78 - 3.76)	0,413

NEFA_20_5	2.35 (0.93 - 5.94)	0,213	1.49 (0.61 - 3.67)	0,610	1.65 (0.75 - 3.64)	0,458
NEFA_22_3	1.48 (0.59 - 3.73)	0,597	2.6 (1.06 - 6.35)	0,207	2.44 (1.1 - 5.43)	0,142
NEFA_22_4	2.5 (0.97 - 6.43)	0,191	2.85 (1.15 - 7.09)	0,174	2 (0.88 - 4.5)	0,303
NEFA_22_5	2.2 (0.87 - 5.55)	0,257	1.81 (0.74 - 4.44)	0,416	1.58 (0.71 - 3.52)	0,498
NEFA_22_6	1.77 (0.7 - 4.48)	0,422	1.29 (0.52 - 3.19)	0,755	1.27 (0.57 - 2.83)	0,821
NEFA_24_0	2.36 (0.96 - 5.82)	0,193	2.08 (0.86 - 5.03)	0,325	0.9 (0.41 - 1.95)	0,909
NEFA_24_1	1.1 (0.44 - 2.73)	0,914	0.75 (0.31 - 1.83)	0,745	0.99 (0.45 - 2.18)	0,993
NEFA_24_2	1.01 (0.39 - 2.58)	0,990	1.46 (0.59 - 3.59)	0,638	1.43 (0.64 - 3.19)	0,640
NEFA_24_4	3.18 (1.27 - 7.96)	0,077	2.83 (1.17 - 6.86)	0,169	2.58 (1.17 - 5.66)	0,126
NEFA_24_5	3.49 (1.38 - 8.8)	0,052	2.48 (1.01 - 6.07)	0,208	3.72 (1.69 - 8.21)	0,030*
NEFA_26_0	3.71 (1.5 - 9.18)	0,041	1.81 (0.75 - 4.37)	0,416	1.64 (0.75 - 3.57)	0,458
NEFA_26_1	2.02 (0.8 - 5.09)	0,320	2.53 (1.03 - 6.2)	0,208	1.88 (0.85 - 4.16)	0,325
NEFA_26_2	1.43 (0.55 - 3.71)	0,651	1.76 (0.71 - 4.38)	0,436	1.4 (0.63 - 3.14)	0,660
lyso.PC.a.C14.0	14.36 (5.71 - 36.13)	0,000*	8.73 (3.51 - 21.7)	0,001*	5.74 (2.57 - 12.83)	0,005*
lyso.PC.a.C16.0	3.67 (1.43 - 9.45)	0,049*	4.91 (1.97 - 12.28)	0,028*	2.64 (1.17 - 5.96)	0,127
lyso.PC.a.C16.1	5.19 (1.97 - 13.68)	0,011*	6.23 (2.46 - 15.82)	0,009*	4.9 (2.15 - 11.16)	0,011*
lyso.PC.a.C18.0	2.01 (0.78 - 5.18)	0,342	3.84 (1.54 - 9.54)	0,072	1.8 (0.81 - 4.04)	0,373
lyso.PC.a.C18.1	5.45 (2.18 - 13.63)	0,005*	3 (1.22 - 7.37)	0,149	1.93 (0.87 - 4.28)	0,306
lyso.PC.a.C18.2	5.44 (2.17 - 13.66)	0,005*	2.17 (0.89 - 5.28)	0,293	1.37 (0.62 - 3.02)	0,690
lyso.PC.a.C18.3	5.96 (2.45 - 14.48)	0,003*	2.16 (0.9 - 5.18)	0,293	2.86 (1.33 - 6.19)	0,073
lyso.PC.a.C20.3	8.33 (3.34 - 20.78)	0,000*	6.94 (2.84 - 16.96)	0,002*	4.94 (2.25 - 10.88)	0,008
lyso.PC.a.C20.4	5.81 (2.31 - 14.62)	0,004*	4.87 (2 - 11.88)	0,027*	2.48 (1.12 - 5.47)	0,135
lyso.PC.a.C20.5	2.61 (1.03 - 6.6)	0,167	1.3 (0.53 - 3.14)	0,755	2.3 (1.05 - 5.02)	0,159
lyso.PC.a.C22.6	2.83 (1.12 - 7.14)	0,118	1.64 (0.67 - 4.01)	0,514	1.24 (0.56 - 2.73)	0,857
lyso.PC.e.C16.0	2.84 (1.12 - 7.21)	0,118	2.95 (1.19 - 7.28)	0,159	1.99 (0.89 - 4.44)	0,303
lyso.PC.e.C18.0	0.88 (0.35 - 2.22)	0,890	1.74 (0.71 - 4.24)	0,436	1.27 (0.58 - 2.8)	0,821
lyso.PC.e.C18.1	2.54 (0.98 - 6.63)	0,191	1.3 (0.51 - 3.29)	0,755	1.56 (0.69 - 3.55)	0,519
PC.aa.C30.0	6.15 (2.46 - 15.34)	0,003*	3.06 (1.25 - 7.52)	0,149	2.37 (1.07 - 5.22)	0,155
PC.aa.C30.3	1.16 (0.46 - 2.91)	0,879	1.82 (0.75 - 4.42)	0,416	0.87 (0.39 - 1.92)	0,876
PC.aa.C32.0	3.84 (1.51 - 9.78)	0,041*	2.63 (1.06 - 6.5)	0,207	1.79 (0.8 - 4.01)	0,378
PC.aa.C32.1	5.44 (2.13 - 13.89)	0,006*	3.82 (1.54 - 9.47)	0,072	3.94 (1.77 - 8.75)	0,023*
PC.aa.C32.2	6.56 (2.61 - 16.49)	0,003*	2.8 (1.14 - 6.87)	0,174	2.55 (1.15 - 5.63)	0,129
PC.aa.C32.3	1.42 (0.54 - 3.71)	0,668	1.8 (0.72 - 4.54)	0,428	1.17 (0.52 - 2.63)	0,873
PC.aa.C34.1	3.14 (1.24 - 7.97)	0,083	3.29 (1.35 - 8.04)	0,104	2.85 (1.29 - 6.31)	0,084
PC.aa.C34.2	2.31 (0.9 - 5.89)	0,237	1.9 (0.78 - 4.68)	0,390	1.48 (0.67 - 3.3)	0,596
PC.aa.C34.3	3.22 (1.27 - 8.2)	0,077	2 (0.81 - 4.94)	0,377	1.89 (0.85 - 4.21)	0,325
PC.aa.C34.4	7.95 (3.23 - 19.53)	0,000*	3.8 (1.57 - 9.19)	0,072	2.72 (1.24 - 5.97)	0,094
PC.aa.C34.5	4.64 (1.81 - 11.95)	0,016*	1.94 (0.77 - 4.88)	0,390	1.88 (0.84 - 4.23)	0,343
PC.aa.C36.0	1.21 (0.47 - 3.15)	0,831	1.7 (0.67 - 4.32)	0,494	1.18 (0.52 - 2.68)	0,873
PC.aa.C36.1	4.12 (1.65 - 10.3)	0,026*	2.4 (0.98 - 5.88)	0,233	2.28 (1.03 - 5.04)	0,170
PC.aa.C36.2	2.43 (0.95 - 6.22)	0,193	1.82 (0.74 - 4.51)	0,416	1.4 (0.63 - 3.12)	0,660
PC.aa.C36.3	3.26 (1.27 - 8.37)	0,077	2.75 (1.11 - 6.83)	0,185	2.57 (1.16 - 5.71)	0,129
PC.aa.C36.4	3.51 (1.39 - 8.84)	0,052	3.71 (1.51 - 9.1)	0,074	2.47 (1.11 - 5.49)	0,135

PC.aa.C36.5	4.65 (1.85 - 11.68)	0,013*	1.84 (0.74 - 4.54)	0,416	3.07 (1.39 - 6.78)	0,063
PC.aa.C36.6	4.56 (1.79 - 11.61)	0,016*	2.28 (0.91 - 5.69)	0,283	2.94 (1.32 - 6.58)	0,080
PC.aa.C38.0	1.2 (0.46 - 3.14)	0,839	1.13 (0.44 - 2.88)	0,910	1.01 (0.44 - 2.28)	0,993
PC.aa.C38.2	2.57 (0.99 - 6.63)	0,182	2.65 (1.06 - 6.65)	0,207	2.34 (1.04 - 5.26)	0,170
PC.aa.C38.3	3.91 (1.51 - 10.11)	0,042*	4.77 (1.89 - 12.02)	0,033*	4.1 (1.82 - 9.25)	0,023*
PC.aa.C38.4	2.42 (0.96 - 6.14)	0,193	3.64 (1.47 - 8.99)	0,078	2.4 (1.07 - 5.37)	0,155
PC.aa.C38.5	2.59 (1.03 - 6.47)	0,167	2.66 (1.09 - 6.48)	0,195	3.34 (1.52 - 7.36)	0,063
PC.aa.C38.6	1.5 (0.57 - 3.93)	0,600	1.52 (0.6 - 3.89)	0,610	1.84 (0.8 - 4.21)	0,372
PC.aa.C40.0	1.79 (0.69 - 4.69)	0,426	0.98 (0.38 - 2.5)	0,974	0.93 (0.41 - 2.1)	0,955
PC.aa.C40.1	1.81 (0.71 - 4.63)	0,412	2.04 (0.82 - 5.07)	0,372	1.17 (0.52 - 2.61)	0,873
PC.aa.C40.2	4.1 (1.63 - 10.31)	0,027*	1.79 (0.73 - 4.38)	0,421	2.46 (1.12 - 5.44)	0,135
PC.aa.C40.3	2.58 (1.02 - 6.53)	0,168	1.93 (0.78 - 4.77)	0,390	2.46 (1.11 - 5.48)	0,135
PC.aa.C40.4	3.69 (1.46 - 9.32)	0,046*	4.39 (1.79 - 10.77)	0,037*	4 (1.81 - 8.83)	0,023*
PC.aa.C40.5	2.04 (0.82 - 5.05)	0,307	3.5 (1.45 - 8.43)	0,078	3.08 (1.41 - 6.74)	0,063
PC.aa.C40.6	2.04 (0.79 - 5.25)	0,329	1.97 (0.78 - 4.95)	0,390	2.34 (1.04 - 5.3)	0,170
PC.aa.C42.0	1.13 (0.45 - 2.86)	0,890	1.04 (0.42 - 2.58)	0,968	1.61 (0.73 - 3.54)	0,495
PC.aa.C42.5	1.38 (0.55 - 3.46)	0,694	0.96 (0.39 - 2.35)	0,968	1.27 (0.58 - 2.78)	0,821
PC.aa.C43.6	0.88 (0.34 - 2.23)	0,885	1.31 (0.53 - 3.25)	0,755	1.22 (0.55 - 2.71)	0,870
PC.aa.C44.12	1.3 (0.51 - 3.32)	0,765	1.75 (0.7 - 4.37)	0,436	1.58 (0.7 - 3.55)	0,498
PC.ae.C30.0	3.85 (1.53 - 9.68)	0,039	2.38 (0.97 - 5.85)	0,233	1.96 (0.89 - 4.33)	0,303
PC.ae.C32.0	2.54 (1 - 6.42)	0,178	2.53 (1.04 - 6.19)	0,208	1.91 (0.87 - 4.23)	0,307
PC.ae.C32.1	1.94 (0.75 - 4.99)	0,356	2.52 (1.02 - 6.26)	0,208	1.18 (0.52 - 2.64)	0,873
PC.ae.C32.2	1.58 (0.62 - 3.97)	0,523	1.43 (0.59 - 3.48)	0,654	0.75 (0.34 - 1.66)	0,742
PC.ae.C34.0	2.81 (1.11 - 7.1)	0,120	2.56 (1.04 - 6.27)	0,208	1.71 (0.77 - 3.78)	0,417
PC.ae.C34.1	2.22 (0.88 - 5.63)	0,257	2.1 (0.85 - 5.15)	0,325	1.82 (0.82 - 4.05)	0,354
PC.ae.C34.2	1.63 (0.63 - 4.23)	0,514	1.52 (0.61 - 3.8)	0,608	1.15 (0.51 - 2.6)	0,884
PC.ae.C34.3	1.66 (0.67 - 4.14)	0,473	1.63 (0.67 - 3.92)	0,514	0.86 (0.39 - 1.9)	0,873
PC.ae.C34.4	1.84 (0.72 - 4.71)	0,399	1.38 (0.56 - 3.42)	0,705	1.49 (0.66 - 3.32)	0,596
PC.ae.C36.0	0.97 (0.38 - 2.46)	0,983	1.79 (0.73 - 4.4)	0,421	1.09 (0.49 - 2.43)	0,945
PC.ae.C36.1	1.83 (0.72 - 4.64)	0,399	2.45 (0.99 - 6.05)	0,225	2.53 (1.14 - 5.6)	0,134
PC.ae.C36.2	1.12 (0.43 - 2.91)	0,902	1.04 (0.42 - 2.61)	0,968	1.07 (0.48 - 2.41)	0,959
PC.ae.C36.3	1.88 (0.74 - 4.76)	0,381	1.78 (0.72 - 4.4)	0,428	1.84 (0.83 - 4.09)	0,351
PC.ae.C36.4	2.52 (1.01 - 6.27)	0,175	4.2 (1.73 - 10.2)	0,040*	2.19 (1 - 4.81)	0,200
PC.ae.C36.5	2.02 (0.81 - 5)	0,317	2.22 (0.92 - 5.39)	0,283	1.42 (0.65 - 3.11)	0,640
PC.ae.C38.0	1.64 (0.63 - 4.26)	0,513	1.03 (0.41 - 2.6)	0,968	1.59 (0.7 - 3.59)	0,498
PC.ae.C38.2	1.99 (0.76 - 5.18)	0,352	2.35 (0.93 - 5.93)	0,271	1.31 (0.57 - 2.97)	0,800
PC.ae.C38.3	2.5 (0.97 - 6.42)	0,191	3.6 (1.44 - 8.97)	0,081	3.11 (1.39 - 6.98)	0,063
PC.ae.C38.4	1.94 (0.78 - 4.82)	0,346	3.08 (1.27 - 7.47)	0,137	1.93 (0.88 - 4.25)	0,306
PC.ae.C38.5	1.67 (0.67 - 4.19)	0,468	2.26 (0.93 - 5.52)	0,276	1.57 (0.71 - 3.46)	0,498
PC.ae.C38.6	1.72 (0.67 - 4.38)	0,454	1.77 (0.71 - 4.43)	0,436	1.59 (0.71 - 3.54)	0,498
PC.ae.C40.0	1.79 (0.67 - 4.73)	0,439	1.15 (0.45 - 2.97)	0,892	1.48 (0.64 - 3.43)	0,628
PC.ae.C40.1	2.97 (1.13 - 7.8)	0,118	1.33 (0.52 - 3.42)	0,755	1.74 (0.76 - 3.98)	0,422
PC.ae.C40.2	1.22 (0.48 - 3.14)	0,824	0.97 (0.39 - 2.42)	0,968	2.13 (0.96 - 4.74)	0,230
PC.ae.C40.3	2.08 (0.8 - 5.37)	0,318	2.25 (0.89 - 5.69)	0,293	1.63 (0.72 - 3.7)	0,498

PC.ae.C40.4	2.18 (0.86 - 5.53)	0,263	3.52 (1.43 - 8.7)	0,081	2.04 (0.91 - 4.57)	0,284
PC.ae.C40.5	1.14 (0.45 - 2.91)	0,885	1.55 (0.63 - 3.84)	0,579	1.61 (0.72 - 3.6)	0,498
PC.ae.C40.6	1.02 (0.39 - 2.63)	0,988	1.22 (0.49 - 3.06)	0,831	1.48 (0.65 - 3.34)	0,613
PC.ae.C42.1	2.17 (0.83 - 5.65)	0,286	2.25 (0.88 - 5.71)	0,293	1.6 (0.71 - 3.64)	0,498
PC.ae.C42.3	0.86 (0.33 - 2.26)	0,881	0.64 (0.25 - 1.62)	0,579	1.17 (0.51 - 2.66)	0,873
PC.ae.C42.4	2.18 (0.86 - 5.56)	0,263	2.17 (0.88 - 5.38)	0,303	1.94 (0.87 - 4.31)	0,306
PC.ae.C42.5	1.25 (0.49 - 3.18)	0,804	1.74 (0.7 - 4.32)	0,436	1.79 (0.8 - 4.01)	0,378
PC.ae.C42.6	1.02 (0.4 - 2.62)	0,988	0.95 (0.38 - 2.37)	0,968	1.16 (0.52 - 2.59)	0,873
SM.a.C30.1	2.27 (0.9 - 5.75)	0,241	2.53 (1.03 - 6.2)	0,208	1.93 (0.88 - 4.24)	0,306
SM.a.C32.1	1.84 (0.72 - 4.7)	0,399	2.41 (0.98 - 5.94)	0,233	2 (0.9 - 4.45)	0,297
SM.a.C32.2	1.86 (0.7 - 4.93)	0,412	3.18 (1.25 - 8.11)	0,149	3.17 (1.4 - 7.19)	0,063
SM.a.C33.1	1.02 (0.39 - 2.63)	0,988	1.54 (0.62 - 3.8)	0,587	1.27 (0.57 - 2.84)	0,821
SM.a.C34.1	1.01 (0.39 - 2.63)	0,988	1.63 (0.65 - 4.09)	0,531	1.3 (0.57 - 2.93)	0,801
SM.a.C34.2	1.12 (0.41 - 3.05)	0,910	2 (0.76 - 5.29)	0,390	1.67 (0.71 - 3.9)	0,495
SM.a.C35.0	0.77 (0.3 - 1.97)	0,765	0.88 (0.36 - 2.18)	0,908	1.25 (0.56 - 2.78)	0,846
SM.a.C35.1	0.37 (0.14 - 0.98)	0,168	0.83 (0.33 - 2.09)	0,841	0.73 (0.33 - 1.65)	0,711
SM.a.C36.1	0.74 (0.28 - 1.96)	0,738	1.46 (0.57 - 3.75)	0,648	1 (0.44 - 2.3)	0,993
SM.a.C36.2	0.43 (0.16 - 1.18)	0,263	1.19 (0.45 - 3.16)	0,871	0.88 (0.37 - 2.07)	0,897
SM.a.C36.3	0.76 (0.28 - 2.06)	0,765	0.88 (0.34 - 2.31)	0,910	0.99 (0.43 - 2.31)	0,993
SM.a.C37.1	1.22 (0.47 - 3.17)	0,824	1.29 (0.51 - 3.22)	0,755	2.18 (0.98 - 4.85)	0,214
SM.a.C38.2	1.29 (0.5 - 3.36)	0,768	1.9 (0.77 - 4.71)	0,390	1.3 (0.58 - 2.9)	0,800
SM.a.C38.3	1.24 (0.48 - 3.16)	0,810	1.49 (0.61 - 3.65)	0,610	0.96 (0.44 - 2.13)	0,985
SM.a.C39.1	2.2 (0.83 - 5.83)	0,286	2.26 (0.89 - 5.76)	0,293	2.32 (1.02 - 5.29)	0,180
SM.a.C39.2	0.73 (0.28 - 1.93)	0,723	0.76 (0.3 - 1.91)	0,755	1.21 (0.54 - 2.73)	0,873
SM.a.C40.2	1.88 (0.73 - 4.83)	0,384	2.83 (1.14 - 7)	0,174	2.02 (0.91 - 4.5)	0,290
SM.a.C40.5	3.42 (1.36 - 8.59)	0,056	2.05 (0.83 - 5.03)	0,357	4.07 (1.86 - 8.9)	0,023*
SM.a.C41.1	2.9 (1.14 - 7.36)	0,111	1.94 (0.79 - 4.72)	0,390	1.71 (0.78 - 3.75)	0,413
SM.a.C41.2	1.33 (0.52 - 3.41)	0,744	1.03 (0.42 - 2.54)	0,968	1.99 (0.9 - 4.4)	0,300
SM.a.C42.1	1.51 (0.6 - 3.82)	0,579	1.06 (0.43 - 2.6)	0,968	1.3 (0.59 - 2.86)	0,800
SM.a.C42.2	1.09 (0.42 - 2.84)	0,928	0.85 (0.34 - 2.16)	0,875	1.42 (0.63 - 3.22)	0,656
SM.a.C42.3	1 (0.38 - 2.61)	0,992	1.56 (0.62 - 3.95)	0,579	1.59 (0.7 - 3.6)	0,498
SM.a.C42.4	2.4 (0.95 - 6.03)	0,193	2.78 (1.13 - 6.81)	0,174	1.95 (0.88 - 4.33)	0,306
SM.a.C42.6	1.96 (0.75 - 5.11)	0,356	1.95 (0.77 - 4.94)	0,390	1.94 (0.85 - 4.43)	0,319
SM.a.C43.1	1.59 (0.61 - 4.13)	0,523	1.07 (0.42 - 2.69)	0,966	1.05 (0.47 - 2.37)	0,973
SM.a.C43.2	1.17 (0.46 - 2.98)	0,874	1.22 (0.49 - 3.04)	0,831	0.93 (0.42 - 2.08)	0,955
SM.a.C44.6	1.39 (0.54 - 3.6)	0,694	1.63 (0.65 - 4.12)	0,534	1.86 (0.82 - 4.18)	0,351
SM.e.C36.2	0.47 (0.18 - 1.27)	0,320	0.73 (0.28 - 1.87)	0,720	0.89 (0.39 - 2.05)	0,913
SM.e.C38.3	0.97 (0.39 - 2.45)	0,983	1.28 (0.53 - 3.13)	0,755	1.02 (0.46 - 2.24)	0,993
SM.e.C40.5	1.49 (0.6 - 3.71)	0,589	1.85 (0.76 - 4.51)	0,398	1.11 (0.51 - 2.43)	0,913
Carn	1.14 (0.44 - 2.95)	0,885	1.93 (0.78 - 4.77)	0,390	2.5 (1.13 - 5.56)	0,135
Carn.a.C10.0	0.47 (0.19 - 1.18)	0,274	0.83 (0.34 - 2.04)	0,834	1.03 (0.46 - 2.29)	0,985
Carn.a.C10.1	0.56 (0.22 - 1.43)	0,417	0.6 (0.24 - 1.5)	0,514	0.84 (0.37 - 1.87)	0,873
Carn.a.C12.0	0.68 (0.27 - 1.72)	0,604	0.82 (0.33 - 2.01)	0,831	1.17 (0.53 - 2.58)	0,873
Carn.a.C14.1	0.61 (0.24 - 1.52)	0,487	0.65 (0.26 - 1.59)	0,579	1 (0.45 - 2.21)	0,993
Carn.a.C14.2	0.52 (0.21 - 1.3)	0,356	0.56 (0.23 - 1.35)	0,416	0.97 (0.44 - 2.13)	0,985

Carn.a.C15.0	0.89 (0.36 - 2.24)	0,902	1 (0.41 - 2.42)	0,999	1.74 (0.79 - 3.83)	0,399
Carn.a.C16.0	0.74 (0.3 - 1.85)	0,720	0.84 (0.34 - 2.03)	0,838	0.89 (0.4 - 1.95)	0,897
Carn.a.C16.0.Oxo	1.11 (0.44 - 2.83)	0,910	0.96 (0.38 - 2.39)	0,968	1.03 (0.46 - 2.32)	0,985
Carn.a.C16.1	0.34 (0.14 - 0.86)	0,108	0.56 (0.23 - 1.37)	0,421	1.19 (0.53 - 2.64)	0,873
Carn.a.C16.2	0.57 (0.23 - 1.41)	0,417	0.65 (0.27 - 1.56)	0,579	1.19 (0.54 - 2.61)	0,873
Carn.a.C18.0	1.21 (0.48 - 3.01)	0,830	0.51 (0.21 - 1.23)	0,377	0.99 (0.45 - 2.19)	0,993
Carn.a.C18.1	0.53 (0.21 - 1.35)	0,381	0.73 (0.29 - 1.81)	0,708	1.43 (0.64 - 3.18)	0,640
Carn.a.C18.2	0.68 (0.27 - 1.69)	0,594	0.91 (0.37 - 2.21)	0,923	0.94 (0.43 - 2.07)	0,972
Carn.a.C18.2.OH	0.93 (0.38 - 2.31)	0,928	0.87 (0.36 - 2.1)	0,887	1.22 (0.55 - 2.68)	0,870
Carn.a.C2.0	1.69 (0.67 - 4.26)	0,465	1.82 (0.75 - 4.45)	0,416	1.85 (0.83 - 4.1)	0,347
Carn.a.C20.0	1.45 (0.57 - 3.65)	0,618	1.87 (0.77 - 4.53)	0,391	0.85 (0.38 - 1.87)	0,873
Carn.a.C20.1	1.58 (0.64 - 3.91)	0,520	1.03 (0.43 - 2.46)	0,968	1.38 (0.64 - 3.01)	0,660
Carn.a.C20.3	0.91 (0.37 - 2.24)	0,914	1.25 (0.52 - 2.99)	0,786	0.7 (0.32 - 1.53)	0,640
Carn.a.C20.4	0.77 (0.31 - 1.92)	0,760	1.03 (0.42 - 2.48)	0,968	0.95 (0.43 - 2.09)	0,973
Carn.a.C3.0	2.9 (1.16 - 7.23)	0,107	2.28 (0.94 - 5.51)	0,263	2.32 (1.06 - 5.07)	0,159
Carn.a.C3.0.DC	0.79 (0.31 - 2)	0,784	1.06 (0.43 - 2.62)	0,968	1.6 (0.72 - 3.58)	0,498
Carn.a.C4.0	3.54 (1.43 - 8.75)	0,046*	2.36 (0.97 - 5.7)	0,233	1.9 (0.87 - 4.16)	0,306
Carn.a.C5.0	2.9 (1.16 - 7.21)	0,107	2.47 (1.02 - 6.02)	0,208	2.82 (1.29 - 6.2)	0,084
Carn.a.C6.0	0.86 (0.34 - 2.18)	0,874	1.08 (0.44 - 2.67)	0,958	1.09 (0.49 - 2.42)	0,945
Carn.a.C6.0.OH	0.58 (0.23 - 1.48)	0,448	1.22 (0.49 - 3.03)	0,831	1.75 (0.78 - 3.91)	0,406
Carn.a.C8.0	0.51 (0.2 - 1.29)	0,346	0.74 (0.3 - 1.83)	0,730	0.86 (0.38 - 1.91)	0,873
Carn.a.C8.1	0.85 (0.33 - 2.19)	0,865	1.03 (0.41 - 2.6)	0,968	0.7 (0.31 - 1.59)	0,656
Carn.a.C9.0	1.63 (0.65 - 4.08)	0,504	1.29 (0.53 - 3.16)	0,755	1.68 (0.76 - 3.7)	0,436
Asn/Asp	1.08 (0.44 - 2.66)	0,928	0.48 (0.2 - 1.14)	0,303	0.86 (0.4 - 1.87)	0,873
Gln/Glu	1.08 (0.43 - 2.72)	0,928	0.38 (0.15 - 0.92)	0,198	0.38 (0.17 - 0.83)	0,113
NEFA18.1/NEFA18.0	0.82 (0.32 - 2.09)	0,824	0.65 (0.26 - 1.62)	0,593	0.92 (0.41 - 2.05)	0,945
NEFA16.1/NEFA16.0	0.44 (0.17 - 1.15)	0,257	0.66 (0.26 - 1.68)	0,610	1.21 (0.53 - 2.74)	0,873
PC.aa/PC.ae	3.62 (1.42 - 9.24)	0,049	2.21 (0.9 - 5.46)	0,293	2.34 (1.06 - 5.17)	0,159
Lyso.PC.a/PC.aa	1.51 (0.58 - 3.91)	0,589	1.89 (0.77 - 4.62)	0,390	1.05 (0.47 - 2.34)	0,973
(lyso.PC.a.C16.0 + lyso.PC.a.C18.0)/ PC.aa	0.94 (0.36 - 2.46)	0,950	1.96 (0.79 - 4.86)	0,390	1.05 (0.47 - 2.35)	0,973
(lyso.PC.a.C18.1 + lyso.PC.a.C18.2)	2.43 (0.96 - 6.18)	0,193	1.18 (0.49 - 2.87)	0,856	0.79 (0.36 - 1.74)	0,821
Carn.a.C.16.0/ free Carn	0.65 (0.26 - 1.63)	0,542	0.58 (0.24 - 1.41)	0,436	0.49 (0.22 - 1.08)	0,268
Carn.a.C2.0/ Carn.a.C16.0	1.88 (0.75 - 4.73)	0,374	1.92 (0.78 - 4.7)	0,390	1.71 (0.78 - 3.76)	0,413

Values represent absolute differences in blood pressure (95% confidence interval) and corresponding p-values from linear regression models that reflect the difference in blood pressure (mmHg) per SDS increase in maternal early-pregnancy metabolite concentrations (μmol/L) or metabolite ratio. Model includes gestational age at time of measurement, age, parity, pre-pregnancy body mass index, educational level, smoking and folic acid supplementation. AA amino acids, NEFA non-esterified fatty acids, PC.aa diacyl-phosphatidylcholines, PC.ae acyl-alkyl-phosphatidylcholines, lyso.PC.a acyl-lysophosphatidylcholines, lyso.PC.e alkyl-lysophosphatidylcholines, Carn.a acyl-carnitines, SM sphingomyelins

[a] p-value corrected for multiple hypothesis testing using Benjamin-Hochberg FDR correction.

*****Statistically significant

Table S9. Associations of early-pregnancy individual metabolites with diastolic blood pressure in early-, mid- and late pregnancy. Full model, sensitivity analysis excluding women without preexisting hypertension.

	Differences in diastolic blood pressure in mmHg (95% confidence interval)					
**Metabolite**	**Early pregnancy** N = 790	**P-value[a]**	**Mid pregnancy** N = 780	**P-value[a]**	**Late pregnancy** N = 786	**P-value[a]**
Ala	1.1 (0.55 - 2.18)	0,843	1.55 (0.8 - 3.02)	0,423	2.41 (1.29 - 4.51)	0,054
Arg	1.95 (0.98 - 3.87)	0,147	1.13 (0.58 - 2.2)	0,843	1.78 (0.95 - 3.33)	0,136
Asn	0.95 (0.47 - 1.88)	0,913	0.84 (0.43 - 1.64)	0,776	1.81 (0.96 - 3.4)	0,131
Asp	2.69 (1.34 - 5.41)	0,038*	1.77 (0.91 - 3.43)	0,284	3.15 (1.68 - 5.9)	0,009
Cit	0.98 (0.47 - 2.04)	0,977	0.73 (0.36 - 1.46)	0,599	1.14 (0.58 - 2.21)	0,724
Gln	1.31 (0.66 - 2.59)	0,545	0.68 (0.35 - 1.31)	0,465	0.78 (0.42 - 1.47)	0,508
Glu	2.67 (1.34 - 5.31)	0,038*	2.02 (1.04 - 3.92)	0,208	3.75 (2.01 - 6.99)	0,008*
Gly	0.77 (0.39 - 1.52)	0,552	0.9 (0.47 - 1.72)	0,857	1.6 (0.86 - 2.98)	0,212
His	0.99 (0.49 - 2)	0,978	0.83 (0.42 - 1.64)	0,775	1.98 (1.04 - 3.77)	0,103
Ile	0.67 (0.34 - 1.35)	0,385	0.83 (0.42 - 1.63)	0,775	1.47 (0.78 - 2.79)	0,300
Leu	0.79 (0.4 - 1.57)	0,595	0.84 (0.43 - 1.63)	0,775	1.72 (0.92 - 3.21)	0,152
Lys	0.47 (0.23 - 0.93)	0,099	1.19 (0.61 - 2.31)	0,776	2.21 (1.18 - 4.14)	0,064
Met	0.97 (0.49 - 1.93)	0,957	0.88 (0.45 - 1.71)	0,819	1.27 (0.68 - 2.38)	0,513
Orn	0.68 (0.34 - 1.36)	0,390	1 (0.51 - 1.94)	0,999	2.13 (1.13 - 4.01)	0,071
Phe	1.71 (0.87 - 3.36)	0,216	1.27 (0.66 - 2.44)	0,687	2.08 (1.12 - 3.84)	0,071
Pro	0.96 (0.49 - 1.9)	0,939	0.81 (0.42 - 1.55)	0,715	1.43 (0.77 - 2.67)	0,318
Trp	1.11 (0.56 - 2.22)	0,821	1.05 (0.54 - 2.04)	0,927	1.48 (0.79 - 2.76)	0,289
Ser	1.57 (0.79 - 3.1)	0,304	0.97 (0.5 - 1.85)	0,939	2.1 (1.13 - 3.91)	0,071
Thr	0.82 (0.41 - 1.66)	0,669	1.04 (0.52 - 2.05)	0,939	1.65 (0.87 - 3.12)	0,199
Tyr	1.29 (0.65 - 2.54)	0,566	0.95 (0.49 - 1.83)	0,927	1.62 (0.87 - 3.01)	0,202
Val	1.24 (0.63 - 2.47)	0,619	0.95 (0.49 - 1.82)	0,921	1.72 (0.92 - 3.21)	0,152
Cys	0.36 (0.18 - 0.73)	0,038*	0.71 (0.36 - 1.39)	0,525	0.99 (0.52 - 1.89)	0,977
NEFA_14_0	2.16 (1.09 - 4.27)	0,094	1.37 (0.71 - 2.65)	0,557	1.1 (0.59 - 2.05)	0,786
NEFA_14_1	3.23 (1.63 - 6.42)	0,019*	2 (1.03 - 3.88)	0,208	1.39 (0.73 - 2.62)	0,377
NEFA_15_0	2.19 (1.11 - 4.31)	0,091	1.49 (0.78 - 2.87)	0,443	0.87 (0.46 - 1.62)	0,675
NEFA_16_0	2.78 (1.4 - 5.52)	0,038	1.72 (0.88 - 3.33)	0,309	1.46 (0.78 - 2.76)	0,301
NEFA_16_1	3.3 (1.66 - 6.56)	0,018*	2.2 (1.14 - 4.27)	0,144	2.16 (1.15 - 4.06)	0,067
NEFA_16_2	2.62 (1.32 - 5.23)	0,043*	1.59 (0.81 - 3.09)	0,407	1.62 (0.86 - 3.06)	0,208
NEFA_17_0	2.25 (1.14 - 4.41)	0,085	1.66 (0.86 - 3.19)	0,340	1.17 (0.63 - 2.18)	0,646
NEFA_17_1	2.69 (1.36 - 5.31)	0,038*	1.94 (1.01 - 3.73)	0,217	1.27 (0.68 - 2.38)	0,515
NEFA_17_2	1.81 (0.91 - 3.59)	0,187	1.08 (0.56 - 2.1)	0,878	1.37 (0.73 - 2.56)	0,390
NEFA_18_0	2.49 (1.25 - 4.97)	0,054	1.27 (0.65 - 2.47)	0,704	0.96 (0.51 - 1.83)	0,917
NEFA_18_1	2.92 (1.48 - 5.79)	0,035*	1.56 (0.81 - 3.02)	0,414	1.87 (1 - 3.51)	0,111
NEFA_18_2	2.38 (1.21 - 4.69)	0,064	1.16 (0.6 - 2.24)	0,793	1.33 (0.71 - 2.48)	0,428
NEFA_18_3	1.72 (0.87 - 3.4)	0,216	1.09 (0.57 - 2.1)	0,872	1.26 (0.68 - 2.35)	0,516
NEFA_19_1	2.26 (1.15 - 4.44)	0,085	1.45 (0.76 - 2.79)	0,468	1.21 (0.65 - 2.27)	0,577
NEFA_20_1	2.93 (1.47 - 5.81)	0,035*	1.37 (0.7 - 2.66)	0,571	2.17 (1.16 - 4.09)	0,066
NEFA_20_2	2.73 (1.38 - 5.4)	0,038*	2.01 (1.04 - 3.88)	0,208	2.29 (1.22 - 4.29)	0,064
NEFA_20_3	3.45 (1.7 - 7)	0,018*	2.8 (1.43 - 5.52)	0,049*	1.91 (1 - 3.66)	0,109
NEFA_20_4	3.82 (1.94 - 7.53)	0,007*	2.02 (1.05 - 3.88)	0,208	2.68 (1.44 - 4.99)	0,032*

NEFA_20_5	2.08 (1.04 - 4.15)	0,113	1.2 (0.62 - 2.32)	0,775	2.09 (1.12 - 3.92)	0,071
NEFA_22_3	3.09 (1.55 - 6.14)	0,027*	3.01 (1.55 - 5.84)	0,047*	2.4 (1.27 - 4.55)	0,054
NEFA_22_4	4.51 (2.25 - 9.06)	0,005*	2.82 (1.43 - 5.53)	0,049*	2.73 (1.43 - 5.2)	0,033*
NEFA_22_5	3.99 (2.01 - 7.91)	0,007*	2.18 (1.12 - 4.22)	0,150	2.25 (1.19 - 4.24)	0,064
NEFA_22_6	3.56 (1.79 - 7.08)	0,012*	1.77 (0.91 - 3.44)	0,284	1.99 (1.06 - 3.75)	0,096
NEFA_24_0	1.25 (0.64 - 2.46)	0,608	0.9 (0.47 - 1.73)	0,858	0.95 (0.51 - 1.76)	0,883
NEFA_24_1	1.51 (0.76 - 2.98)	0,349	0.79 (0.41 - 1.52)	0,691	1.7 (0.91 - 3.18)	0,158
NEFA_24_2	2.46 (1.22 - 4.95)	0,061	1.44 (0.74 - 2.81)	0,493	1.62 (0.85 - 3.07)	0,212
NEFA_24_4	2.8 (1.41 - 5.54)	0,036*	2.58 (1.34 - 4.96)	0,058	2.19 (1.18 - 4.1)	0,064
NEFA_24_5	3.85 (1.93 - 7.65)	0,007*	2.99 (1.55 - 5.79)	0,047*	3.15 (1.68 - 5.92)	0,009*
NEFA_26_0	2.54 (1.29 - 5)	0,046*	1.22 (0.63 - 2.34)	0,755	1.47 (0.79 - 2.74)	0,297
NEFA_26_1	2.82 (1.42 - 5.61)	0,036*	1.84 (0.94 - 3.58)	0,254	1.75 (0.93 - 3.29)	0,151
NEFA_26_2	2.28 (1.12 - 4.65)	0,091	1.61 (0.81 - 3.19)	0,407	1.35 (0.7 - 2.6)	0,428
lyso.PC.a.C14.0	2.33 (1.15 - 4.69)	0,085	3.44 (1.75 - 6.76)	0,047*	3.07 (1.61 - 5.83)	0,015*
lyso.PC.a.C16.0	1.51 (0.74 - 3.07)	0,370	2.77 (1.41 - 5.44)	0,050	3.35 (1.77 - 6.37)	0,009*
lyso.PC.a.C16.1	1.96 (0.95 - 4.06)	0,162	2.91 (1.46 - 5.82)	0,049*	3.81 (1.97 - 7.35)	0,008*
lyso.PC.a.C18.0	1.33 (0.66 - 2.71)	0,540	1.91 (0.97 - 3.76)	0,241	2.29 (1.21 - 4.35)	0,064
lyso.PC.a.C18.1	1.82 (0.91 - 3.64)	0,187	1.82 (0.94 - 3.54)	0,259	2.69 (1.44 - 5.03)	0,032*
lyso.PC.a.C18.2	1.25 (0.62 - 2.49)	0,619	0.92 (0.48 - 1.78)	0,875	1.23 (0.65 - 2.31)	0,559
lyso.PC.a.C18.3	1.13 (0.58 - 2.21)	0,794	1.08 (0.57 - 2.05)	0,885	1.89 (1.02 - 3.48)	0,103
lyso.PC.a.C20.3	2.34 (1.17 - 4.65)	0,077	2.8 (1.44 - 5.43)	0,049*	2.26 (1.21 - 4.24)	0,064
lyso.PC.a.C20.4	3.09 (1.55 - 6.18)	0,027*	2.66 (1.37 - 5.14)	0,052	2.67 (1.43 - 5.01)	0,032*
lyso.PC.a.C20.5	1.25 (0.62 - 2.51)	0,618	1.41 (0.73 - 2.7)	0,515	2.02 (1.09 - 3.76)	0,079
lyso.PC.a.C22.6	1.62 (0.81 - 3.24)	0,284	1.46 (0.76 - 2.83)	0,468	1.46 (0.78 - 2.74)	0,301
lyso.PC.e.C16.0	1.16 (0.58 - 2.33)	0,760	1.89 (0.97 - 3.68)	0,241	1.83 (0.97 - 3.46)	0,129
lyso.PC.e.C18.0	1.01 (0.51 - 2.01)	0,978	2.08 (1.08 - 4)	0,184	1.63 (0.87 - 3.06)	0,201
lyso.PC.e.C18.1	1.8 (0.88 - 3.67)	0,210	2.01 (1.01 - 3.98)	0,217	2.25 (1.18 - 4.31)	0,064
PC.aa.C30.0	2.05 (1.03 - 4.08)	0,117	1.76 (0.91 - 3.41)	0,284	1.47 (0.78 - 2.77)	0,300
PC.aa.C30.3	1.76 (0.88 - 3.51)	0,210	1.41 (0.73 - 2.73)	0,515	1.31 (0.69 - 2.49)	0,459
PC.aa.C32.0	2.89 (1.44 - 5.8)	0,036*	2.22 (1.14 - 4.32)	0,144	2.41 (1.27 - 4.57)	0,054
PC.aa.C32.1	2.53 (1.26 - 5.11)	0,054	2.78 (1.42 - 5.44)	0,049	2.59 (1.36 - 4.92)	0,043
PC.aa.C32.2	1.87 (0.93 - 3.75)	0,169	2.16 (1.12 - 4.19)	0,152	1.64 (0.87 - 3.1)	0,200
PC.aa.C32.3	2.19 (1.07 - 4.48)	0,104	2.13 (1.08 - 4.21)	0,184	2.4 (1.25 - 4.6)	0,060
PC.aa.C34.1	2.27 (1.13 - 4.53)	0,088	2.26 (1.17 - 4.38)	0,126	2.56 (1.36 - 4.82)	0,043*
PC.aa.C34.2	1.59 (0.78 - 3.21)	0,306	1.47 (0.76 - 2.87)	0,468	1.79 (0.94 - 3.39)	0,140
PC.aa.C34.3	1.55 (0.77 - 3.12)	0,333	1.87 (0.96 - 3.64)	0,241	2.03 (1.07 - 3.84)	0,087
PC.aa.C34.4	2.44 (1.24 - 4.81)	0,054	2.49 (1.3 - 4.78)	0,068	1.72 (0.92 - 3.21)	0,152
PC.aa.C34.5	2.31 (1.13 - 4.69)	0,088	1.61 (0.81 - 3.19)	0,407	1.84 (0.96 - 3.52)	0,131
PC.aa.C36.0	1.83 (0.9 - 3.73)	0,195	1.71 (0.86 - 3.4)	0,335	1.76 (0.92 - 3.38)	0,152
PC.aa.C36.1	2.16 (1.09 - 4.29)	0,094	1.51 (0.78 - 2.93)	0,438	2.33 (1.24 - 4.37)	0,060
PC.aa.C36.2	1.58 (0.78 - 3.2)	0,306	1.31 (0.67 - 2.57)	0,656	1.61 (0.85 - 3.07)	0,215
PC.aa.C36.3	1.75 (0.86 - 3.55)	0,221	1.88 (0.96 - 3.68)	0,241	1.84 (0.97 - 3.5)	0,128
PC.aa.C36.4	2.53 (1.27 - 5.05)	0,052	2.81 (1.45 - 5.42)	0,049	2.23 (1.18 - 4.21)	0,064
PC.aa.C36.5	1.78 (0.89 - 3.56)	0,201	1.8 (0.92 - 3.5)	0,274	2.54 (1.35 - 4.78)	0,043*
PC.aa.C36.6	1.44 (0.71 - 2.9)	0,430	1.81 (0.92 - 3.56)	0,274	1.94 (1.02 - 3.69)	0,103

PC.aa.C38.0	1.4 (0.68 - 2.86)	0,468	1.19 (0.59 - 2.37)	0,776	1.97 (1.03 - 3.79)	0,103
PC.aa.C38.2	1.38 (0.68 - 2.8)	0,481	1.89 (0.96 - 3.72)	0,241	1.82 (0.96 - 3.48)	0,131
PC.aa.C38.3	2.14 (1.05 - 4.37)	0,109	2.47 (1.25 - 4.89)	0,096	2.12 (1.11 - 4.06)	0,074
PC.aa.C38.4	2.77 (1.39 - 5.54)	0,038	2.54 (1.31 - 4.94)	0,068	2.3 (1.22 - 4.35)	0,064
PC.aa.C38.5	2.18 (1.1 - 4.33)	0,093	2.32 (1.21 - 4.47)	0,111	2.7 (1.44 - 5.05)	0,032
PC.aa.C38.6	1.64 (0.8 - 3.36)	0,292	1.69 (0.84 - 3.37)	0,360	1.98 (1.02 - 3.83)	0,103
PC.aa.C40.0	1.91 (0.93 - 3.91)	0,170	1.06 (0.53 - 2.13)	0,921	1.59 (0.83 - 3.07)	0,236
PC.aa.C40.1	2.61 (1.3 - 5.26)	0,046	1.48 (0.75 - 2.91)	0,468	1.38 (0.73 - 2.62)	0,388
PC.aa.C40.2	2.69 (1.35 - 5.36)	0,038	1.56 (0.81 - 3.04)	0,414	1.93 (1.03 - 3.63)	0,103
PC.aa.C40.3	2.88 (1.44 - 5.75)	0,036	2.22 (1.14 - 4.32)	0,144	1.92 (1.02 - 3.63)	0,106
PC.aa.C40.4	3.34 (1.68 - 6.64)	0,018	3.1 (1.6 - 6.01)	0,047	2.34 (1.24 - 4.39)	0,060
PC.aa.C40.5	2.08 (1.06 - 4.1)	0,107	2.35 (1.23 - 4.49)	0,096	2.1 (1.13 - 3.91)	0,071
PC.aa.C40.6	2.08 (1.03 - 4.21)	0,120	1.72 (0.87 - 3.39)	0,316	2.1 (1.1 - 4)	0,075
PC.aa.C42.0	1.68 (0.84 - 3.34)	0,249	1.28 (0.66 - 2.51)	0,687	1.92 (1.02 - 3.59)	0,103
PC.aa.C42.5	1.86 (0.94 - 3.7)	0,169	1.45 (0.75 - 2.81)	0,483	1.82 (0.97 - 3.41)	0,127
PC.aa.C43.6	1.45 (0.72 - 2.92)	0,411	1.61 (0.82 - 3.15)	0,407	1.92 (1.01 - 3.62)	0,106
PC.aa.C44.12	1.75 (0.87 - 3.52)	0,216	1.54 (0.78 - 3.02)	0,433	2.22 (1.17 - 4.22)	0,064
PC.ae.C30.0	1.97 (0.99 - 3.93)	0,143	1.74 (0.9 - 3.38)	0,298	1.38 (0.73 - 2.6)	0,378
PC.ae.C32.0	1.83 (0.91 - 3.67)	0,187	1.67 (0.86 - 3.24)	0,345	1.66 (0.88 - 3.14)	0,189
PC.ae.C32.1	1.97 (0.97 - 3.99)	0,148	1.79 (0.91 - 3.51)	0,284	1.78 (0.93 - 3.4)	0,150
PC.ae.C32.2	1.58 (0.79 - 3.16)	0,304	1.16 (0.6 - 2.25)	0,798	1.39 (0.73 - 2.63)	0,376
PC.ae.C34.0	1.97 (0.98 - 3.93)	0,144	1.71 (0.88 - 3.33)	0,311	1.25 (0.66 - 2.36)	0,535
PC.ae.C34.1	1.65 (0.82 - 3.32)	0,267	1.51 (0.77 - 2.93)	0,443	1.74 (0.92 - 3.29)	0,152
PC.ae.C34.2	1.42 (0.7 - 2.89)	0,462	1.16 (0.58 - 2.31)	0,799	1.64 (0.85 - 3.16)	0,212
PC.ae.C34.3	1.46 (0.73 - 2.89)	0,400	1.14 (0.59 - 2.21)	0,814	1.47 (0.78 - 2.77)	0,300
PC.ae.C34.4	1.52 (0.75 - 3.07)	0,356	1.22 (0.62 - 2.4)	0,754	1.74 (0.91 - 3.31)	0,157
PC.ae.C36.0	1.9 (0.95 - 3.82)	0,165	1.94 (1 - 3.77)	0,227	2.09 (1.1 - 3.96)	0,075
PC.ae.C36.1	2.23 (1.11 - 4.45)	0,091	1.98 (1.02 - 3.86)	0,217	2.41 (1.28 - 4.53)	0,054
PC.ae.C36.2	1.15 (0.56 - 2.35)	0,773	1.05 (0.53 - 2.08)	0,932	1.19 (0.61 - 2.29)	0,639
PC.ae.C36.3	1.67 (0.83 - 3.36)	0,258	1.55 (0.79 - 3.04)	0,431	2.25 (1.19 - 4.26)	0,064
PC.ae.C36.4	2.8 (1.42 - 5.52)	0,036	2.63 (1.37 - 5.06)	0,052	2.38 (1.28 - 4.44)	0,054
PC.ae.C36.5	2.5 (1.27 - 4.91)	0,050	2.05 (1.07 - 3.94)	0,184	2.16 (1.16 - 4.02)	0,064
PC.ae.C38.0	1.27 (0.62 - 2.61)	0,602	1.51 (0.76 - 2.99)	0,461	1.83 (0.95 - 3.53)	0,134
PC.ae.C38.2	1.41 (0.69 - 2.89)	0,467	1.32 (0.66 - 2.62)	0,656	1.25 (0.65 - 2.42)	0,546
PC.ae.C38.3	2.04 (1 - 4.14)	0,133	2.48 (1.26 - 4.87)	0,093	2.1 (1.1 - 4)	0,075
PC.ae.C38.4	2.06 (1.04 - 4.06)	0,113	1.93 (1 - 3.7)	0,223	1.91 (1.02 - 3.57)	0,103
PC.ae.C38.5	2.18 (1.1 - 4.32)	0,093	2 (1.03 - 3.85)	0,208	2.37 (1.27 - 4.43)	0,054
PC.ae.C38.6	2.23 (1.11 - 4.49)	0,091	1.74 (0.89 - 3.42)	0,307	2.45 (1.3 - 4.64)	0,054
PC.ae.C40.0	1.99 (0.96 - 4.14)	0,154	1.91 (0.95 - 3.85)	0,248	1.59 (0.81 - 3.11)	0,247
PC.ae.C40.1	1.81 (0.87 - 3.73)	0,210	1.35 (0.67 - 2.72)	0,636	1.58 (0.81 - 3.07)	0,247
PC.ae.C40.2	1.62 (0.8 - 3.28)	0,291	1.16 (0.59 - 2.3)	0,798	1.92 (1.01 - 3.65)	0,109
PC.ae.C40.3	2.23 (1.1 - 4.54)	0,094	1.56 (0.78 - 3.09)	0,433	1.49 (0.77 - 2.86)	0,300
PC.ae.C40.4	1.94 (0.97 - 3.87)	0,150	1.44 (0.74 - 2.81)	0,496	1.67 (0.88 - 3.17)	0,189
PC.ae.C40.5	1.76 (0.88 - 3.54)	0,210	1.34 (0.68 - 2.61)	0,632	2.23 (1.18 - 4.24)	0,064
PC.ae.C40.6	1.4 (0.69 - 2.85)	0,467	1.25 (0.63 - 2.48)	0,715	1.97 (1.03 - 3.77)	0,103

PC.ae.C42.1	2.79 (1.37 - 5.7)	0,038	3.23 (1.63 - 6.4)	0,047	1.58 (0.82 - 3.04)	0,242
PC.ae.C42.3	1.07 (0.52 - 2.19)	0,908	0.68 (0.34 - 1.36)	0,487	1.26 (0.65 - 2.46)	0,535
PC.ae.C42.4	1.59 (0.79 - 3.21)	0,304	1.42 (0.73 - 2.78)	0,515	1.85 (0.98 - 3.5)	0,126
PC.ae.C42.5	1.59 (0.79 - 3.19)	0,304	1.19 (0.61 - 2.34)	0,775	1.93 (1.02 - 3.67)	0,106
PC.ae.C42.6	1.21 (0.6 - 2.45)	0,676	0.96 (0.49 - 1.9)	0,939	1.6 (0.84 - 3.04)	0,226
SM.a.C30.1	1.89 (0.94 - 3.81)	0,168	1.79 (0.92 - 3.48)	0,276	1.59 (0.85 - 3)	0,215
SM.a.C32.1	1.79 (0.88 - 3.62)	0,209	2.28 (1.17 - 4.43)	0,126	1.64 (0.86 - 3.11)	0,204
SM.a.C32.2	2.2 (1.06 - 4.59)	0,109	2.35 (1.18 - 4.65)	0,126	2.47 (1.28 - 4.76)	0,054
SM.a.C33.1	1.42 (0.69 - 2.9)	0,465	1.5 (0.76 - 2.96)	0,456	1.37 (0.72 - 2.64)	0,397
SM.a.C34.1	2.02 (0.99 - 4.12)	0,141	1.58 (0.8 - 3.12)	0,414	1.57 (0.82 - 3.01)	0,247
SM.a.C34.2	2.72 (1.28 - 5.76)	0,054	1.96 (0.96 - 3.98)	0,241	2.04 (1.03 - 4)	0,103
SM.a.C35.0	1.67 (0.83 - 3.35)	0,262	1.31 (0.67 - 2.55)	0,656	1.8 (0.95 - 3.4)	0,136
SM.a.C35.1	1.16 (0.56 - 2.39)	0,766	1.45 (0.73 - 2.87)	0,502	1.44 (0.75 - 2.78)	0,337
SM.a.C36.1	1.85 (0.89 - 3.82)	0,196	1.9 (0.96 - 3.79)	0,241	2.35 (1.22 - 4.53)	0,064
SM.a.C36.2	1.78 (0.84 - 3.79)	0,237	1.95 (0.96 - 3.98)	0,241	2.24 (1.13 - 4.42)	0,071
SM.a.C36.3	1.71 (0.81 - 3.64)	0,272	1.57 (0.77 - 3.18)	0,433	2.04 (1.04 - 4.01)	0,103
SM.a.C37.1	1.32 (0.64 - 2.69)	0,555	1.55 (0.79 - 3.04)	0,433	2.4 (1.27 - 4.55)	0,054
SM.a.C38.2	1.37 (0.67 - 2.8)	0,497	1.6 (0.82 - 3.14)	0,407	2.19 (1.15 - 4.18)	0,067
SM.a.C38.3	1.33 (0.66 - 2.69)	0,536	1.59 (0.82 - 3.08)	0,407	1.83 (0.97 - 3.46)	0,129
SM.a.C39.1	1.97 (0.95 - 4.1)	0,163	1.78 (0.89 - 3.56)	0,303	1.94 (1 - 3.76)	0,109
SM.a.C39.2	1.11 (0.53 - 2.32)	0,835	1.1 (0.55 - 2.2)	0,872	1.67 (0.86 - 3.23)	0,204
SM.a.C40.2	2 (0.99 - 4.07)	0,143	2.11 (1.08 - 4.13)	0,184	2.27 (1.2 - 4.32)	0,064
SM.a.C40.5	2.03 (1.02 - 4.05)	0,124	1.91 (0.98 - 3.7)	0,241	3.38 (1.81 - 6.32)	0,009
SM.a.C41.1	1.95 (0.97 - 3.92)	0,148	1.89 (0.98 - 3.66)	0,241	2.14 (1.15 - 4)	0,067
SM.a.C41.2	1.57 (0.77 - 3.18)	0,318	1.48 (0.76 - 2.9)	0,465	2.12 (1.12 - 4)	0,071
SM.a.C42.1	2.03 (1.02 - 4.04)	0,123	1.26 (0.65 - 2.43)	0,705	1.7 (0.91 - 3.18)	0,163
SM.a.C42.2	2.16 (1.06 - 4.43)	0,109	1.24 (0.63 - 2.47)	0,729	2.14 (1.11 - 4.1)	0,073
SM.a.C42.3	2.11 (1.03 - 4.32)	0,117	1.57 (0.79 - 3.11)	0,423	2.15 (1.12 - 4.14)	0,071
SM.a.C42.4	2.67 (1.34 - 5.31)	0,038	2.78 (1.44 - 5.37)	0,049	2.73 (1.45 - 5.13)	0,032
SM.a.C42.6	1.99 (0.97 - 4.07)	0,148	2.02 (1.02 - 4)	0,217	2.24 (1.17 - 4.32)	0,066
SM.a.C43.1	2.34 (1.15 - 4.75)	0,085	1.18 (0.6 - 2.34)	0,776	1.56 (0.82 - 2.99)	0,247
SM.a.C43.2	2.17 (1.07 - 4.37)	0,103	1.84 (0.94 - 3.62)	0,254	1.46 (0.77 - 2.77)	0,311
SM.a.C44.6	1.84 (0.9 - 3.73)	0,193	1.4 (0.71 - 2.75)	0,556	2.3 (1.21 - 4.38)	0,064
SM.e.C36.2	1.63 (0.78 - 3.42)	0,304	1.27 (0.63 - 2.56)	0,709	1.54 (0.78 - 3.02)	0,283
SM.e.C38.3	1.26 (0.63 - 2.52)	0,608	1.32 (0.68 - 2.57)	0,636	2.12 (1.13 - 4)	0,071
SM.e.C40.5	1.7 (0.86 - 3.36)	0,221	1.56 (0.81 - 3.01)	0,414	2.14 (1.15 - 3.97)	0,067
Carn	1.5 (0.74 - 3.04)	0,377	1.28 (0.65 - 2.49)	0,691	2.26 (1.2 - 4.26)	0,064
Carn.a.C10.0	2.22 (1.12 - 4.42)	0,091	1.24 (0.63 - 2.41)	0,729	1.65 (0.87 - 3.1)	0,199
Carn.a.C10.1	1.64 (0.82 - 3.31)	0,276	1.03 (0.53 - 2.03)	0,939	1.91 (1.01 - 3.62)	0,109
Carn.a.C12.0	1.72 (0.86 - 3.41)	0,221	1.17 (0.61 - 2.28)	0,778	1.93 (1.03 - 3.63)	0,103
Carn.a.C14.1	1.39 (0.7 - 2.76)	0,467	0.84 (0.43 - 1.63)	0,775	1.88 (1 - 3.52)	0,109
Carn.a.C14.2	1.09 (0.55 - 2.16)	0,850	0.91 (0.47 - 1.75)	0,872	1.79 (0.96 - 3.34)	0,131
Carn.a.C15.0	1.19 (0.6 - 2.37)	0,694	1.03 (0.54 - 1.99)	0,939	1.5 (0.8 - 2.81)	0,278
Carn.a.C16.0	1.36 (0.69 - 2.68)	0,488	1.51 (0.79 - 2.92)	0,433	1.44 (0.77 - 2.69)	0,318
Carn.a.C16.0.Oxo	1.16 (0.58 - 2.33)	0,758	1 (0.51 - 1.97)	0,998	1.55 (0.82 - 2.96)	0,247

Carn.a.C16.1	1.03 (0.52 - 2.06)	0,956	1.01 (0.52 - 1.96)	0,979	1.89 (1 - 3.56)	0,109
Carn.a.C16.2	0.76 (0.39 - 1.5)	0,540	0.84 (0.44 - 1.61)	0,775	1.59 (0.86 - 2.96)	0,212
Carn.a.C18.0	0.89 (0.45 - 1.76)	0,804	0.66 (0.35 - 1.28)	0,438	1.05 (0.56 - 1.96)	0,889
Carn.a.C18.1	1.39 (0.69 - 2.77)	0,468	1.07 (0.55 - 2.09)	0,904	2.09 (1.11 - 3.94)	0,072
Carn.a.C18.2	1.27 (0.65 - 2.51)	0,585	1.09 (0.57 - 2.11)	0,872	1.73 (0.93 - 3.22)	0,151
Carn.a.C18.2.OH	1.07 (0.55 - 2.11)	0,883	0.92 (0.48 - 1.75)	0,872	1.19 (0.64 - 2.22)	0,619
Carn.a.C2.0	2.36 (1.18 - 4.69)	0,074	1.58 (0.82 - 3.07)	0,407	3.16 (1.68 - 5.94)	0,009
Carn.a.C20.0	1.56 (0.78 - 3.11)	0,311	1.84 (0.96 - 3.55)	0,241	1.31 (0.7 - 2.46)	0,459
Carn.a.C20.1	1.29 (0.65 - 2.53)	0,566	0.98 (0.51 - 1.87)	0,971	1.59 (0.86 - 2.95)	0,212
Carn.a.C20.3	1.13 (0.58 - 2.22)	0,790	1.14 (0.6 - 2.16)	0,819	1.22 (0.66 - 2.26)	0,569
Carn.a.C20.4	1.4 (0.71 - 2.76)	0,457	1.18 (0.62 - 2.27)	0,776	1.46 (0.78 - 2.72)	0,301
Carn.a.C3.0	1.79 (0.9 - 3.53)	0,195	1.31 (0.68 - 2.52)	0,640	1.79 (0.96 - 3.33)	0,131
Carn.a.C3.0.DC	1.03 (0.51 - 2.05)	0,958	0.89 (0.45 - 1.73)	0,843	1.27 (0.67 - 2.4)	0,516
Carn.a.C4.0	1.84 (0.94 - 3.63)	0,169	1.37 (0.71 - 2.64)	0,557	1.75 (0.94 - 3.25)	0,142
Carn.a.C5.0	1.92 (0.97 - 3.79)	0,148	1.64 (0.85 - 3.15)	0,362	1.85 (0.99 - 3.45)	0,116
Carn.a.C6.0	2.27 (1.14 - 4.53)	0,087	1.26 (0.65 - 2.47)	0,705	1.54 (0.82 - 2.92)	0,247
Carn.a.C6.0.OH	1.39 (0.69 - 2.8)	0,468	1.16 (0.59 - 2.27)	0,798	1.75 (0.93 - 3.32)	0,152
Carn.a.C8.0	1.8 (0.9 - 3.6)	0,195	1.21 (0.62 - 2.35)	0,775	1.31 (0.69 - 2.48)	0,459
Carn.a.C8.1	1.64 (0.81 - 3.33)	0,284	1.63 (0.83 - 3.22)	0,402	1.96 (1.03 - 3.74)	0,103
Carn.a.C9.0	2.19 (1.11 - 4.35)	0,091	1.1 (0.57 - 2.14)	0,872	1.42 (0.76 - 2.67)	0,337
Asn/Asp	0.37 (0.19 - 0.72)	0,038	0.58 (0.3 - 1.1)	0,284	0.54 (0.29 - 0.99)	0,109
Gln/Glu	0.58 (0.29 - 1.15)	0,216	0.51 (0.26 - 0.98)	0,217	0.31 (0.17 - 0.58)	0,009
NEFA18.1/NEFA18.0	0.99 (0.49 - 1.99)	0,978	0.85 (0.43 - 1.65)	0,776	1.55 (0.82 - 2.93)	0,247
NEFA16.1/NEFA16.0	1.06 (0.52 - 2.17)	0,914	1.27 (0.64 - 2.52)	0,705	1.21 (0.63 - 2.32)	0,598
PC.aa/PC.ae	1.24 (0.62 - 2.5)	0,627	1.48 (0.76 - 2.9)	0,467	1.4 (0.74 - 2.65)	0,365
Lyso.PC.a/PC.aa	0.68 (0.33 - 1.38)	0,408	1.09 (0.56 - 2.12)	0,875	1.23 (0.65 - 2.32)	0,559
(lyso.PC.a.C16.0 + lyso.PC.a.C18.0)/ PC.aa	0.61 (0.3 - 1.26)	0,292	1.3 (0.66 - 2.55)	0,663	1.42 (0.75 - 2.71)	0,346
(lyso.PC.a.C18.1 + lyso.PC.a.C18.2)	0.76 (0.38 - 1.53)	0,548	0.65 (0.34 - 1.25)	0,423	0.8 (0.43 - 1.49)	0,521
Carn.a.C.16.0/ free Carn	1.09 (0.55 - 2.18)	0,850	1.31 (0.68 - 2.53)	0,640	0.88 (0.47 - 1.64)	0,704
Carn.a.C2.0/ Carn.a.C16.0	1.37 (0.69 - 2.73)	0,478	0.9 (0.46 - 1.74)	0,858	1.5 (0.8 - 2.8)	0,278

Values represent absolute differences in blood pressure (95% confidence interval) and corresponding p-values from linear regression models that reflect the difference in blood pressure (mmHg) per SDS increase in maternal early-pregnancy metabolite concentrations (μmol/L) or metabolite ratio. Model includes gestational age at time of measurement, age, parity, pre-pregnancy body mass index, educational level, smoking and folic acid supplementation. AA amino acids, NEFA non-esterified fatty acids, PC.aa diacyl-phosphatidylcholines, PC.ae acyl-alkyl-phosphatidylcholines, lyso.PC.a acyl-lysophosphatidylcholines, lyso.PC.e alkyl-lysophosphatidylcholines, Carn.a acyl-carnitines, SM sphingomyelins.
[a] p-value corrected for multiple hypothesis testing using Benjamin-Hochberg FDR correction.
*****Statistically significant

Table S10. Lasso regression on systolic blood pressure change from first to third trimester

Variable	Coefficient
AGE_M_v2	0.20
BMI_0	-0.62
PARITY	-0.10
Smoke2cat	-0.94
Asp	1.59
His	0.19
Phe	-1.14
Trp	-0.45
Cys	0.37
NEFA_14_1	0.16
NEFA_16_0	-0.49
NEFA_16_1	0.48
NEFA_17_1	-0.67
NEFA_22_3	0.98
NEFA_22_5	-0.11
NEFA_22_6	-0.23
NEFA_24_4	-0.18
NEFA_26_1	0.14
Lyso.PC.a.C16.0	0.22
Lyso.PC.a.C16.1	0.67
Lyso.PC.a.C18.1	-0.90
Lyso.PC.a.C18.2	-0.12
Lyso.PC.a.C20.3	0.10
PC.aa.C30.0	-0.06
PC.aa.C32.0	-0.97
PC.aa.C34.4	-1.63
PC.aa.C34.5	-0.14
PC.aa.C36.5	-1.36
PC.aa.C38.3	0.03
PC.aa.C38.5	1.82
PC.aa.C40.2	-0.32
PC.ae.C36.4	0.39
SM.a.C40.5	1.59
Asn/asp	0.64
Gln/glu	-0.27

Lasso regression on the outcome systolic blood pressure change from first to third trimes ter. including all early-pregnancy metabolites and the selected confounders age. pre-preg nancy body-mass index. parity and smoking. that could not be penalized.

Table S11. Lasso on diastolic blood pressure change from first to third trimester

Variable	Coefficient
AGE_M_v2	0.21
BMI_0	-0.62
PARITY	-0.68
Smoke2cat	-0.92
Arg	0.00
Asn	0.03
Gly	-0.01
Lys	0.04
Phe	-0.07
Trp	-0.04
NEFA_15_0	-0.45
NEFA_17_2	-8.18
NEFA_18_0	0.00
NEFA_18_2	-0.01
NEFA_26_0	-0.17
lyso.PC.a.C18.1	-0.18
lyso.PC.a.C18.2	0.00
lyso.PC.e.C18.0	1.36
PC.aa.C34.4	-0.54
PC.aa.C40.0	-0.22
PC.aa.C40.2	-2.94
PC.aa.C40.5	0.14
PC.ae.C30.0	-0.45
PC.ae.C32.2	-3.50
PC.ae.C36.1	0.07
PC.ae.C36.5	-0.07
PC.ae.C40.5	0.13
SM.a.C32.2	0.83
SM.a.C35.0	3.40
SM.a.C35.1	0.19
SM.a.C37.1	0.02
SM.a.C39.2	0.99
SM.a.C40.5	0.47
SM.a.C41.1	-0.07
SM.a.C42.3	0.06
SM.a.C43.2	-0.43
SM.a.C44.6	0.03
SM.e.C40.5	-1.06
Carn	0.13
Carn.a.C10.0	8.46
Carn.a.C15.0	6.27
Carn.a.C16.1	36.69
Carn.a.C16.2	20.98
Carn.a.C20.0	-109.26
Carn.a.C20.3	-4.75
Carn.a.C4.0	-2.38
Carn.a.C6.0.OH	33.44
Asn/asp	0.18
Gln/glu	-0.40
Lasso regression on the outcome systolic blood pressure change from first to third trimes ter. including all early-pregnancy metabolites and the selected confounders age. pre-preg nancy body-mass index. parity and smoking. that could not be penalized.	

Figure S1. Flowchart of participants included in the study.

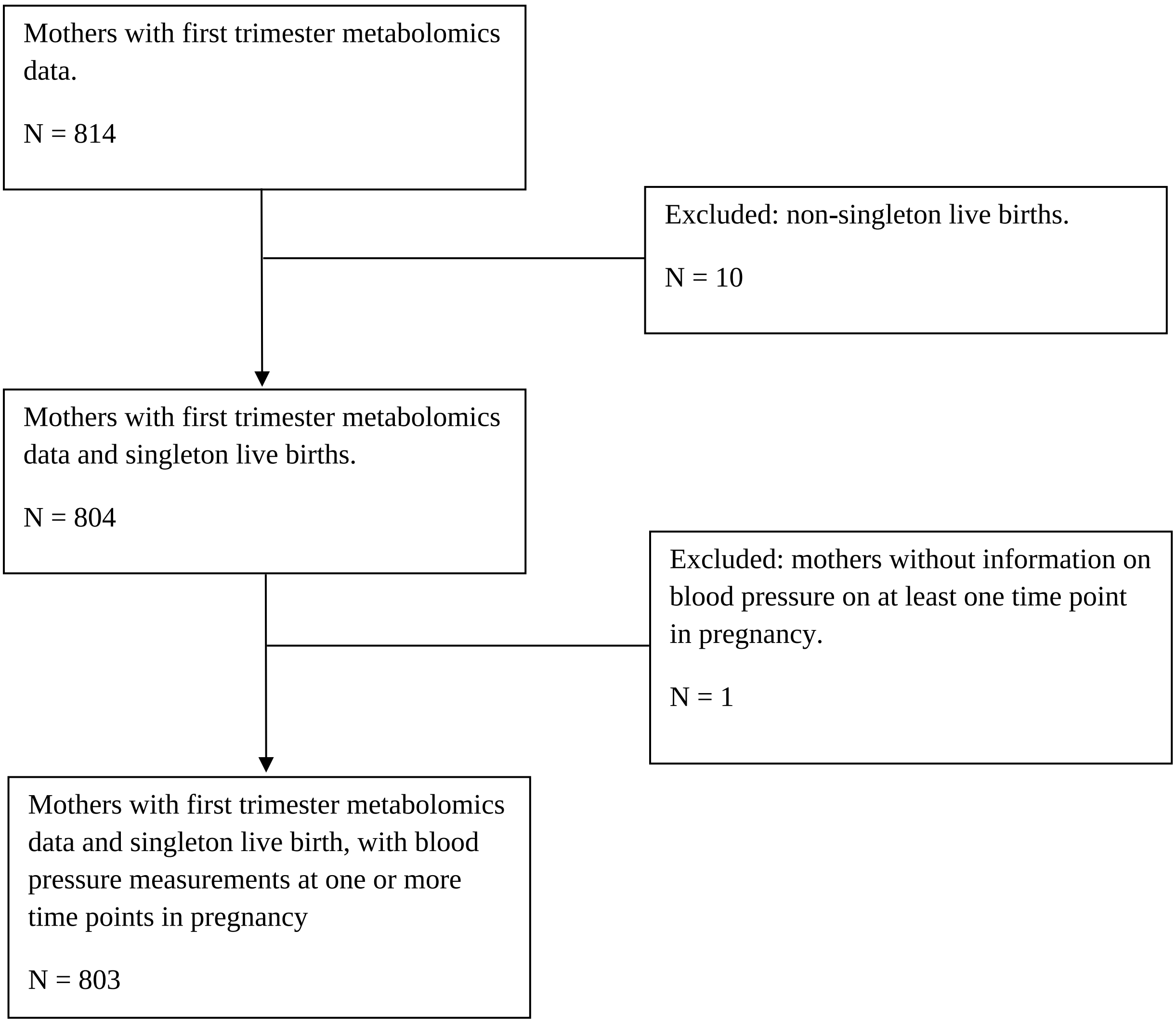

Figure S2. Direct Acyclic Graph

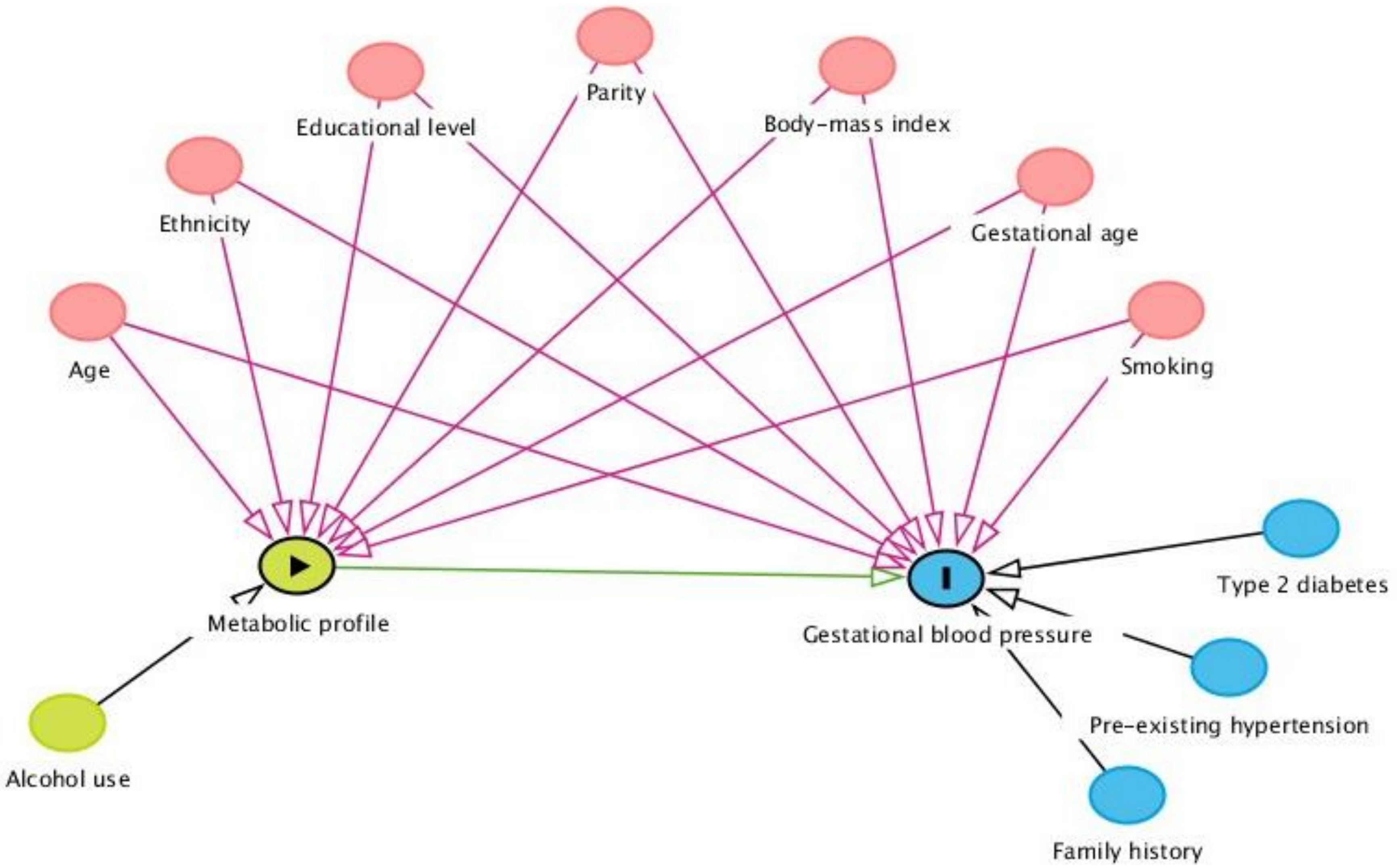

Figure S3. ROC curves on the prediction of gestational hypertensive disorders

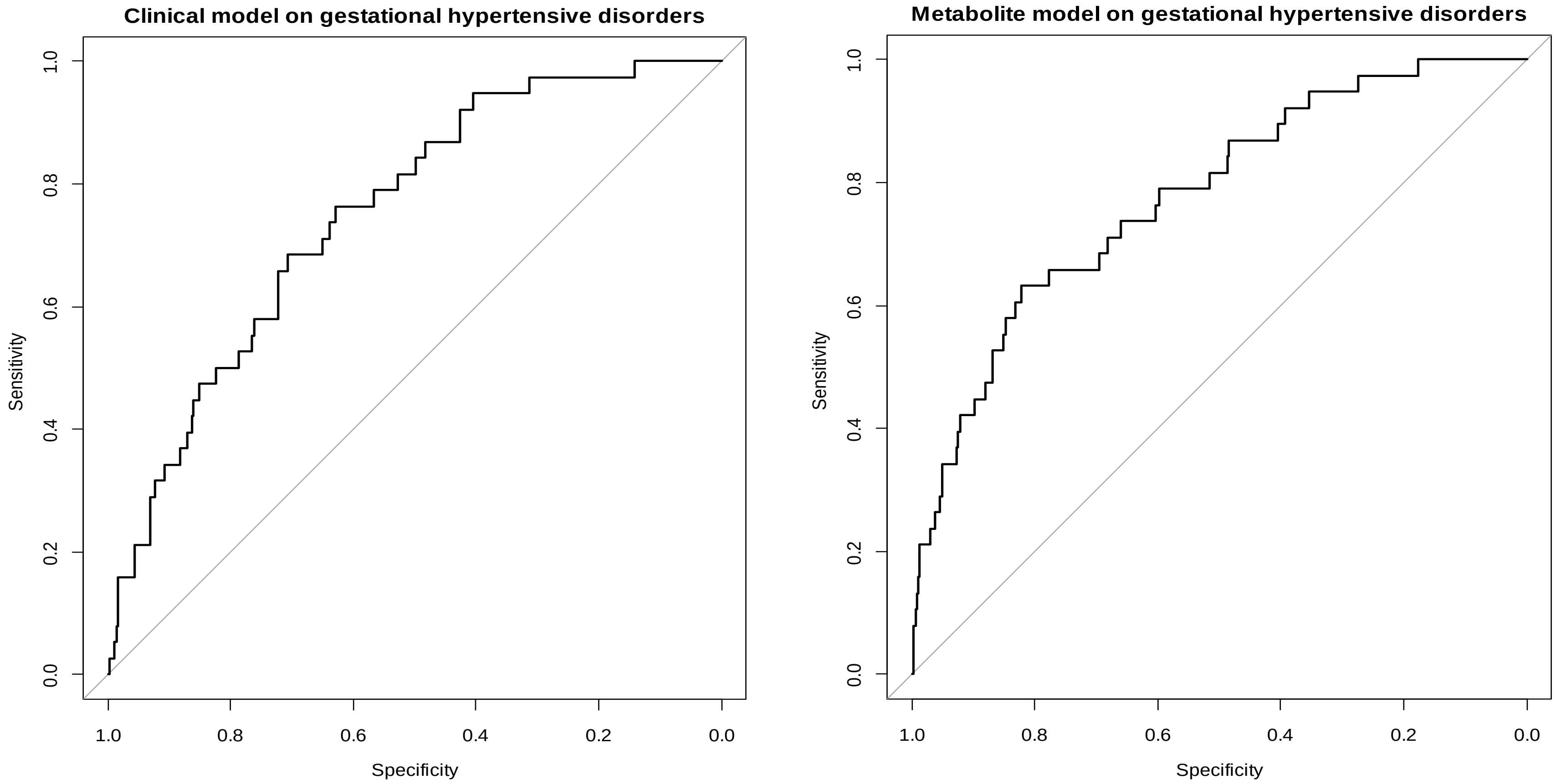

Clinical models includes maternal age. pre-pregnancy BMI. parity and smoking. Metabolite model includes maternal age. pre-pregnancy BMI. parity. smoking. and arginine. asparagine. glycine. lysine. tryptophan. NEFA_18_0. NEFA_26_0. PC.aa.C34.4. PC.ae.C36.5. SM.a.C37.1. SM.a.C38.2. SM.a.C39.2. SM.a.C41.1. SM.a.C43.2. Carn.a.C16.1. Carn.a.C16.2 and Arganine/asparagine ratio.
